# Manganese(II) and Magnesium(II) Analogies in Iridium Hydride Coordination Chemistry

**DOI:** 10.1021/acs.inorgchem.6c01844

**Published:** 2026-06-12

**Authors:** Till Neumann, Erwann Jeanneau, Chloé Thieuleux, Clément Camp

**Affiliations:** † Laboratory of Catalysis, Polymerization, Processes and Materials (CP2M, UMR 5128) CNRS, Institut de Chimie de Lyon, 27098 Universite Claude Bernard Lyon 1, CPE Lyon, 3 rue Victor Grignard, Villeurbanne 69616, France; ‡ Centre de Diffractométrie Henri Longchambon, Universite Claude Bernard Lyon 1, 5 rue de la Doua, Villeurbanne 69100, France

## Abstract

Alkane elimination reactions between stable magnesium and manganese dialkyls, [L_*n*_M­(CH_2_ *t*Bu)_2_] (M = Mg, Mn), and an iridium polyhydride species, [Cp*IrH_4_] (Cp* = C_5_Me_5_), provide a variety of heterobimetallic Mg–Ir and Mn–Ir hydride complexes. In several cases, their solid-state molecular structures are shown to be virtually identical despite fundamentally different electronic properties of Mg­(II) (2*p* ^6^) and Mn­(II) (3*d* ^5^). A direct comparison of the paramagnetic Mn with the diamagnetic Mg analog enables a comprehensive characterization based on a combination of analytical techniques (single-crystal X-ray diffraction, IR, and NMR spectroscopies), especially with respect to the hydride ligands, traditionally difficult to identify in open-shell species. Nevertheless, such congruence is not universal: the nuclearity and M:Ir stoichiometry depend on the nature of the Mg and Mn dialkyl precursors, which in turn is determined by the type of coligand L, and on the presence of proton-donating reagents in the reaction mixture, revealing subtle differences between the Mg and Mn systems in certain cases.

## Graphical abstract

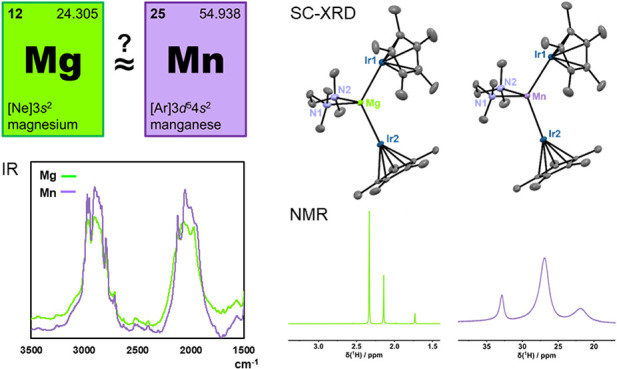

## Introduction

Manganese coordination chemistry is currently attracting considerable attention due to the element’s earth abundance, low toxicity, and remarkable catalytic potential. However, Mn­(II) organometallic compounds remain comparatively underexplored and are often considered atypical within the 3d series, as manganese­(II) coordination compounds often show an unusual behavior compared to other first-row transition metals.^1^ This becomes particularly obvious in series of structurally similar compounds containing Mn­(II) and other 3*d* transition-metal ions in the +II oxidation state, such as the metallocenes [MCp_2_][Bibr ref2] or some recently reported perhydrocarbyl-stabilized Ta–M bimetallic complexes.^3^ The increased ionic character of Mn­(II)–C bonds frequently results in a reduced influence of the 18-electron rule, and, as a result, their structural properties and reactivity rather correspond to organomagnesium compounds.^4^ The unique nature of Mn­(II) and its similarity with Mg­(II) are reflected by an exceptional number of stable aryl and alkyl complexes, starting from the description of [PhMnI] and [Ph_2_Mn] by Gilman^5,6^ and followed by the development of closely related Mg and Mn dialkyls bearing neopentyl (CH_2_ *t*Bu), neosilyl (CH_2_SiMe_3_), and neophyl (CH_2_CMe_2_Ph) ligands lacking hydrogen atoms at the β-position by Wilkinson and coworkers,^7,8^ completed by more detailed structural investigations later on.^9–12^ Mulvey and coworkers then demonstrated that both Mg­(II) and Mn­(II) partake in the alkali-metal-assisted C–H metalation of arenes and the formation of base-stabilized “inverse crown” complexes (Scheme 1a).^13–15^ More recently, the same group described polymeric structures obtained from a reaction of Mg­(II) and Mn­(II) arylsilyl amido complexes^16,17^ with heavy alkali-metal alkyls (Scheme 1b).^18,19^ Finally, Uzelac and coworkers directly compared Mg­(II) and Mn­(II) complexes as catalysts for the hydroamination of styrene derivatives and highlighted that their structural resemblance causes almost identical reactivity (Scheme 1c).^20^

**Scheme 1:**
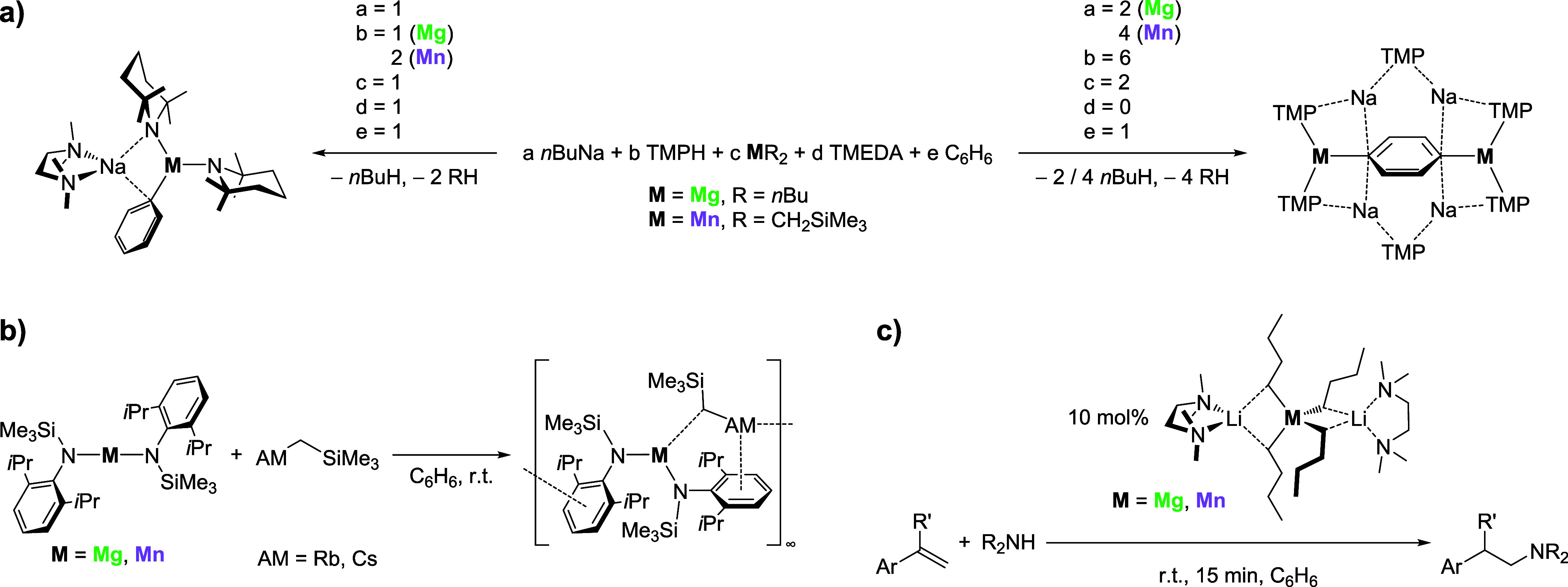
Examples of Analogies between Similar Magnesium­(II) and Manganese­(II) Organometallic Compounds: (a) Alkali-Metal-Mediated C–H Magnesiation and Manganation of Arenes,^13–15^ (b) Synthesis of Polymeric Alkali Metal Bis­(amido)­alkyl Magnesiates and Manganates,^18,19^ (c) Hydroamination of Styrenes Catalyzed by Isostructural Lithium Tetra­(*n*-butyl) Magnesiates and Manganates^20^ [Fn sch1-fn1]

i Adapted with permission from the original publications: ref [Bibr ref15], 2007 Wiley-VCH Verlag GmbH; ref 19, copyright 2024 American Chemical Society; ref 20, available under a CC-BY 3.0 license, copyright 2025 Jones, R. S.; Krämer, T.; Nichol, G. S.; Mahon, M. F.; Kragskow, J. G. C.; Uzelac, M. A.

The present work makes use of dineopentylmagnesium and dineopentylmanganese (either uncoordinated or several of their adducts with coordinating ligands) to access heterobimetallic complexes associating Mg and Mn with Ir. The choice of Ir is founded on the availability of a versatile polyhydride precursor, [Cp*IrH_4_] (Cp* = C_5_Me_5_), which possesses an elevated Brønsted acidity. As a result, [Cp*IrH_4_] undergoes protonolysis reactions with basic X-type ligands and therefore enables the facile construction of heterobimetallic M–Ir hydride architectures from a diversity of metal species (Scheme 2). Numerous examples combining main-group,^21–28^ transition-metal,^29–36^ or lanthanide/actinide^37–40^ complexes featuring alkyl or amido ligands and rhenium, osmium, or iridium polyhydrides have been reported by our group and others. However, for different reasons, paramagnetic transition-metal hydride complexes remain relatively rare and sparsely characterized^41^ even though they play key roles in homogeneous catalysis^42^ and enzymes,^43,44^ to name just a few examples. The alkane elimination approach using [Cp*IrH_4_] offers a straightforward pathway toward open-shell Mn–Ir hydride species and their closed-shell Mg–Ir analogs. In several cases, their structural properties (at least in the solid state) are shown to be nearly identical, a fact which facilitates the characterization of the paramagnetic complexes by means of a direct comparison with their diamagnetic counterparts relying on common analytical techniques.

**Scheme 2:**
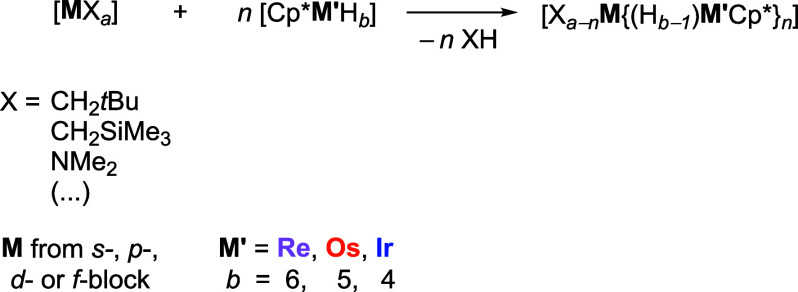
General Synthetic Strategy for Preparing Hydride-Rich Heterobimetallic Complexes from M′ Polyhydride Precursors (M′ = Re, Os, Ir)

Yet, such congruence is not universal, and differences in structure and behavior between the Mg and Mn derivatives can emerge for a variety of reasons, as shown in the second part of the manuscript. Note that there is a growing interest in heterobimetallic hydride complexes owing to their unusual structures and distinctive bonding situations,^45–48^ as exemplified by the expanding family of Mg–H–TM (TM = transition metal) systems.^21,49–55^ However, analogous Mn–H–TM systems remain rare and limited to low-spin Mn­(I) carbonyl complexes,^56–60^ a disparity that may in part reflect the greater challenges associated with the reliable preparation and characterization of high-spin Mn­(II)–H–TM compounds. More generally, molecular heterobimetallic systems are attracting increasing attention in catalysis, as metal–metal cooperativity can enable unique activation pathways and enhanced catalytic performance.^61–69^ In particular, polar heterobimetallic complexes involving electropositive metals such as Mg have been shown to display synergistic reactivity patterns that are inaccessible to the corresponding monometallic fragments.^61,64^ Related examples involving Mn remain comparatively scarce.^70^ In this context, the compounds reported herein not only provide insight into the structural and spectroscopic parallels between Mg and Mn complexes but also expand the scope of heterobimetallic Mg/Mn chemistry and may offer promising platforms for the future development of cooperative reactivity and catalytic transformations.

## Results and Discussion

The treatment of the dineopentyl compounds [Mg­(CH_2_ *t*Bu)_2_(1,4-dioxane)]_*n*_ and [Mn­(CH_2_tBu)_2_]_4_ with a stoichiometric amount of *N*,*N*,*N*′,*N*′-tetramethylethylenediamine (TMEDA) in a pentane solution at room temperature affords the monomeric chelated dineopentylmetal complexes [M­(tmeda)­(CH_2_ *t*Bu)_2_] (**1-M**, M = Mg, Mn; Scheme 3 top). Both are isolable as crystalline solids (75 and 82% yields for **1-Mg** and **1-Mn**, respectively). **1-Mg** and **1-Mn** readily react with two equivalents of the (pentamethylcyclopentadienyl)­iridium hydride [Cp*IrH_4_] to give the trinuclear heterobimetallic complexes [M­(tmeda)­(IrH_3_Cp*)_2_] (**2-M**, M = Mg, Mn, 85 and 69% yields for **2-Mg** and **2-Mn**, respectively). Like in previous examples of reactions between metal complexes featuring basic alkyl, aryl, or amido ligands and late transition-metal polyhydrides (references in the Introduction), the protonolysis of the neopentyl ligands in **1-M** by the Brønsted-acidic iridium precursor leads to the elimination of neopentane, C­(CH_3_)_4_ (“NpH”), detected by ^1^H NMR monitoring of the reaction. The reaction results in the formation of the bimetallic product **2-M**, in which the metal centers are bridged by part of the hydride ligands. These hydride ligands play an important role in the reactivity of **2-M**. The addition of one equivalent of the sterically demanding tris­(*tert*-butoxy)­silanol, (*t*BuO)_3_SiOH, to **2-M** provokes the reprotonation of a [Cp*Ir­(μ-H)_3_] fragment and generates [M­{OSi­(O*t*Bu)_3_}­(tmeda)­(IrH_3_Cp*)] (**3-M**, M = Mg, Mn, 72 and 84% yields for **3-Mg** and **3-Mn**, respectively) with the release of [Cp*IrH_4_] into the reaction mixture, as evidenced by a ^1^H NMR monitoring of the reaction. The *in situ* addition of a second equivalent of (*t*BuO)_3_SiOH to **2-M** (or of one equivalent of the silanol to the isolated compounds **3-M**) results in the reprotonation of the remaining Cp*IrH_3_ fragment, again releasing Cp*IrH_4_ and affording the new complexes [M­(tmeda)­(OSi­{O*t*Bu}_3_)_2_] (M = Mg, Mn; see SI for characterization details). Their crystal structures closely resemble those of the analogous Cr and Co complexes reported previously.^71,72^ Interestingly, **2-M** does not only react as a Brønsted base but also as a hydride donor: the reaction with the carbenium salt [Ph_3_C]­[B­(C_6_F_5_)_4_] results in the cationic, hydride-bridged Cp* iridium dimer [Cp*IrH_3_IrCp*]­[B­(C_6_F_5_)_4_] (**4**) stabilized by the weakly coordinating anion (Scheme 3 bottom). The cationic fragment of this compound has been described^73,74^ but not structurally characterized until recently.^34^ The structure and NMR spectra of **4** are therefore given in the SI (Figures S16, S17, and S49). They are not further discussed because the hydride abstraction unintentionally causes the dissociation from the second metal (Mg, Mn) instead of yielding a modified bimetallic complex, making this synthetic route of limited interest.

**Scheme 3:**
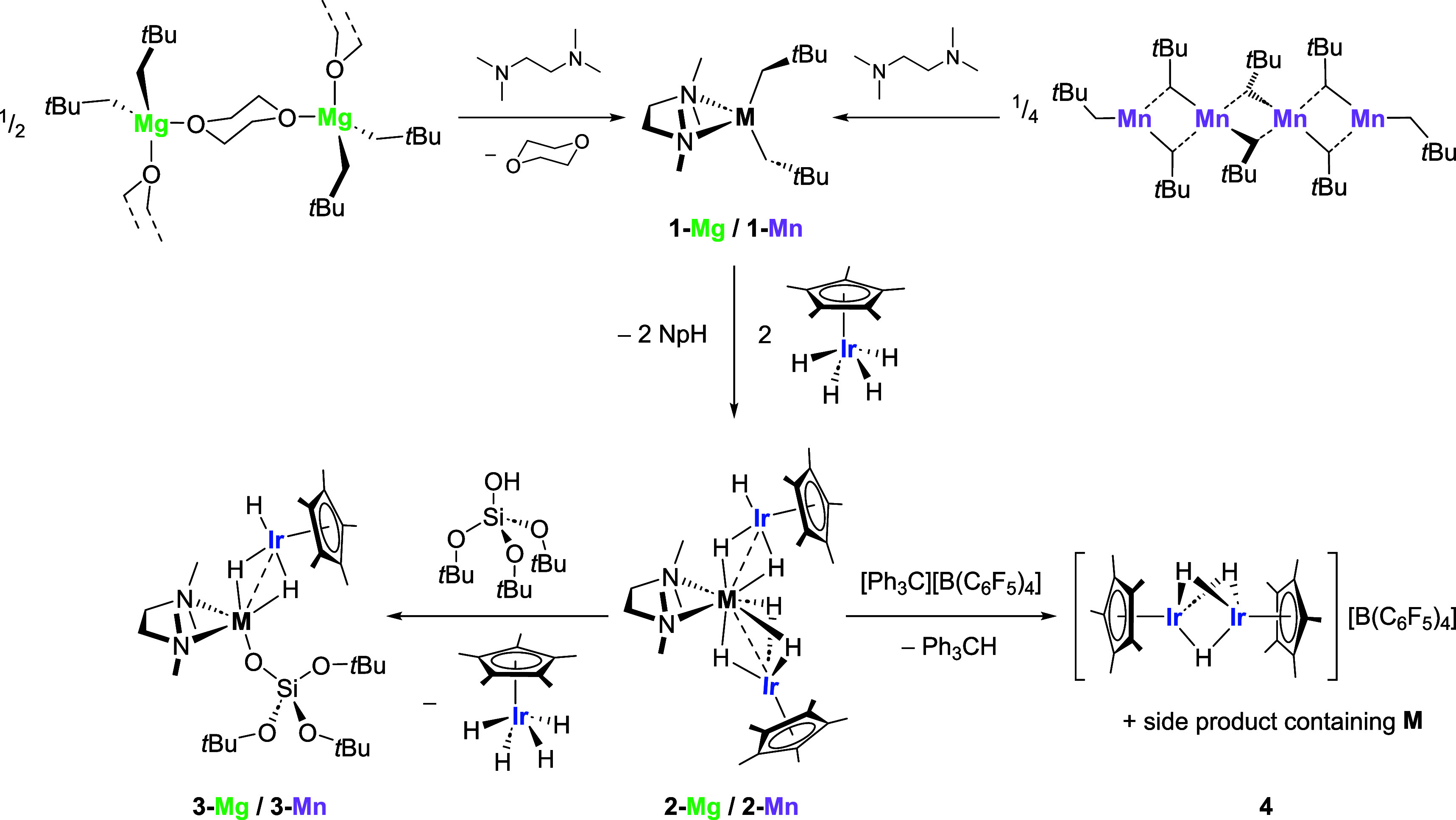
Syntheses of Isostructural Magnesium–Iridium and Manganese–Iridium Compounds and Reactivity toward Proton-Donating and Hydride Abstraction Reagents

**Scheme 4:**
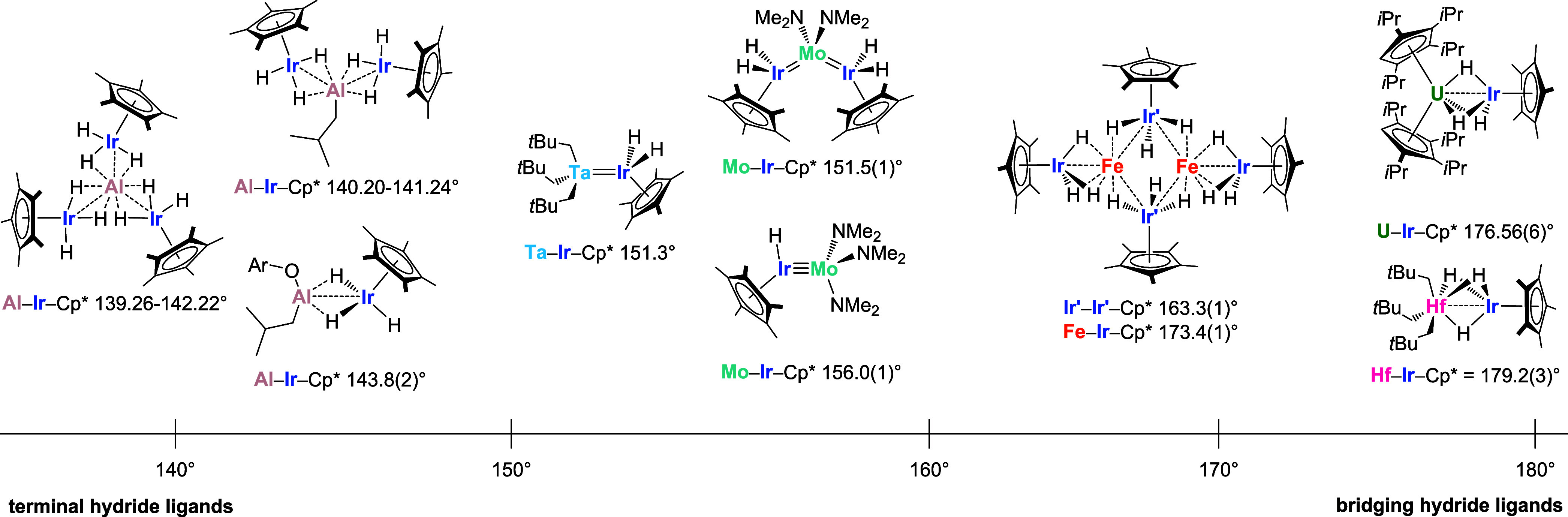
Examples of Heterobimetallic Complexes Obtained from [Cp*IrH_4_] with Different Configurations of Bridging and Terminal Hydride Ligands and Associated M···Ir···Cp* Angles, Reproduced from the Original Publications.^22,23,29,30,33,34,40^

All compounds represented in Scheme 3 could be obtained in single-crystal quality and analyzed by X-ray diffraction (XRD), solution-state nuclear magnetic resonance (NMR), and diffuse reflectance infrared Fourier-transform (DRIFT) spectroscopy. The main challenges relating to their characterization include the paramagnetism of the Mn­(II) species **1-Mn**, **2-Mn**, and **3-Mn** causing broad resonances in the corresponding NMR spectra as well as the difficulty of localizing hydride ligands next to heavy metal atoms (Ir) in the difference Fourier maps of the XRD refinement models despite high-quality crystallographic datasets. A combined discussion of all three analytical techniques (XRD, NMR, and DRIFT) is required to fully understand the structural properties of the compounds **1-Mn**, **2-Mn**, and **3-Mn** and relies on the comparison with the diamagnetic complexes **1-Mg**, **2-Mg**, and **3-Mg**. Indeed, the magnesium compounds are found to be isostructural with their paramagnetic manganese analogs, a circumstance that supports the proposed analogy and facilitates the structural elucidation of **1-Mn**, **2-Mn**, and **3-Mn** owing to sharp and easily interpretable ^1^H and ^13^C NMR resonances in the spectra of **1-Mg**, **2-Mg**, and **3-Mg**.

Although the syntheses of complexes **1-M** were already described by Wilkinson and coworkers,^7,8^ to the best of our knowledge, their crystal structures have not been reported so far. They are shown in Figure 1 and closely resemble that of [Mn­(tmeda)­(CH_2_SiMe_3_)_2_] described by Mulvey and coworkers.^10^ Both compounds crystallize in the same space group (*C*2/*c*, no. 15). Their unit cell parameters differ by less than 0.06 Å in any direction of space, and the β angles lie within 0.2° (Table S1), giving a first indication of the great structural similarity between analogous magnesium and manganese complexes. In spite of considerably different Pauling radii (Mg: 1.364 Å, Mn: 1.168 Å^75^), the bond distances and angles around the Mg and Mn centers in **1-M** are nearly identical. The Mg*–*C bonds are slightly longer (2.1637(9) Å) and the C*–*Mg*–*C angle is slightly sharper (138.09(5)°) than the Mn–C bonds (2.1556(8) Å) and the C–Mn–C angle (142.81(4)°), while the opposite is true for the M–N contacts (2.2795(8) Å for Mg vs 2.3470(7) Å for Mn) and N–M–N angles (81.57(4)° for Mg *vs* 79.50(3)° for Mn). Their NMR (Figures S1–S3) and infrared spectra (Figures S37 and S38) are given in the SI.

**Figure 1:**
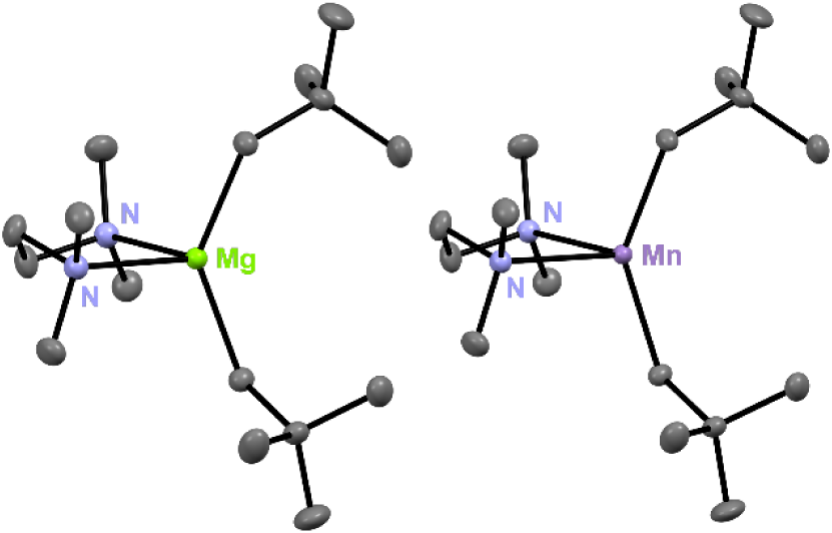
Solid-state molecular structures of **1-Mg** (left) and **1-Mn** (right) with thermal ellipsoids (ORTEP) drawn at the 50% probability level. Carbon atoms (gray) are unlabeled, and hydrogen atoms are omitted for clarity. Selected bond distances (Å) and angles (°): Mg–C 2.1637(9), Mn–C 2.1556(8), Mg–N 2.2795(8), Mn–N 2.3470(7); Mg–C–*t*Bu 125.20(6), Mn–C–*t*Bu 123.39(6), N–Mg–N 81.57(4), N–Mn–N 79.50(3), C–Mg–C 138.09(5), C–Mn–C 142.81(4).

Figure 2 depicts the crystal structures of the heterobimetallic compounds **2-M** and **3-M** (M = Mg, Mn). Key distances and angles around the metal centers and ligand donor atoms are summarized in Table 1. **2-Mg** and **2-Mn** crystallize in the space group *Pna*2_1_ (no. 33), and **3-Mg** and **3-Mn** in the space group *P*-1 (no. 2). Again, the unit cell parameter variations within these two pairs of structures are negligible (<0.05 Å in any direction of space, cell angles differing by less than 0.03°, Tables S2 and S3). The metal centers in all four complexes exhibit distorted tetrahedral coordination geometries with hardly varying N–M–N bite angles between 79.6(2)° (**2-Mn**) and 82.11(6)° (**3-Mg**) imposed by the chelating tmeda ligands and almost identical Ir···M···Ir (125.90(9)° in **2-Mg** vs 127.73(3)° in **2-Mn**) and Ir···M–O (122.68(5)° in **3-Mg** vs 123.69(5)° in **3-Mn**) angles. The M···Ir1 distances in **3-M** tend to be slightly shorter than both the M···Ir1 and M···Ir2 distances in **2-M**. The M···Ir2 contacts in **2-M**, in turn, are marginally shorter than the M···Ir1 contacts, but the distances are virtually the same in all four structures and lie in a remarkably narrow range between 2.6474(6) Å and 2.695(3) Å. Cotton’s formal shortness ratio (FSR)^76^ is used as a normalization method in order to compare these metal–metal distances independently of the different atomic radii of Mg and Mn. The FSR is defined as the ratio of the crystallographically observed distance between two metal centers to the sum of their metallic radii as defined by Pauling.^75^ Due to the difference in atomic radii between Mg and Mn, the nearly identical metal–metal distances lead to distinct FSRs of 1.01 to 1.03 in **2-Mg** and **3-Mg**, on the one hand, and 1.09 to 1.11 in **2-Mn** and **3-Mn**, on the other hand. Usually only values below unity suggest some covalent interaction between the metal centers.^77^ Therefore, based on the Mg···Ir and Mn···Ir distances observed in the complexes studied here, the presence of metal–metal bonds is not suggested. There are only very few literature precedents of bimetallic complexes associating Mg or Mn with Ir, all of which are structurally unrelated to compounds **2-M** and **3-M** because of entirely different ligand architectures. Yet all examples with Mg···Ir or Mn···Ir contacts feature similar distances between 2.660(2) Å and 2.750(2) Å for Mg^21,78^ and between 2.565(3) Å and 2.694(3) Å for Mn.^79,80^ The distances in **2-M** and **3-M** fall within these ranges.

**Table 1:** Key Structural Parameters (Distances in Å and Angles in Deg) for Complexes **2-M** and **3-M** (M = Mg, Mn)

	2-Mg	2-Mn	3-Mg	3-Mn
M···Ir1	2.695(3)	2.6873(8)	2.6474(6)	2.6489(3)
M···Ir2	2.664(2)	2.6785(7)	–	–
Ir1···Cp*	1.872(8)	1.877(5)	1.873(2)	1.874(2)
Ir2···Cp*	1.863(8)	1.862(5)	–	–
M–N1	2.256(7)	2.337(5)	2.257(1)	2.276(2)
M–N2	2.268(8)	2.324(4)	2.208(2)	2.315(2)
M–O	–	–	1.884(1)	1.959(2)
Ir1···M···Ir2	125.90(9)	127.73(3)	–	–
M···Ir1···Cp*	129.58(9)	129.00(3)	128.89(8)	127.80(6)
M···Ir2···Cp*	175.59(9)	174.76(3)	–	–
N1–M–N2	81.43(3)	79.6(2)	82.11(6)	80.10(6)
Ir1···M–O	–	–	122.68(5)	123.69(5)
M–O–Si	–	–	150.44(8)	149.0(1)

**Figure 2:**
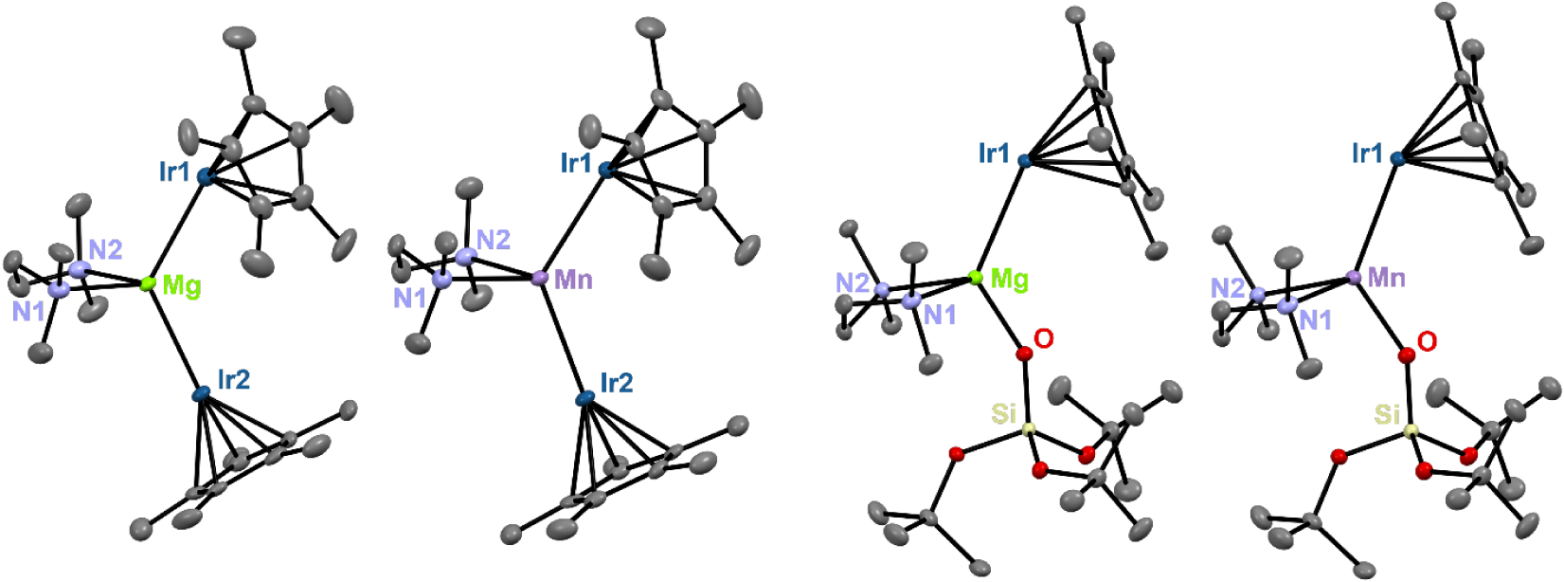
Solid-state molecular structures of **2-M** (left) and **3-M** (right), M = Mg, Mn. Thermal ellipsoids (ORTEP) are drawn at the 50% probability level. Carbon atoms (gray) are unlabeled, and hydrogen atoms are omitted for clarity. Selected bond distances and angles are listed in Table 1.

Interestingly, despite very similar M···Ir1 and M···Ir2 distances, the two iridium centers in **2-Mg** and **2-Mn** have different coordination geometries attributed to changes in the hydride coordination modes. The M···Ir2···Cp* angles (175.59(9)° in **2-Mg** and 174.76(3)° in **2-Mn**, where Cp* denotes the centroid of the carbon atoms defined by the five-membered ring of the cyclopentadienyl ligand) are close to 180°, suggesting that all hydride ligands bridge the M and Ir2 centers in a symmetrical way around the metal–metal axis. In contrast, the M···Ir1···Cp* angles significantly deviate from 180° (129.58(9)° in **2-Mg** and 129.00(3)° in **2-Mn**). This deflection of the Cp* centroid away from the metal–metal axis has been observed in other examples of metal–iridium complexes synthesized from the same [Cp*IrH_4_] precursor, with M···Ir···Cp* angles close to 180° if all hydride ligands are bridging (Scheme 4, right)^30,40^ and decreased M···Ir···Cp* angles roughly between 140° and 160° if part of the hydride ligands are terminal, i.e., located at the Ir centers without any interaction with the second metal (Scheme 4, left).^22,23,29,33,34^ The M···Ir1···Cp* angles in **2-Mg** and **2-Mn** are even smaller and therefore strongly suggest at least one terminal hydride ligand *per* Ir1 center, as drawn in Scheme 3.

The room temperature ^1^H NMR spectrum of the diamagnetic compound **2-Mg** does not permit a definite conclusion concerning the ratio of hydride ligands pertaining to the bridging or terminal coordination modes because all hydride ligands are chemically equivalent at 295 K, pointing to a dynamic exchange between the ligands, which is fast at the NMR time scale. Even at lower temperatures (−30 °C in toluene-*d*_8_), no decoalescence of the hydride resonance can be observed. However, the characteristic singlet resonance at −18.71 ppm integrates for six protons (Figure S6) and thus confirms the total number of hydride ligands represented in Scheme 3. In contrast, none of the broad resonances in the ^1^H NMR spectrum of the paramagnetic compound **2-Mn** (Figure S10) could be assigned to a particular chemical environment. However, the comparison of its infrared spectrum with that of **2-Mg** (Figure 3 top) reveals striking similarities between the two compounds, in particular as regards the strong bands between 1800 and 2200 cm^–1^, the typical region for M–H stretching frequencies in transition-metal hydride complexes.^41^ The combined information from the crystal structures and from the NMR and DRIFT spectra therefore strongly suggests perfectly isostructural compounds **2-Mg** and **2-Mn**, whose only distinguishing feature is the presence of different metal centers (magnesium vs manganese).

**Figure 3:**
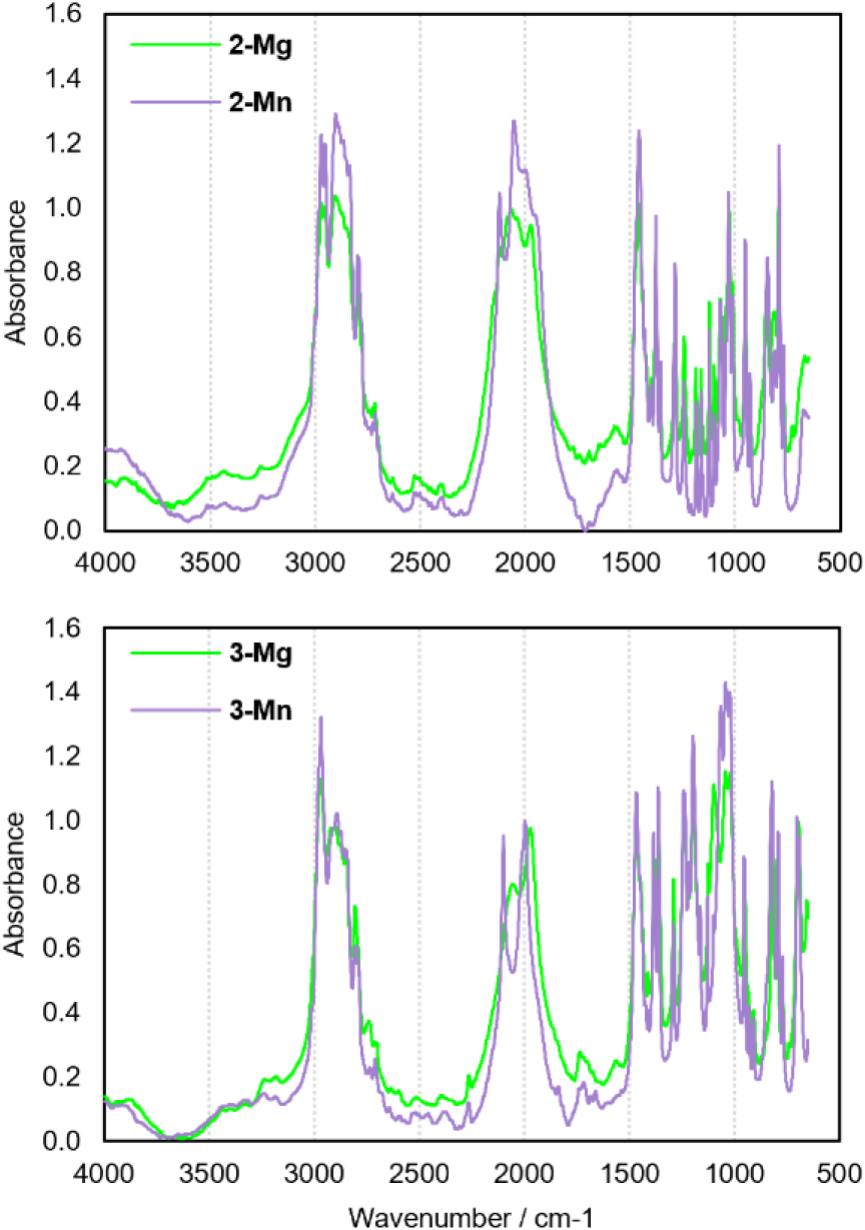
Comparison of DRIFT spectra for compounds **2-M** (top) and **3-M** (bottom) (M = Mg, Mn).

Similar conclusions can be drawn with respect to compounds **3-Mg** and **3-Mn**. In both structures, it is the [IrCp*] fragment containing the Ir1 center with a Cp* centroid “bent away” from the M···Ir axis (M···Ir1···Cp* angles 128.89(8)° in **3-Mg** and 127.80(6)° in **3-Mn**), which is retained after protonolysis by the silanol and elimination of [Cp*IrH_4_] from the “linear” [M­(μ-H)_3_IrCp*] fragment. Accordingly, the ^1^H NMR spectrum of **3-Mg** shows a characteristic hydride resonance at −19.27 ppm, integrating for three protons. As discussed for **2-Mg**, these protons are chemically equivalent under the conditions of the solution-state NMR experiment at 295 K, whereas the crystal structure is indicative of a terminal hydride ligand at the Ir center. Once more, a comparison of the DRIFT spectra of **3-Mg** and **3-Mn** (Figure 3 bottom) proves the similarity of these two compounds and implies that the same holds true for the hydride ligands of **3-Mn** despite a deficient NMR characterization due to its paramagnetism.

Differences in the behavior of magnesium and manganese dialkyls are noted if the iridium tetrahydride precursor directly reacts with the nonchelated precursors, the polymeric 1,4-dioxane adduct of dineopentylmagnesium^9^ and the tetrameric dineopentylmanganese,^12^ instead of **1-Mg** and **1-Mn** (Scheme 5). [Mn­(CH_2_ *t*Bu)_2_]_4_ reacts with [Cp*IrH_4_] to give compound **5-Mn**, a heterometallic cluster comprising manganese and iridium centers in a stoichiometric 4:3 ratio. Its crystal structure (Figure 4) illustrates that the tetrameric structure of the precursor is preserved in the sense that four manganese centers remain in relatively close spatial proximity to each other (shortest Mn···Mn contacts: 2.617(1) and 2.866(1) Å, corresponding to FSR of 1.12 and 1.23, respectively) and are coordinated by bridging Cp* iridium hydride fragments, giving rise to Mn···Ir distances between 2.734(1) Å (FSR 1.13) and 3.086(1) Å (FSR 1.27), significantly longer than those in **2-Mn** and **3-Mn**, and almost equilateral “Mn_2_Ir triangles” with angles close to 60° (Table 2). Only half of the neopentyl ligands from the dialkylmanganese tetramer have been eliminated through protonolysis by the iridium precursor, leaving behind three terminal neopentyl ligands (CH_2_ *t*Bu) and one neopentyl ligand bridging Mn2 and Mn3 (μ-CH_2_ *t*Bu).

**Table 2:** Key Structural Parameters for Complex **5-Mn**

*d*/Å		∠/deg (**°**)	
Mn1···Mn2	2.866(1)	Ir1···Mn1···Ir3	113.80(4)
Mn2···Mn3	2.617(1)	Ir1···Mn1···Mn2	58.53(3)
Mn1···Ir1	2.771(1)	Ir2···Mn2···Ir3	100.24(3)
Mn1···Ir3	2.7645(9)	Ir2···Mn2···Mn3	65.04(3)
Mn2···Ir2	2.998(1)	Ir2···Mn3···Ir3	91.96(3)
Mn2···Ir3	2.734(1)	Ir2···Mn4···Ir3	106.32(4)
Mn3···Ir2	3.035(1)	Ir3···Mn1···Mn2	58.07(3)
Mn3···Ir3	3.086(1)	Mn1···Ir3···Mn2	62.83(3)
Mn4···Ir2	2.737(1)	Mn1···Ir3···Mn3	112.75(3)
Mn4···Ir3	2.763(1)	Mn1···Mn2···Mn3	125.45(5)
Ir···Cp* (avg.)	1.876(1)	Mn2···Ir2···Mn3	51.40(3)
Mn1,3,4–CH_2_ *t*Bu (avg.)	2.13(1)	Mn2···Ir3···Mn3	53.01(3)
Mn2,3–(μ-CH*t*Bu) (avg.)	2.391(9)	Mn2···Mn3···Ir3	56.59(3)
		Mn1,3,4–CH_2_–*t*Bu (avg.)	121.7(6)
		Mn2–(μ-CH)–Mn3	66.3(2)

**Scheme 5:**
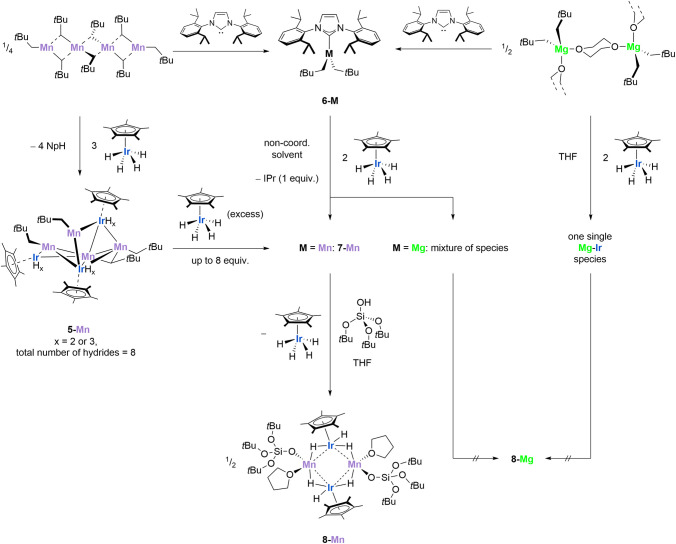
Syntheses of Bimetallic Manganese–Iridium Compounds through Protonolysis between an Iridium Polyhydride and Different Manganese Dialkyl Complexes

**Figure 4:**
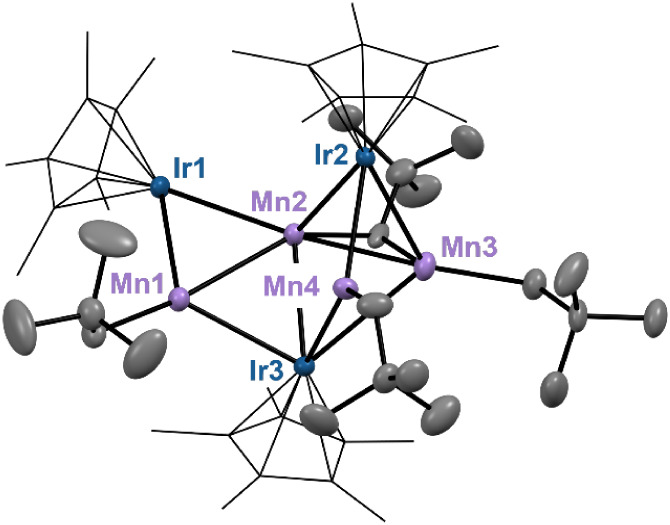
Solid-state molecular structure of **5-Mn** with thermal ellipsoids (ORTEP) drawn at the 50% probability level. Carbon atoms (gray) are unlabeled, Cp* ligands are wireframed, and hydrogen atoms are omitted for clarity. Selected metal–metal distances and angles are listed in Table 2.

Interestingly, even though four of the alkyl ligands from [Mn­(CH_2_ *t*Bu)_2_]_4_ have been eliminated, only three [Cp*IrH_*x*_] fragments appear in the structure of **5-Mn**. This suggests a double protonolysis by one of the Ir tetrahydrides, leading to one [Cp*IrH_2_] and two [Cp*IrH_3_] fragments in the final product, **5-Mn**. Note that such double deprotonation of Cp*IrH_4_ has been observed before in related Al–Ir, Ta–Ir, Nb–Ir, and Mo–Ir compounds.^23,29,33,35,81^ The IR spectrum confirms the presence of hydride ligands with M–H stretching frequencies similar to those discussed earlier (Figure 3, Figure S43), even though their number and coordination modes are not evident from the ^1^H NMR spectrum due to paramagnetic chemical shifts and line broadening. Any further characterization is hampered by the fact that **5-Mn**, stable as a crystalline solid as evidenced by a correct elemental analysis result, rapidly evolves into other paramagnetic species under concomitant formation of neopentane as soon as it is dissolved in deuterated benzene (see NMR spectra in Figures S18 and S19). It can also be modified by adding an excess of [Cp*IrH_4_], which brings about a color change from dark-brown to yellow. A total of up to 8 equiv of the Ir complex *per* [Mn­(CH_2_ *t*Bu)_2_]_4_ is consumed until the formation of a stable compound, **7-Mn** (Scheme 5, Figure S25). Supposedly containing Mn and Ir in a 1:2 ratio, **7-Mn** could not be obtained in single-crystal quality and is paramagnetic, making it impossible to propose a structure for this compound. Nonetheless, this result emphasizes that **5-Mn** is a structural “snapshot” in a series of protonolysis reactions toward more stable compounds (and eventually **7-Mn)**, and that **5-Mn** is isolable only thanks to its fast crystallization from *n*-pentane immediately after its formation (see Experimental Section for details).

In order to prevent uncontrolled protonolysis of [Mn­(CH_2_ *t*Bu)_2_]_4_ by [Cp*IrH_4_] and the formation of polynuclear compounds, the tetrameric dialkylmanganese was transformed into the *N*-heterocyclic carbene-coordinated monomer **6-Mn** by the addition of 1,3-bis­(2,6-diisopropylphenyl)­imidazol-2-ylidene (IPr). The crystal structure of **6-Mn** is represented in Figure 5 (right) and contains a manganese center coordinated by the NHC and two neopentyl ligands in a nearly regular trigonal planar coordination geometry with C–Mn–C angles close to 120° and summing up to 360°. A detailed structural description is reported by Mulvey and Robertson^82^ as well as by Tonzetich^83^ and coworkers for the closely related complex [(IPr)­Mn­(CH_2_SiMe_3_)_2_] bearing two neosilyl instead of neopentyl ligands. The analogous compound **6-Mg** could also be synthesized and was characterized by ^1^H and ^13^C NMR, infrared spectroscopy, and elemental analysis (Figures S20–S23, S39). Its crystal structure (Figure 5, left) presents a strong disorder repeatedly occurring despite multiple crystallization attempts under different conditions (see p. S25 in SI and Table S5). **6-Mn** reacts with up to two equivalents of [Cp*IrH_4_] under evolution of neopentane to yield a yellow solution resembling that of compound **7-Mn** obtained from a direct reaction of the dialkyl tetramer with the Ir precursor. Indeed, the ^1^H NMR spectrum (Figure S26) reveals not only the elimination of neopentane but also the formation of the uncoordinated NHC ligand and the same characteristic broad resonances of paramagnetic **7-Mn**. This result is consistent with a more labile NHC coordination to Mn­(II) than to other transition-metal ions described in the literature, for example, Fe­(II) or Co­(II),^83–85^ and explains the carbene dissociation from **6-Mn** following the alkane elimination and the coordination of the residual [Cp*IrH_3_] fragments to the Mn center. The high steric demand of the IPr ligand, originally intended to generate isolable heterobimetallic complexes of low nuclearity, in fact, does not prevent the formation of **7-Mn**.

**Figure 5:**
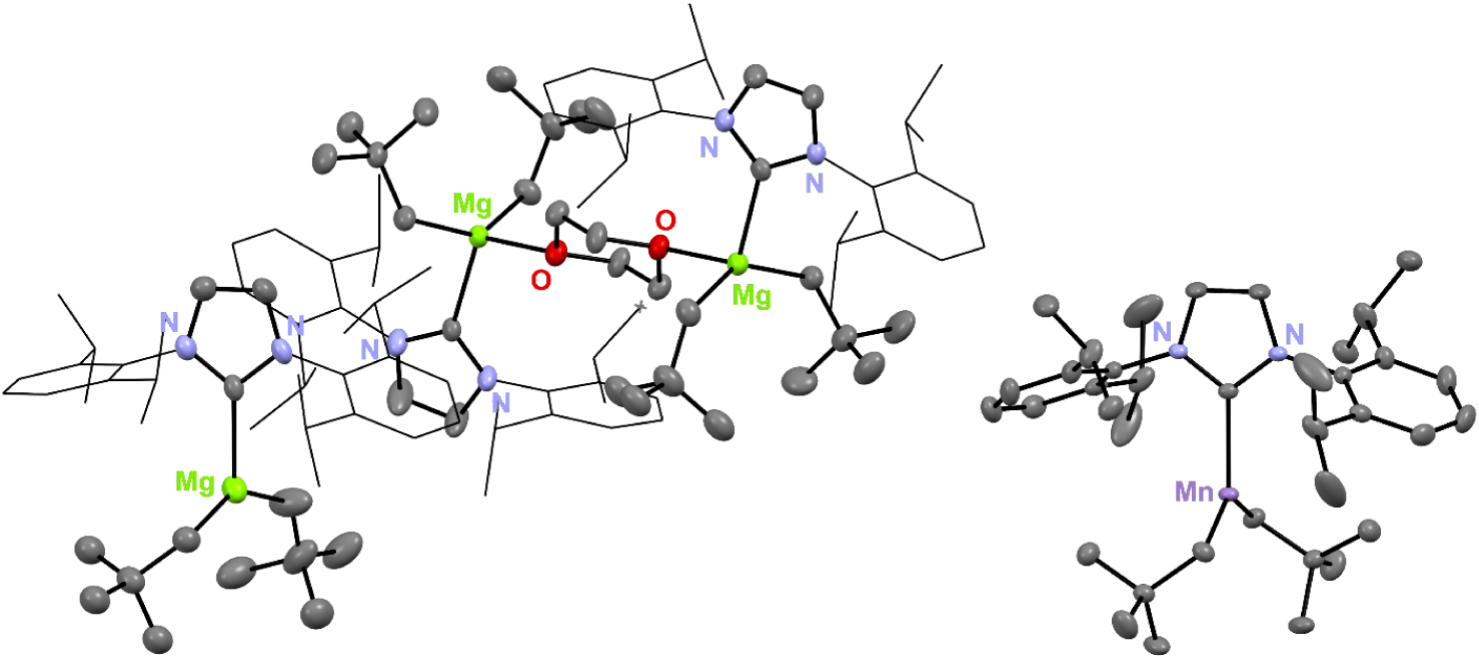
Solid-state molecular structures of **6-Mg** (left) and **6-Mn** (right) with thermal ellipsoids (ORTEP) drawn at the 50% probability level. Carbon atoms (gray) are unlabeled, and hydrogen atoms are omitted for clarity. Selected bond distances (Å) and angles (°): Mg–NHC_(avg)_ 2.314(3), Mn–NHC 2.228(2), Mg–CH_2_ *t*Bu_(avg)_ 2.160(4), Mn–CH_2_ *t*Bu 2.147(2); Mg–C–*t*Bu_(avg)_ 127.2(3), Mn–C–*t*Bu 116.5(1), C–Mg–C_(avg)_ 140.5(2), C–Mn–C 131.4(1), C–Mg–NHC_(avg)_ 105.2(1), C–Mn–NHC 114.31(5).

Although **7-Mn** could not be characterized by X-ray diffraction, its reaction with tris­(*tert*-butoxy)­silanol in the presence of a coordinating solvent such as tetrahydrofuran (THF) leads to the elimination of one equivalent of [Cp*IrH_4_] and the formation of a new paramagnetic compound, **8-Mn** (Figure S28), which could be isolated in single-crystal quality (77% yield based on Mn). According to its crystal structure (Figure 6), **8-Mn** is a tetranuclear “diamond-core” Mn_2_Ir_2_ complex with a relatively long Mn···Mn contact of 3.0669(4) Å (FSR 1.31) and two bridging [Cp*IrH_3_] fragments at distances of 2.7551(4) to 2.8055(4) Å (FSR 1.13 to 1.16) to the Mn centers. Besides, Mn1 and Mn2 are each coordinated by one siloxy ligand and one THF molecule in a distorted tetrahedral coordination environment characterized by an O–M–O angle of 98.45(5)° and an Ir1···Mn···Ir2 angle of 113.06(2)°. As highlighted in the discussion of **2-M** and **3-M**, two different types of Mn···Ir···Cp* angles are found depending on the localization and coordination mode of the hydride ligands in the structure: the Mn1···Ir2···Cp* angle (168.58(9)°) is close to 180°, and the Cp* centroid is almost located on the prolonged Mn1···Ir2 axis, whereas the Mn1···Ir1···Cp* angle (123.32(9)°) is significantly sharper, and the Cp* centroid deflected away from the prolonged Mn1···Ir1 axis. These features suggest that part of the hydride ligands bridge the Mn and Ir centers while the remaining terminal hydride ligands are localized at the Ir centers (Scheme 5). Their presence in the molecule is confirmed by the DRIFT spectrum of **8-Mn** (M–H stretching bands between 1800 and 2100 cm^–1^, see Figure S46), even though a more detailed characterization is again impeded by its paramagnetism, leading to uncharacteristic broad resonances around 5 ppm and between 15 and 35 ppm in the ^1^H NMR spectrum (Figure S29).

**Figure 6:**
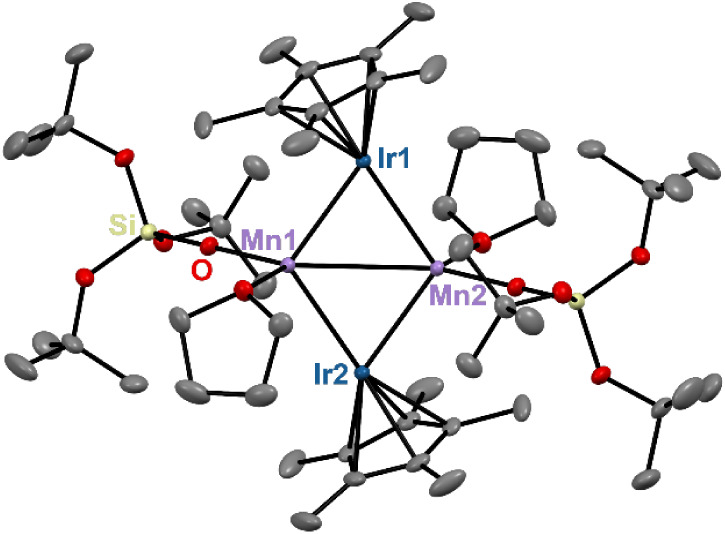
Solid-state molecular structure of **8-Mn** with thermal ellipsoids (ORTEP) drawn at the 50% probability level. Carbon atoms (gray) are unlabeled, and hydrogen atoms are omitted for clarity. Selected bond distances (Å) and angles (°): Mn1···Mn2 3.0669(4), Mn1···Ir1 2.7551(4), Mn1···Ir2 2.8055(4), Mn–O­(Si) 1.932(1), Mn–O­(thf) 2.224(1), Ir···Cp* 1.866(1); Mn1···Ir···Mn2 66.94(1), Ir1···Mn···Ir2 113.06(1), Mn1···Ir1···Cp* 123.32(9), Mn1···Ir2···Cp* 168.58(9), Mn–O–Si 174.61(9), O–Mn–O 98.45(5).

Unfortunately, the magnesium–iridium compounds analogous to **5-Mn** and **8-Mn** could not be obtained for different reasons. In a noncoordinating solvent (C_6_D_6_), [Mg­(CH_2_ *t*Bu)_2_(1,4-dioxane)]_*n*_ neither dissolves nor reacts, even in the presence of an excess amount of [Cp*IrH_4_]. In a coordinating solvent (THF-*d*_8_), the reaction between the dioxane adduct and up to 2 equiv of [Cp*IrH_4_] *per* Mg proceeds slowly over several hours and under gradual formation of a single hydride species with a putative Mg–Ir stoichiometric ratio of 1:2, exhibiting characteristic ^1^H NMR resonances at 2.14 ppm (Cp* methyl groups) and −19.22 ppm (hydride ligands), visibly without passing through any intermediate products of the kind of **5-Mn** (Figure S30). The hypothetical [Mg_4_Ir_3_] complex **5-Mg** seems to be inaccessible because of the composition of the corresponding dialkylmetal precursor [Mg­(CH_2_ *t*Bu)_2_(1,4-dioxane)]_*n*_, synthesized through precipitation of the corresponding Grignard reagent with 1,4-dioxane and subsequent sublimation.^86^ The residual dioxane molecules prevent the formation of base-free dialkylmagnesium oligomers, like in the case of [Mn­(CH_2_ *t*Bu)_2_]_4_, which is directly obtained from the reaction of neopentylmagnesium chloride with manganese­(II) chloride in noncoordinating solvents.^7^ However, the above discussion underlines that the tetrameric structure of the manganese precursor plays an essential role in the formation of **5-Mn** through partial protonolysis by [Cp*IrH_4_].

The alternative route via the NHC-coordinated dialkyl **6-Mg** (Figure 5, Figures S20–S23 and S44) is feasible in a noncoordinating solvent. However, the reaction of **6-Mg** with two equivalents of [Cp*IrH_4_] in C_6_D_6_ is fast and yields several hydride species, as illustrated by five different new ^1^H NMR resonances between −15 and −20 ppm (Figure S31). This reaction outcome is different from the direct reaction of [Mg­(CH_2_ *t*Bu)_2_(1,4-dioxane)]_*n*_ with the Ir precursor in THF-*d*_8_, underlining once more that for reactions starting from precursors other than the isostructural compounds **1-Mg** and **1-Mn** (Scheme 3), the reactivity and speciation in the magnesium and manganese systems are dependent on the structure of the respective dineopentyl precursor to enable a perfect correspondence of the results and crystal structures obtained for Mn. Accordingly, the hypothetical compound **8-Mg** could not be isolated through either of the described pathways.

## Conclusions

Several examples of isostructural heterobimetallic hydride complexes combining magnesium and manganese with iridium were prepared via alkane elimination reactions between a variety of magnesium and manganese dialkyl precursors and an iridium tetrahydride complex. The nuclearity and M:Ir stoichiometry can be adjusted by the addition of simple chelating ligands and proton-donating reagents at different stages of the synthetic procedure. Their great structural similarities are illustrated by pairs of crystal structures whose space groups, unit cell parameters, interatomic distances, and angles are nearly unaffected by the choice of either magnesium or manganese. This approach helps overcome two ubiquitous obstacles in the characterization of hydride complexes in general and of those of the first-row transition metals in particular. First, the difficulty of localizing hydride ligands in the immediate vicinity of heavy metal centers based on X-ray diffraction data could be resolved by complementary DRIFT and NMR spectra as well as a detailed discussion of indirect information from key structural parameters (e.g., M···Ir···Cp* angles) confirming the presence (DRIFT), number (NMR), and probable positions/coordination modes (XRD) of the hydride ligands in the diamagnetic magnesium–iridium complexes. Second, the difficulty of interpreting the NMR spectra of paramagnetic compounds featuring extremely broad resonances across wide chemical shift ranges, such as the reported manganese–iridium complexes, could be surmounted once their structural properties had been shown to be identical to those of the well-understood magnesium–iridium analogs through XRD and DRIFT studies, a fact which allows for detailed conclusions concerning their structures despite a sincere lack of information from NMR data.

However, further reactivity investigations disclosed the importance of the isostructural precursors **1-Mg** and **1-Mn** in the synthesis of analogous heterobimetallic Mg–Ir and Mn–Ir complexes. The use of the unchelated or NHC-coordinated Mg and Mn dialkyls led to different results depending in part on the precursor structure and in part on other necessary experimental modifications related, for example, to the choice of solvent. While different bimetallic polynuclear Mn–Ir species could be isolated and characterized by controlling the stoichiometric ratio of the Mn and Ir precursors, these results did not translate to the Mg–Ir system. These examples illustrate the inherent challenges of this chemistry and emphasize that such parallels are not universally observed. Nonetheless, the present study underlines the interest and relevance of the similarity between Mg­(II) and Mn­(II) regarding their coordination chemistry and extends the current knowledge about the structural analogy of Mg and Mn coordination compounds to the area of heterobimetallic complexes containing a (second) transition metal. The results obtained for the bimetallic Mg–Ir and Mn–Ir species might stimulate future work on metals other than rare and expensive iridium, in particular on first-row transition metals. In this context, developing a general synthetic strategy using diamagnetic Mg compounds as NMR spectroscopic handles to gain a deeper understanding of structurally similar but paramagnetic Mn compounds might represent an interesting and rewarding subject of research.

## Experimental Section

### General Considerations

Unless otherwise stated, all reactions were carried out under an argon atmosphere using a standard dual manifold Schlenk line setup. Materials and reactants sensitive to air and moisture were manipulated in an MBraun glovebox operating at H_2_O and O_2_ levels < 0.1 ppm. All glassware was dried at 100 °C for at least 24 h prior to use. Tetrahydrofuran, toluene, and *n*-pentane were purified by passage through a column of activated alumina, dried in the presence of sodium and benzophenone, vacuum-transferred to a storage flask, and freeze–pump–thaw degassed. 1,4-dioxane and deuterated solvents (THF-*d*_8_, C_6_D_6_) were dried over sodium and benzophenone, vacuum-transferred to a storage flask, and freeze–pump–thaw degassed. N,N,N′,*N*′-Tetramethylethylenediamine (TMEDA, purchased as anhydrous reagent > 99.5%, purified by redistillation) and fluorobenzene were transferred to storage flasks under inert atmosphere, freeze–pump–thaw degassed and stored over 4 Å molecular sieves. [Mg­(CH_2_ *t*Bu)_2_(1,4-dioxane)]_*n*_,^8,86^ [Mn­(CH_2_ *t*Bu)_2_]_4_,^7,12^ [Cp*IrH_4_] (Cp* = C_5_Me_5_),^87,88^ and 1,3-bis­(2,6-diisopropylphenyl)­imidazol-2-ylidene (IPr)^89^ were prepared according to literature procedures. All other reagents were purchased from commercial sources and used without any further purification.

### Characterization Methods

#### Infrared Spectroscopy

Solid samples were diluted in dry potassium bromide (KBr) inside a glovebox, sealed under argon in a DRIFTS cell equipped with KBr windows, and analyzed on a Nicolet 670 FT-IR spectrometer.

#### Elemental Analysis

Elemental analyses were performed under an inert atmosphere at Mikroanalytisches Labor Pascher, Germany.

#### X-ray Structural Determination

Experimental details regarding single-crystal X-ray diffraction measurements are provided in the SI. CCDC 2544746–2544755, 2549630, 2553738, and 2553896 contain the supplementary crystallographic data for this publication. These data are provided free of charge by the Cambridge Crystallographic Data Center.

#### NMR Spectroscopy

Solution NMR spectra were recorded on a Bruker AV-300 spectrometer (^1^H and ^13^C spectra) and on a Jeol ECZ 400 spectrometer (^13^C DEPT-135 and ^1^H–^13^C HSQC spectra). ^1^H and ^13^C chemical shifts are reported relative to an external tetramethylsilane standard at 0.00 ppm and were manually referenced to the residual solvent signal.

### Synthetic Procedures

#### Synthesis of [Mg­(tmeda)­(CH_2_ *t*Bu)_2_] (**1-Mg**)

TMEDA (193 μL, 1.30 mmol, 1.1 equiv) is added to a suspension of [Mg­(CH_2_ *t*Bu)_2_(1,4-dioxane)]_*n*_ (300 mg, 1.18 mmol, 1.0 equiv) in 4.0 mL of *n*-pentane at 20 °C. The reaction mixture turns into a slightly opaque, colorless solution within seconds. After 15 min at ambient temperature, the solution is filtered through a pad of glass wool in a Pasteur pipette. The filtrate is cooled to −40 °C overnight to afford the product as pale rose crystals, which are isolated by filtration and dried under vacuum (250 mg, 75%). ^1^H NMR (C_6_D_6_, 300 MHz, 295 K): δ/ppm = 1.80 (s, 12 H, NC*H*_3_), 1.52 (superposed s, 22 H, N­(C*H*_2_)_2_N and C­(C*H*_3_)_3_), −0.05 (s, 4 H, MgC*H*_2_); ^13^C­{^1^H} NMR (C_6_D_6_, 75 MHz, 295 K): δ/ppm = 56.34 (2 C, N­(*C*H_2_)_2_N), 46.89 (4 C, N*C*H_3_), 37.92 (6 C, C­(*C*H_3_)_3_), 33.37 (2 C, Mg*C*H_2_), 32.13 (2 C, *C*(CH_3_)_3_). Elemental analysis (%). Calculated for **1-Mg** (MgC_16_H_38_N_2_): C 67.95, H 13.54, N 9.91; found: C 67.93, H 13.55, N 9.97.

#### Synthesis of [Mn­(tmeda)­(CH_2_ *t*Bu)_2_] (**1-Mn**)

TMEDA (225 μL, 1.71 mmol, 4.5 equiv) is added to a reddish-brown solution of [Mn­(CH_2_ *t*Bu)_2_]_4_ (300 mg, 0.38 mmol, 1.0 equiv) in 4.0 mL of *n*-pentane at 20 °C. The solution turns colorless within seconds. After 15 min at ambient temperature, the solution is cooled to −40 °C overnight to afford the product as pale rose crystals, which are isolated by filtration and dried under vacuum (390 mg, 82%). ^1^H NMR (C_6_D_6_, 300 MHz, 295 K): δ/ppm = 20.17 (ν_1/2_ ≈ 1800 Hz), 2.57. Elemental analysis (%). Calculated for **1-Mn** (MnC_16_H_38_N_2_): C 61.31, H 12.22, N 8.94; found: C 61.44, H 12.22, N 8.92.

#### Synthesis of [Mg­(tmeda)­{(μ-H)_3_(IrCp*)}­{(μ-H)_2_(HIrCp*)}] (**2-Mg**)

A colorless solution of **1-Mg** (42 mg, 0.15 mmol, 1.0 equiv) in 2.0 mL *n*-pentane is cooled to −40 °C. A colorless solution of [Cp*IrH_4_] (100 mg, 0.30 mmol, 2.0 equiv) in 1.5 mL *n*-pentane prepared at 20 °C is added dropwise under stirring. The reaction mixture is left to stand at ambient temperature for 30 min and is subsequently stored at −40 °C overnight to afford the product as colorless crystals, which are isolated by filtration and dried under vacuum (102 mg, 85%). ^1^H NMR (C_6_D_6_, 300 MHz, 295 K): δ/ppm = 2.33 (s, 30 H, Cp*), 2.14 (s, 12 H, NC*H*_3_), 1.73 (s, 4 H, N­(C*H*_2_)_2_N), −18.71 (s, 6 H, μ-H); ^13^C­{^1^H} NMR (C_6_D_6_, 75 MHz, 295 K): δ/ppm = 88.33 (10 C, Cp* rings), 55.79 (2 C, N­(*C*H_2_)_2_N), 49.60 (4 C, N*C*H_3_), 12.10 (10 C, Cp* methyl groups). Elemental analysis (%). Calculated for **2-Mg** (MgIr_2_C_26_H_52_N_2_): C 38.96, H 6.54, N 3.50; found: C 39.30, H 6.55, N 3.41.

#### Synthesis of [Mn­(tmeda)­{(μ-H)_3_(IrCp*)}­{(μ-H)_2_(HIrCp*)}] (**2-Mn**)

A colorless solution of **1-Mn** (47 mg, 0.15 mmol, 1.0 equiv) in 2.0 mL *n*-pentane is cooled to −40 °C. A colorless solution of [Cp*IrH_4_] (100 mg, 0.30 mmol, 2.0 equiv) in 1.5 mL *n*-pentane prepared at 20 °C is added dropwise under stirring. The solution first turns yellow and then light orange within minutes. The reaction mixture is left to stand at ambient temperature for 30 min and is subsequently stored at −40 °C overnight to afford the product as orange crystals, which are isolated by filtration and dried under vacuum (86 mg, 69%). ^1^H NMR (C_6_D_6_, 300 MHz, 295 K): δ/ppm = 32.88, 26.90, 21.90, 2.40, 2.17. Elemental analysis (%). Calculated for **2-Mn** (MnIr_2_C_26_H_52_N_2_): C 37.53, H 6.30, N 3.37; found: C 37.17, H 6.28, N 3.21.

#### Synthesis of [Mg­{OSi­(O*t*Bu)_3_}­(tmeda)­(μ-H)_2_(HIrCp*)] (**3-Mg**)

Tris­(*tert*-butoxy)­silanol (33 mg, 0.12 mmol, 1.0 equiv) is added as a solid to a colorless solution of **2-Mg** (100 mg, 0.12 mmol, 1.0 equiv) in 1.5 mL *n*-pentane at 20 °C. The resulting solution is colorless and is left to stand for 1 h at ambient temperature. Storage at −40 °C overnight affords the product as colorless, block-shaped crystals, which are isolated by filtration and dried under vacuum (66 mg, 72%). ^1^H NMR (C_6_D_6_, 300 MHz, 296 K): δ/ppm = 2.31 (superposed s, 21 H, Cp* and 2 NC*H*_3_), 2.03 (s, 6 H, 2 NC*H*_3_), 1.99 (m, 2 H, 1 NC*H*_2_), 1.72 (m, 2 H, 1 N*CH*_2_), 1.60 (s, 27 H, OC­(C*H*_3_)_3_), −19.27 (s, 3 H, μ-H); ^13^C­{^1^H} NMR (C_6_D_6_, 75 MHz, 295 K): δ/ppm = 88.56 (5 C, Cp* ring), 70.58 (3 C, O*C*(CH_3_)_3_), 55.88 (2 C, N­(*C*H_2_)_2_N), 48.42 (2 C, N*C*H_3_), 47.01 (2 C, N*C*H_3_), 32.33 (9 C, OC­(*C*H_3_)_3_), 12.42 (5 C, Cp* methyl groups). Elemental analysis (%). Calculated for **3-Mg** (MgIrC_28_H_61_N_2_O_4_Si): C 45.79, H 8.37, N 3.81; found: C 46.16, H 8.45, N 3.95.

#### Synthesis of [Mn­{OSi­(O*t*Bu)_3_}­(tmeda)­(μ-H)_2_(HIrCp*)] (**3-Mn**)

Tris­(*tert*-butoxy)­silanol (32 mg, 0.12 mmol, 1.0 equiv) is added as a solid to a light orange solution of **2-Mn** (100 mg, 0.12 mmol, 1.0 equiv) in 1.5 mL *n*-pentane at 20 °C. The resulting solution has a yellow color and is left to stand for 1 h at ambient temperature. Storage at −40 °C overnight affords the product as light yellow, plate-shaped crystals, which are isolated by filtration and dried under vacuum (77 mg, 84%). ^1^H NMR (C_6_D_6_, 300 MHz, 296 K): δ/ppm = 32.79, 24.60 (ν_1/2_ ≈ 450 Hz), 2.32 (ν_1/2_ ≈ 600 Hz). Elemental analysis (%). Calculated for **3-Mn** (MnIrC_28_H_61_N_2_O_4_Si): C 43.96, H 8.04, N 3.66; found: C 44.27, H 8.13, N 3.66.

#### Synthesis of [Cp*Ir­(μ-H)_3_IrCp*]­[B­(C_6_F_5_)_4_] (**4**)

A solution of **2-Mg** or **2-Mn** (0.054 mmol, 1.0 equiv) in 3.0 mL *n*-pentane is added to a suspension of trityl tetrakis­(pentafluorophenyl)­borate (50 mg, 0.054 mmol, 1.0 equiv) in 5.0 mL *n*-pentane at 20 °C. The reaction mixture is stirred for 24 h to afford a suspension of a pale yellow solid. The solvent is removed under vacuum, and the residue is dissolved in 1.5 mL fluorobenzene and layered with 5.0 mL *n*-pentane in a scintillation vial. After standing at 20 °C, the vial is stored at −40 °C for 3 days to give the product as orange crystals, which are isolated by filtration and dried under vacuum (18 mg [from **2-Mg**] and 21 mg [from **2-Mn**], 45% and 53% based on Ir).

#### Synthesis of [{Mn­(CH_2_ *t*Bu)}_4_(H)_9_{IrCp*}_3_] (**5-Mn**)

A reddish-brown solution of [Mn­(CH_2_ *t*Bu)_2_]_4_ (79 mg, 0.10 mmol, 1.0 equiv) in 2.5 mL *n*-pentane is cooled to −40 °C. A colorless solution of [Cp*IrH_4_] (100 mg, 0.30 mmol, 3.0 equiv) in 1.5 mL *n*-pentane prepared at 20 °C is added dropwise under stirring. The color quickly turns from reddish-brown to dark-brown. Once the addition is complete, the reaction mixture is left to stand at ambient temperature for 15 min, during which small, dark-brown, needle-shaped crystals begin to form. It is then stored at −40 °C overnight to increase the yield and to avoid decomposition or evolution of the complex into other species. After filtration and drying under vacuum, the product is obtained as a dark-brown, crystalline solid (95 mg, 64%). ^1^H NMR (C_6_D_6_, 300 MHz, 296 K): δ/ppm = 61.88, 42.73, 25.33, 23.10, 22.11, 21.23, 19.28, 16.91, 14.41, 12.94, 10.95, 9.25, 6.56, 6.04. Elemental analysis (%). Calculated for **5-Mn** (Mn_4_Ir_3_C_50_H_98_): C 40.15, H 6.60; found: C 39.55, H 6.36.

#### Synthesis of [Mg­(IPr)­(CH_2_ *t*Bu)_2_] (**6-Mg**)

A colorless solution of IPr (153 mg, 0.39 mmol, 1.0 equiv) in 3 mL toluene is added to a colorless suspension of [Mg­(CH_2_ *t*Bu)_2_(1,4-dioxane)]_*n*_ (100 mg, 0.39 mmol, 1.0 equiv) in 3 mL toluene at 20 °C. The resulting reaction mixture is a slightly opaque, colorless solution. Solvents are removed under vacuum, and the remaining solid is thoroughly dried. The residue is dissolved in 8 mL *n*-pentane, and the solution is filtered through a pad of glass wool. The filtrate is stored at −40 °C overnight to afford the product as colorless crystals, which are isolated by filtration and dried under vacuum (106 mg, 49%). **6-Mg** cocrystallizes with 1,4-dioxane (^1^/_3_ equiv based on Mg, see Figure 5 and Table S5). ^1^H NMR (C_6_D_6_, 300 MHz, 295 K): δ/ppm = 7.21–7.26 (m, 2 H, aromatic *p*–C*H*), 7.10–7.13 (m, 4 H, aromatic *m*–C*H*), 6.44 (s, 2 H, NHC–C*H*), 3.33 (s, ^8^/_3_ H, 1,4-dioxane), 2.76 (hept, *J* = 6.8 Hz, 4 H, *i*Pr–C*H*), 1.39 (d, *J* = 6.9 Hz, 12 H, *i*Pr–C*H*_3_), 1.21 (s, 18 H, *t*Bu–C*H*_3_), 1.00 (d, *J* = 6.9 Hz, 12 H, *i*Pr–C*H*_3_), −0.32 (s, 4 H, C*H*_2_ *t*Bu); ^13^C­{^1^H} NMR (C_6_D_6_, 75 MHz, 295 K): δ/ppm = 145.51 (4 C, aromatic *o*-*Ci*Pr), 135.89 (2 C, aromatic *ipso*-*C*), 130.43 (4 C, aromatic *p*-*C*H), 124.55 (4 C, aromatic *m*-*C*H), 123.21 (2 C, N–*C*H), 67.11 (^4^/_3_ C, 1,4-dioxane), 37.13 (6 C, *t*Bu-*C*H_3_), 32.94 (2 C, *t*Bu-*C*_*q*_), 32.15 (2 C, Mg*C*H_2_), 28.74 (4 C, *i*Pr-*C*H), 25.42 (4 C, *i*Pr-*C*H_3_), 23.57 (4 C, *i*Pr-*C*H_3_), carbene C not detected. Elemental analysis (%). Calculated for [**6-Mg** + 2 **6-Mg** • C_4_H_8_O_2_] (Mg_3_C_115_H_182_N_6_O_2_): C 78.76, H 10.46, N 4.79; found: C 78.91, H 10.53, N 4.81.

#### Synthesis of [Mn­(IPr)­(CH_2_ *t*Bu)_2_] (**6-Mn**)

A colorless solution of IPr (395 mg, 1.01 mmol, 4.0 equiv) in 5 mL toluene is added to a reddish-brown solution of [Mn­(CH_2_tBu)_2_]_4_ (200 mg, 0.25 mmol, 1.0 equiv) in 5 mL *n*-pentane at 20 °C. The resulting solution immediately turns bright yellow. Solvents are removed under vacuum, and the remaining solid is thoroughly dried. The residue is dissolved in 15 mL *n*-pentane, and the solution is filtered through a pad of glass wool. The filtrate is stored at −40 °C overnight to afford the product as big yellow crystals, which are isolated by filtration and dried under vacuum (505 mg, 85%). ^1^H NMR (C_6_D_6_, 300 MHz, 295 K): δ/ppm = 25.88 (ν_1/2_ ≈ 2200 Hz), 8.52, 4.35. Elemental analysis (%). Calculated for **6-Mn** (MnC_37_H_58_N_2_): C 75.86, H 9.98, N 4.78; found: C 75.82, H 10.01, N 4.82.

#### Synthesis of **7-Mn**

Compound **7-Mn** can be obtained *in situ* in two different ways: (1) The synthesis from [Mn­(CH_2_ *t*Bu)_2_]_4_ (30 mg, 0.04 mmol) follows the procedure described for compound **5-Mn**, except that eight equivalents of [Cp*IrH_4_] (100 mg, 0.30 mmol) *per* dialkylmanganese tetramer must be added instead of three equivalents. The color first changes from reddish-brown to dark-brown, indicating intermediate formation of **5-Mn**, and then gradually becomes lighter and finally turns yellow. (2) Alternatively, **7-Mn** is formed if compound **6-Mn** (88 mg, 0.15 mmol, 1.0 equiv) is treated with [Cp*IrH_4_] (100 mg, 0.30 mmol, 2.0 equiv) in a solution of *n*-pentane. However, the free *N*-heterocyclic ligand IPr can only be partly removed from the reaction mixture (colorless crystals after cooling to −40 °C for several hours), leaving behind a characteristic yellow solution of **7-Mn**. If used in the subsequent synthesis of **8-Mn**, **7-Mn** should therefore be prepared according to method (1) to avoid contamination by residual IPr.

#### Synthesis of [Mn­{OSi­(O*t*Bu)_3_}­(thf)­(μ-H)_2_(HIrCp*)]_2_ (**8-Mn**)

A yellow solution of compound **7-Mn**, prepared from 30 mg (0.04 mmol) [Mn­(CH_2_ *t*Bu)_2_]_4_ and 100 mg (0.30 mmol) [Cp*IrH_4_] according to method (1), in 2.0 mL *n*-pentane is cooled to −40 °C. A solution of tris­(*tert*-butoxy)­silanol (40 mg, 0.15 mmol, 1.0 equiv based on manganese) in 0.5 mL *n*-pentane prepared at 20 °C is added dropwise under stirring or gentle shaking. The reaction mixture is left to stand at ambient temperature for 1 h, during which the yellow color becomes less intense. Three drops of tetrahydrofuran are added using a Pasteur pipette, and the resulting solution is stored at −40 °C overnight to afford the product as yellowish, almost colorless crystals, which are isolated by filtration and dried under vacuum (85 mg, 77% based on manganese). ^1^H NMR (C_6_D_6_, 300 MHz, 296 K): δ/ppm = 33.65, 32.91, 28.17, 21.40, 5.24. Elemental analysis (%). Calculated for **8-Mn** (Mn_2_Ir_2_C_52_H_106_O_10_Si_2_): C 43.32, H 7.41; found: C 43.09, H 7.47.

## Supplementary Material



## Notes

NMR, IR, X-ray crystallography data (PDF)
