# Supplementary material for: Manganese(II) and Magnesium(II) Analogies in Iridium Hydride Coordination Chemistry
Source: Inorg Chem. 2026 Jun 12;65(25):14274–86. doi: 10.1021/acs.inorgchem.6c01844 (PMC13321303; doi:10.1021/acs.inorgchem.6c01844)
Supplement: Supplementary file 1 [file ic6c01844_si_001.pdf]

Supporting information

# Manganese(II) and Magnesium(II) Analogies in Iridium Hydride Coordination Chemistry

Till Neumann[a], Erwann Jeanneau[b], Chloé Thieuleux[a], and Clément Camp[a]*

[a] Laboratory of Catalysis, Polymerization, Processes and Materials (CP2M, UMR 5128), CNRS, Universite Claude Bernard Lyon 1, CPE Lyon, Institut de Chimie de Lyon, 3 rue Victor Grignard, 69616 Villeurbanne, France

[b] Centre de Diffractométrie Henri Longchambon, Universite Claude Bernard Lyon 1, 5 rue de la Doua, 69100 Villeurbanne, France

clement.camp@univ-lyon1.fr

## Contents

Additional experimental procedures ........ 2

NMR spectroscopy data ........ 3

Diffuse reflectance infrared Fourier-transform (DRIFT) spectroscopy data ........ 24

Crystallographic data........ 30

References ........ 38

## Additional experimental procedures

Synthesis of [Mg(tmeda)(OSi{O*t*Bu}$_3$)$_2$].

A colorless solution of tris(*tert*-butoxy)silanol (20 mg, 0.076 mmol, 1.0 eq.) in 0.5 mL *n*-pentane is added to a colorless solution of **2-Mg** (30 mg, 0.038 mmol, 0.5 eq.) or **3-Mg** (56 mg, 0.076 mmol, 1.0 eq.) in 1.5 mL *n*-pentane at 20 °C. The reaction mixture is left at this temperature for 15 minutes and then cooled to −40 °C overnight to afford the product as colorless, block-shaped crystals along with colorless, long needles of [Cp*IrH$_4$], which also crystallizes in these conditions. Alternatively, a solution of tris(*tert*-butoxy)silanol (281 mg, 1.06 mmol, 1.0 eq.) in 1.5 mL *n*-pentane is added to a solution of **1-Mg** (150 mg, 0.53 mmol, 0.5 eq.) in 2.5 mL *n*-pentane at 20 °C, leading to vigorous bubbling of the reaction mixture due to neopentane formation. After 15 minutes at this temperature, the reaction mixture is cooled to −40 °C overnight to afford the pure crystalline product.

$^1$H NMR ($C_6D_6$, 300 MHz, 295 K): δ / ppm = 2.24 (s, 12 H, NC$H_3$), 1.89 (s, 4 H, N(C$H_2$)$_2$N), 1.53 (s, 54 H, OC(C$H_3$)$_3$); $^{13}$C{$^1$H} NMR ($C_6D_6$, 75 MHz, 295 K): δ / ppm = 70.61 (6 C, O*C*(CH$_3$)$_3$), 55.90 (2 C, N(*C*H$_2$)$_2$N), 46.54 (4 C, N*C*H$_3$), 32.25 (18 C, OC(*C*H$_3$)$_3$).

Synthesis of [Mn(tmeda)(OSi{O*t*Bu}$_3$)$_2$].

A colorless solution of tris(*tert*-butoxy)silanol (20 mg, 0.076 mmol, 1.0 eq.) in 0.5 mL *n*-pentane is added to a yellowish solution of **2-Mn** (31 mg, 0.038 mmol, 0.5 eq.) or **3-Mn** (58 mg, 0.076 mmol, 1.0 eq.) in 1.5 mL *n*-pentane at 20 °C. The reaction mixture is left at this temperature for 15 minutes and then cooled to −40 °C overnight to afford the product as colorless, block-shaped crystals along with colorless, long needles of [Cp*IrH$_4$], which also crystallizes in these conditions. Alternatively, a solution of tris(*tert*-butoxy)silanol (253 mg, 0.96 mmol, 1.0 eq.) in 1.5 mL *n*-pentane is added to a solution of **1-Mn** (150 mg, 0.48 mmol, 0.5 eq.) in 2.5 mL *n*-pentane at 20 °C, leading to vigorous bubbling of the reaction mixture due to neopentane formation. After 15 minutes at this temperature, the reaction mixture is cooled to −40 °C overnight to afford the pure crystalline product.

$^1$H NMR ($C_6D_6$, 300 MHz, 295 K): δ / ppm = 2.08 ($\nu_{1/2} \approx$ 900 Hz).

NMR spectroscopy data

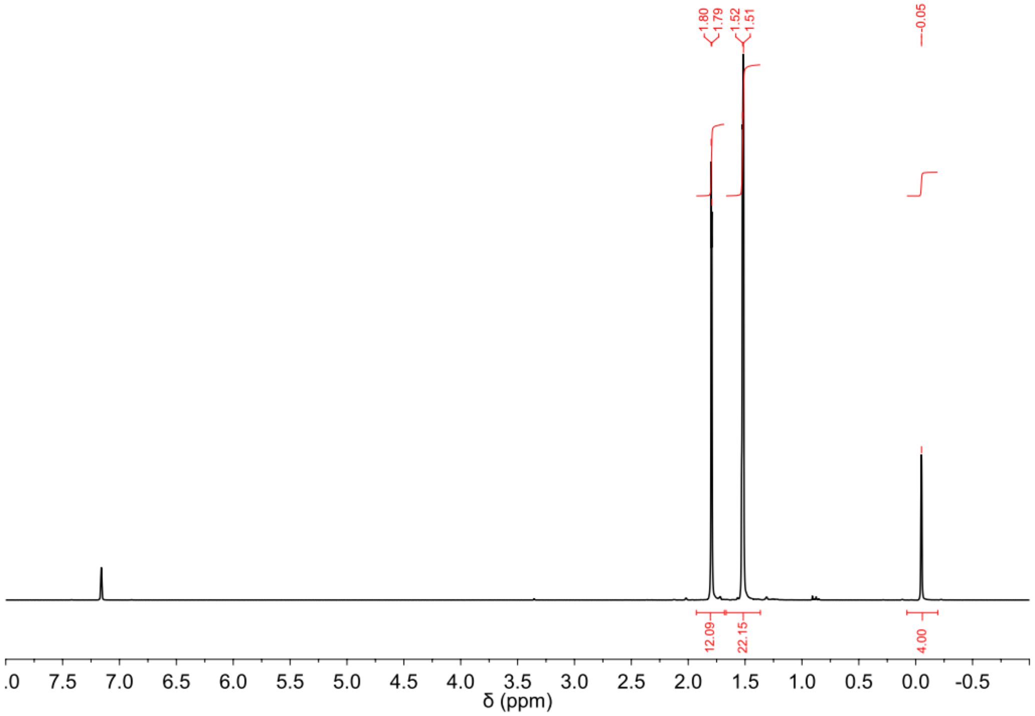


**Figure S1.** $^{1}H$ NMR spectrum (300 MHz, $C_6D_6$, 295 K) for compound **1-Mg**

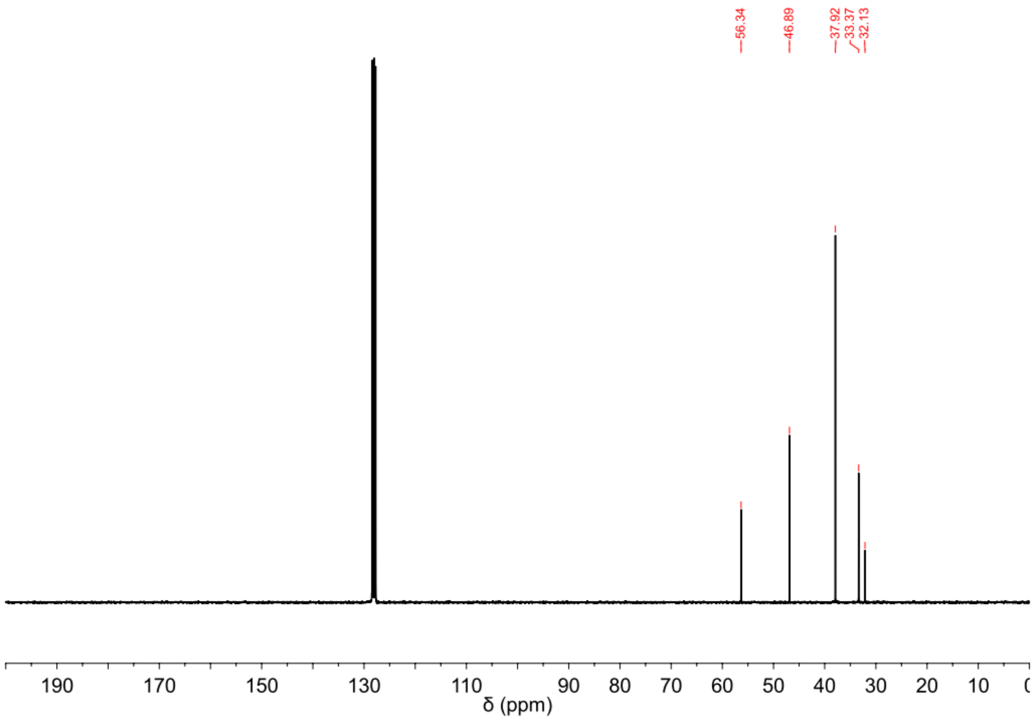


**Figure S2.** $^{13}C\{^{1}H\}$ NMR spectrum (75 MHz, $C_6D_6$, 295 K) for compound **1-Mg**

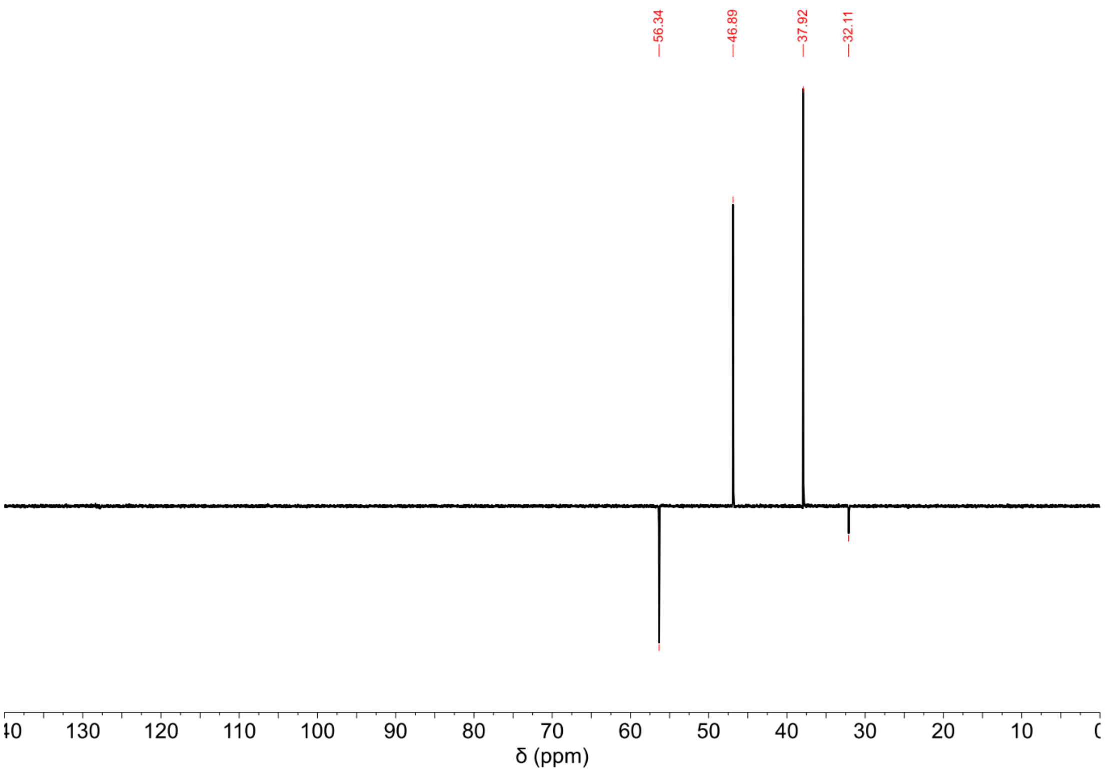


**Figure S3.** $^{13}C\{^{1}H\}$ DEPT-135 NMR spectrum (100 MHz, $C_6D_6$, 293 K) for compound **1-Mg**

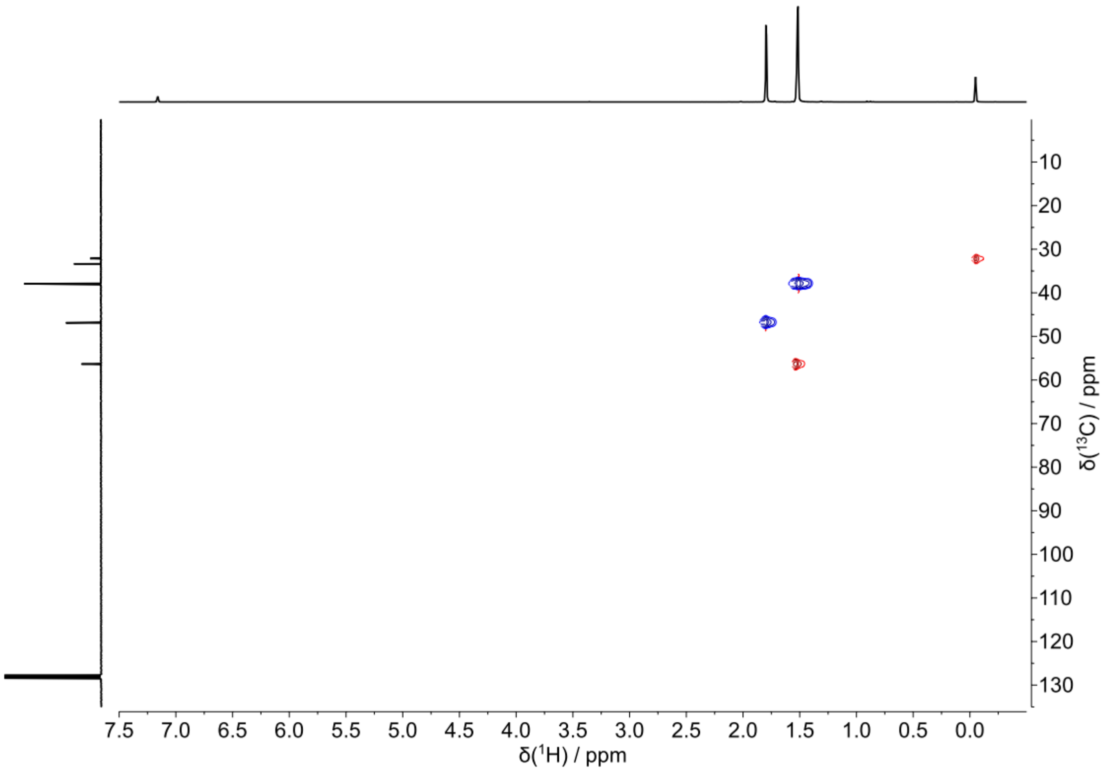


**Figure S4.** $^{1}H$-$^{13}C$ HSQC NMR spectrum (400 MHz, $C_6D_6$, 293 K) for compound **1-Mg**

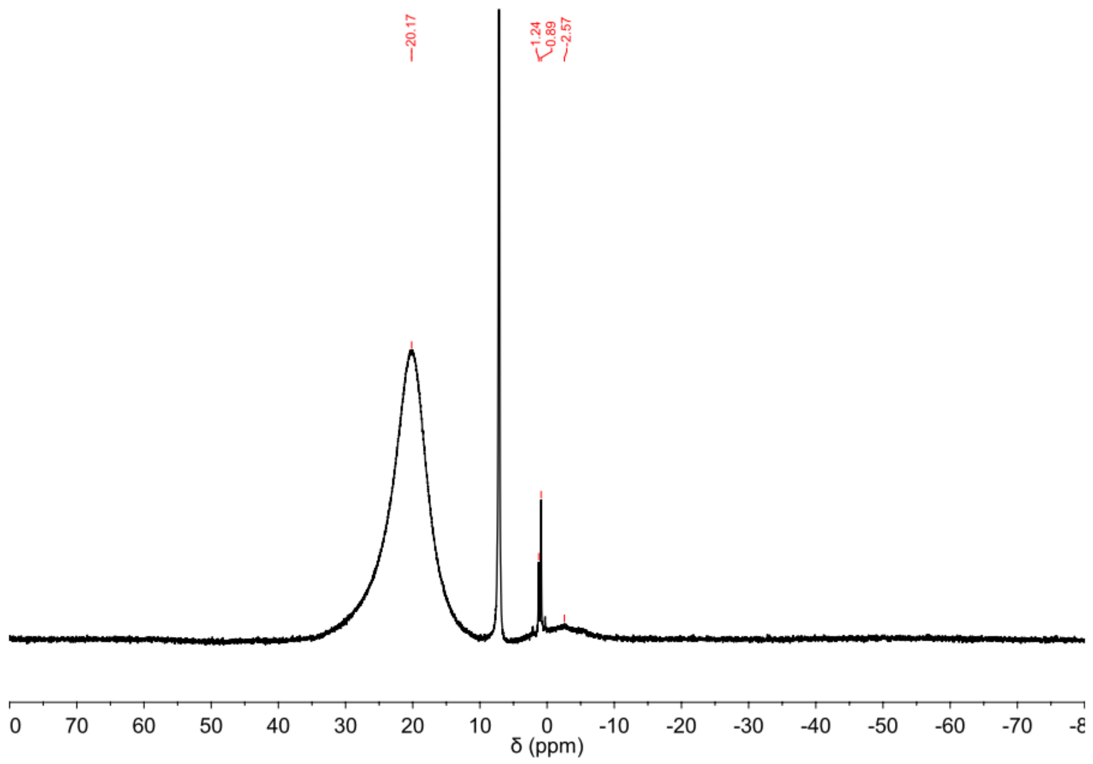


**Figure S5.** $^{1}$H NMR spectrum (300 MHz, $C_6D_6$, 295 K) for compound **1-Mn**. Resonances at 0.89 and 1.24 ppm due to trace amounts of *n*-pentane in the purified product

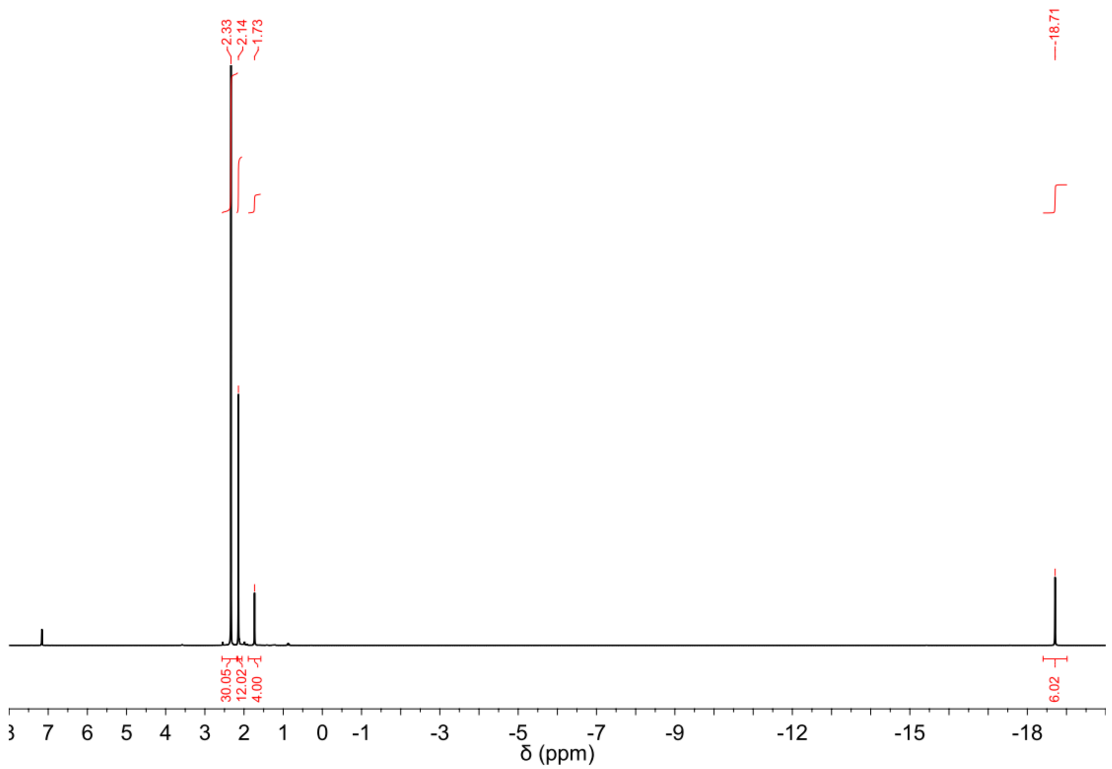


**Figure S6.** $^{1}$H NMR spectrum (300 MHz, $C_6D_6$, 295 K) for compound **2-Mg**

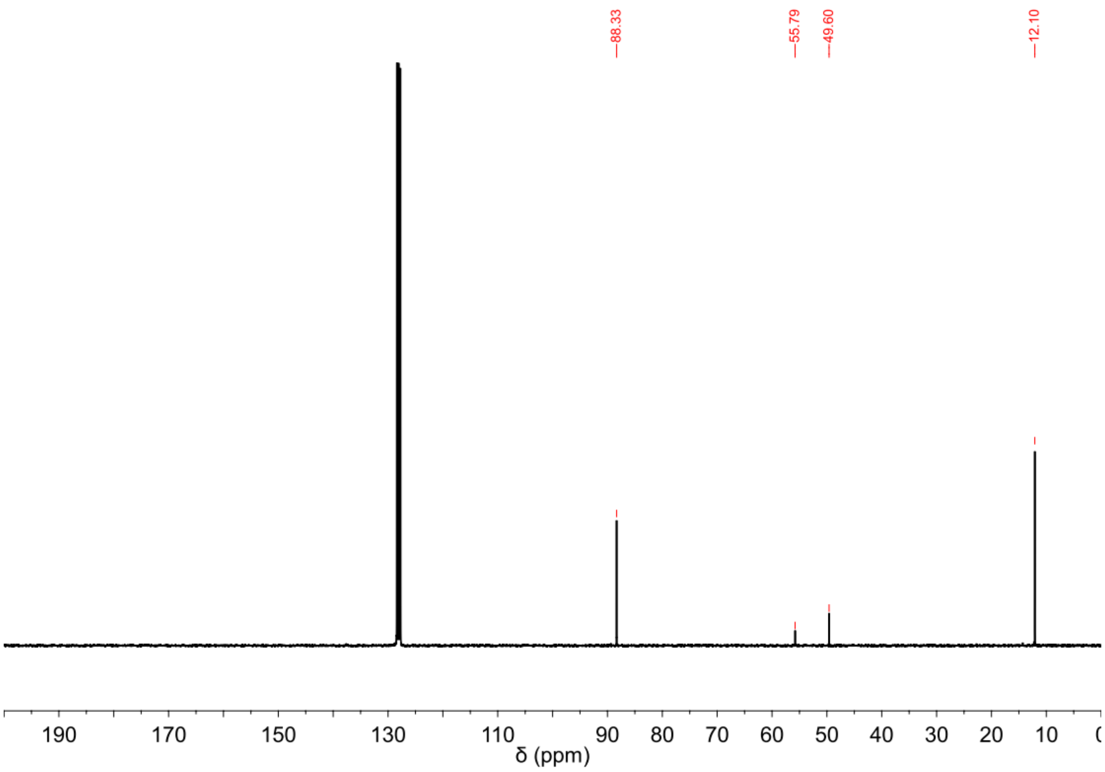


**Figure S7.** $^{13}C\{^{1}H\}$ NMR spectrum (75 MHz, $C_6D_6$, 295 K) for compound **2-Mg**

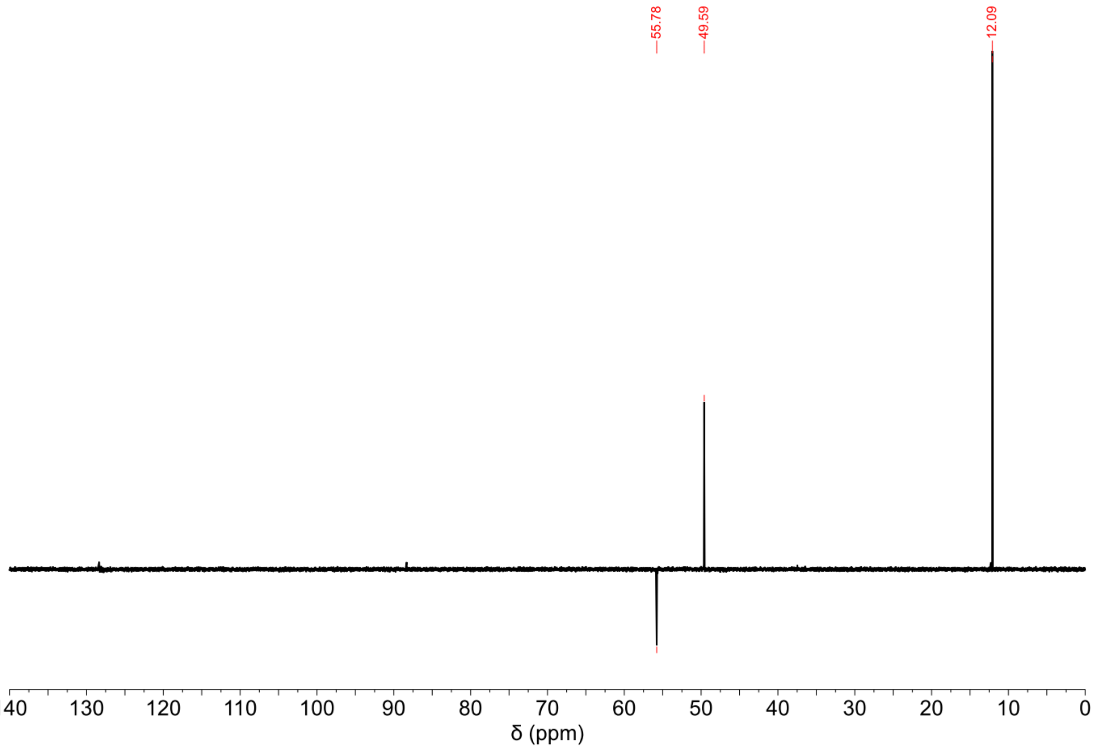


**Figure S8.** $^{13}C\{^{1}H\}$ DEPT-135 NMR spectrum (100 MHz, $C_6D_6$, 293 K) for compound **2-Mg**

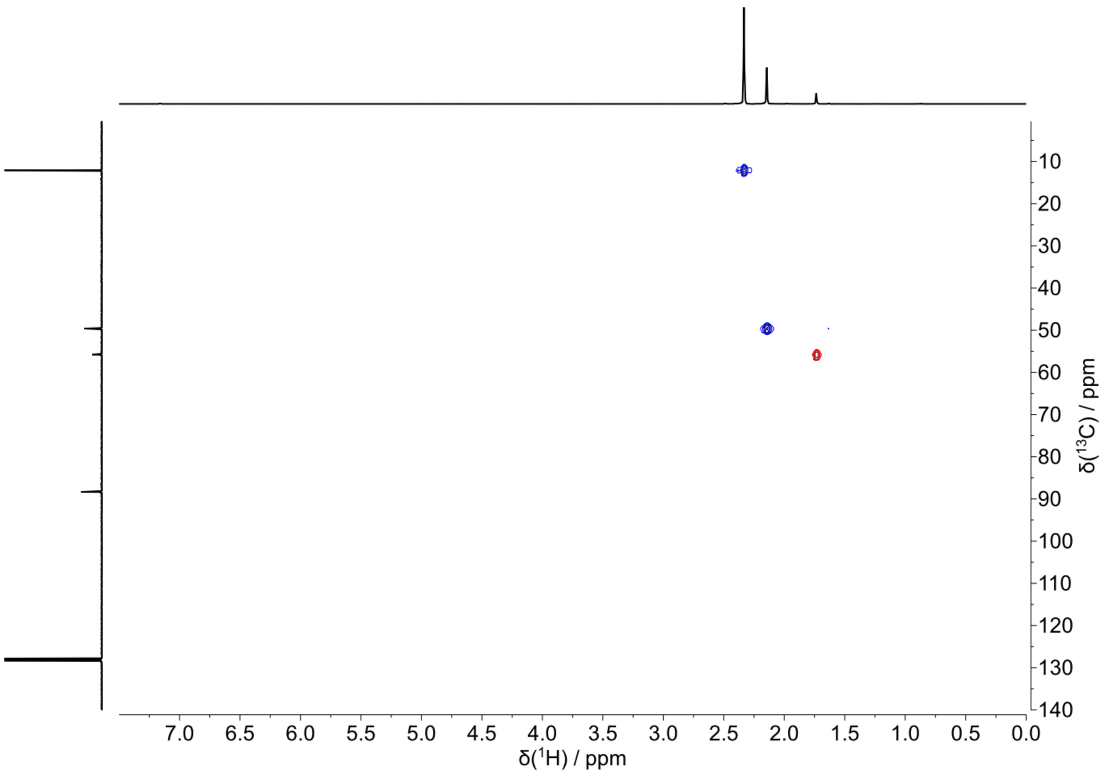


**Figure S9.** $^{1}H$-$^{13}C$ HSQC NMR spectrum (400 MHz, $C_6D_6$, 293 K) for compound **2-Mg**

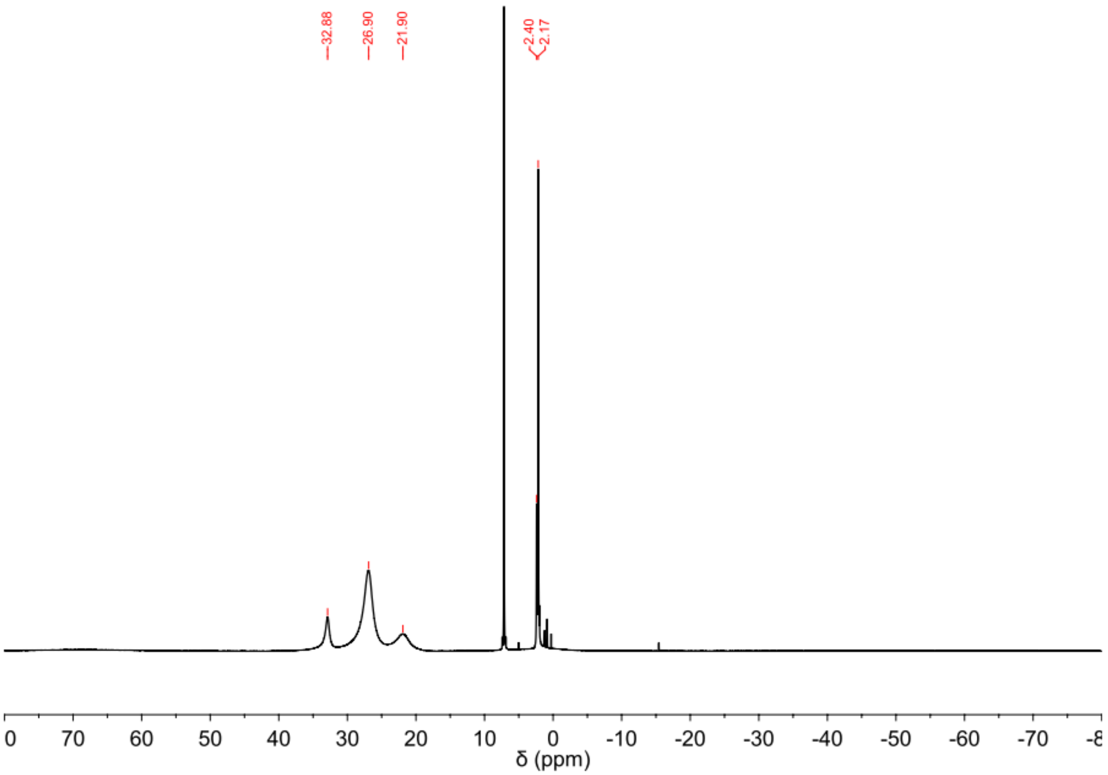


**Figure S10.** $^{1}H$ NMR spectrum (300 MHz, $C_6D_6$, 295 K) for compound **2-Mn**

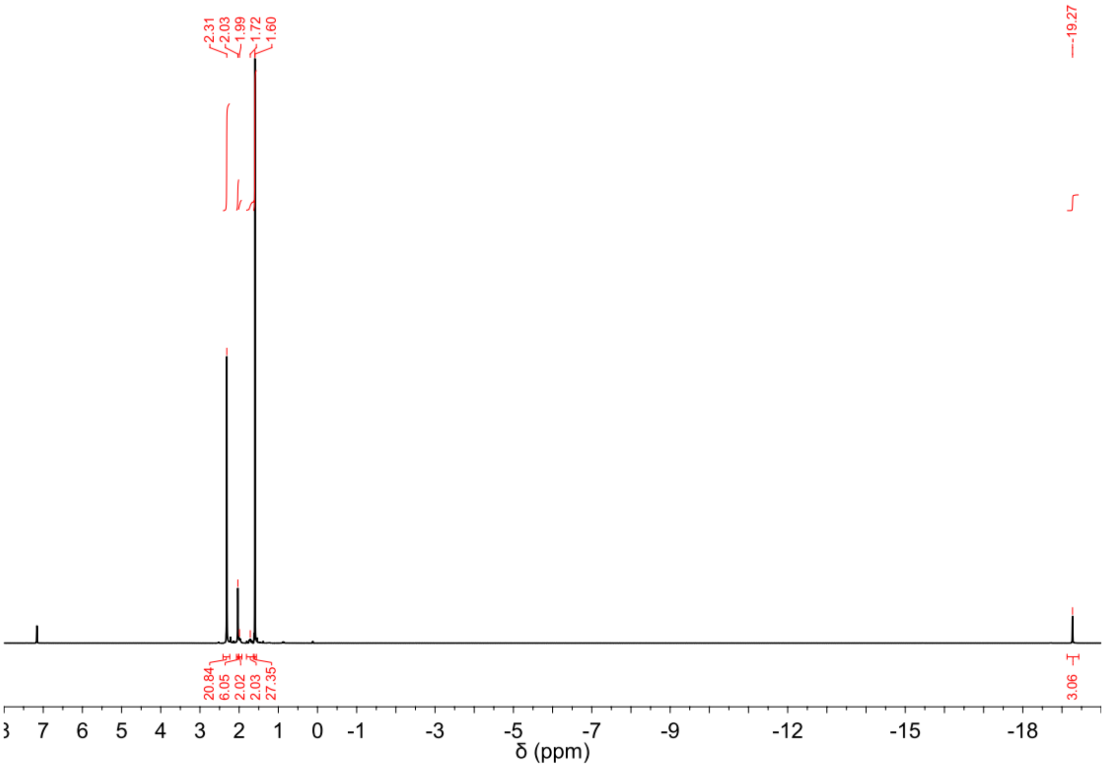


**Figure S11.** $^{1}H$ NMR spectrum (300 MHz, $C_6D_6$, 295 K) for compound **3-Mg**

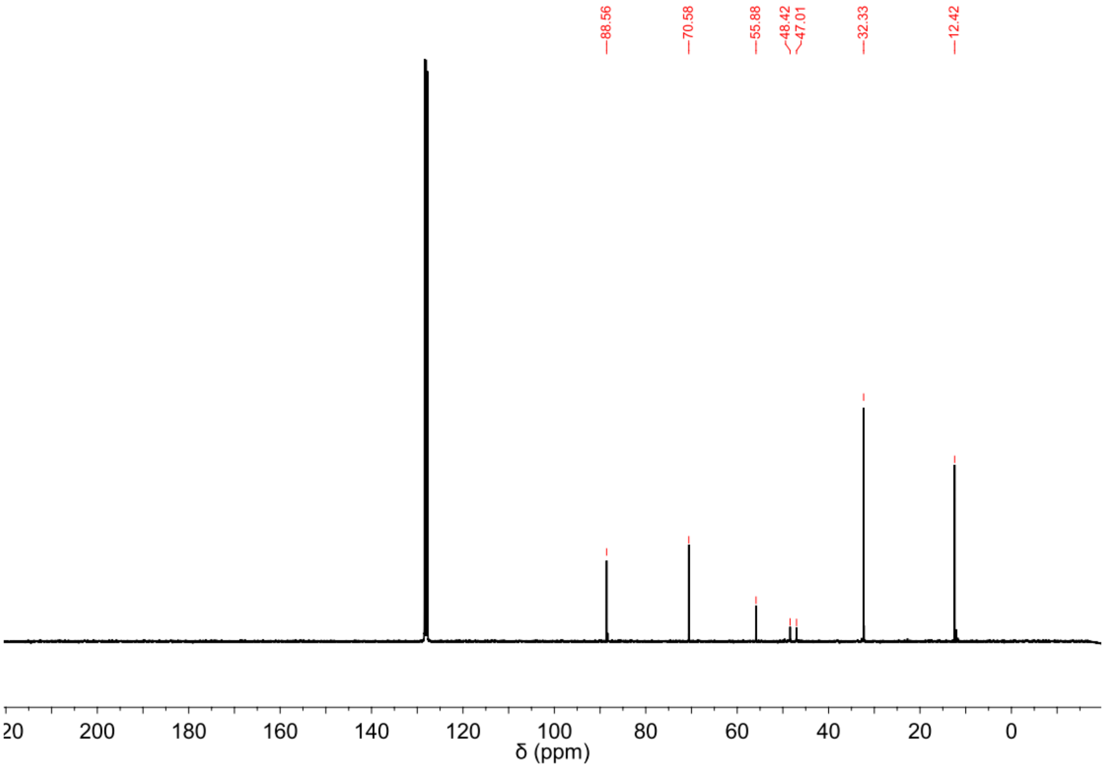


**Figure S12.** $^{13}C\{^{1}H\}$ NMR spectrum (75 MHz, $C_6D_6$, 295 K) for compound **3-Mg**

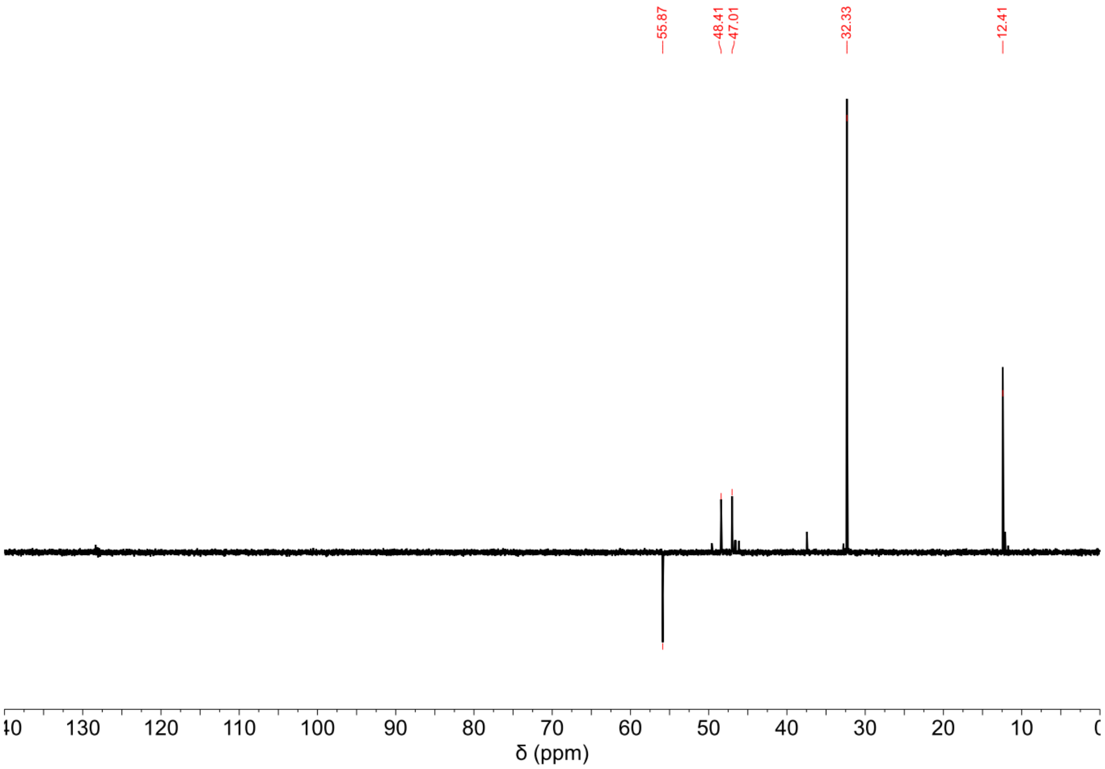


**Figure S13.** $^{13}C\{^{1}H\}$ DEPT-135 NMR spectrum (100 MHz, $C_6D_6$, 293 K) for compound **3-Mg**

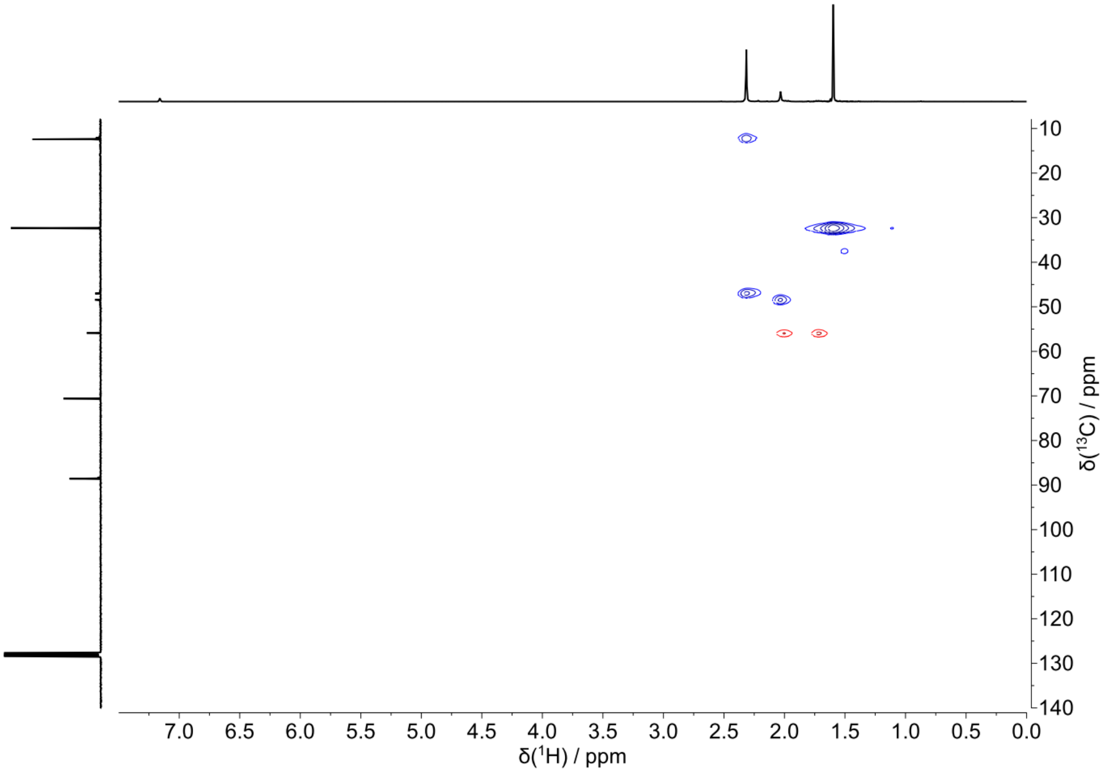


**Figure S14.** $^{1}H$-$^{13}C$ HSQC NMR spectrum (400 MHz, $C_6D_6$, 293 K) for compound **3-Mg**

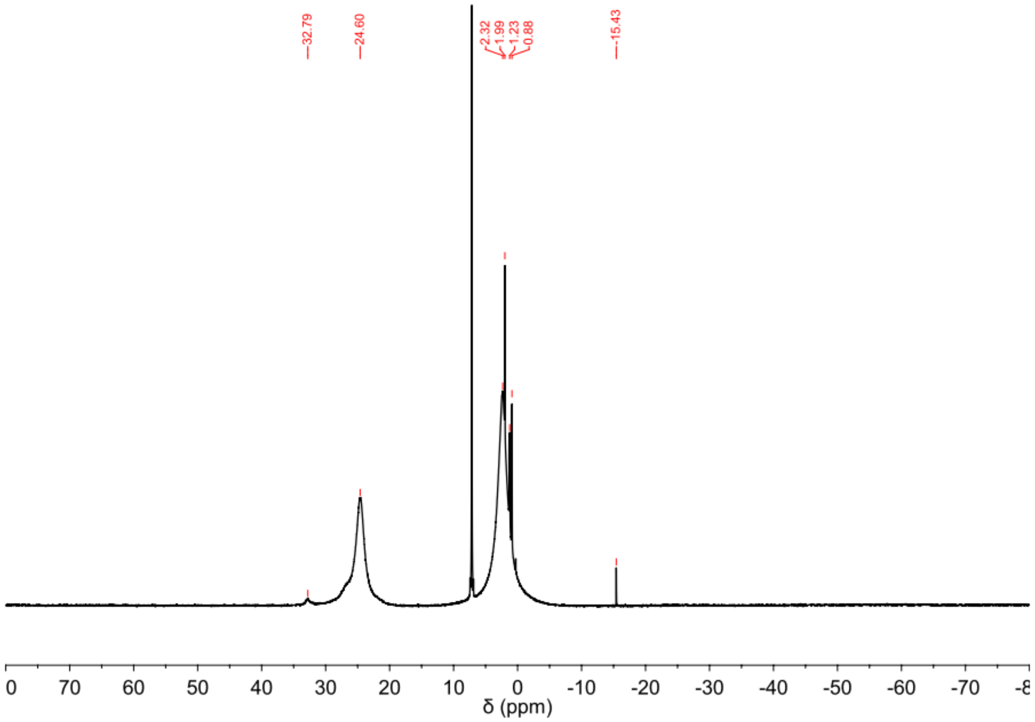


**Figure S15.** $^{1}$H NMR spectrum (300 MHz, $C_6D_6$, 296 K) for compound **3-Mn**. Resonances at 0.88, 1.23 ppm (*n*-pentane) and -15.43, 1.99 ppm ([Cp*$IrH_4$]) due to trace impurities in the purified product

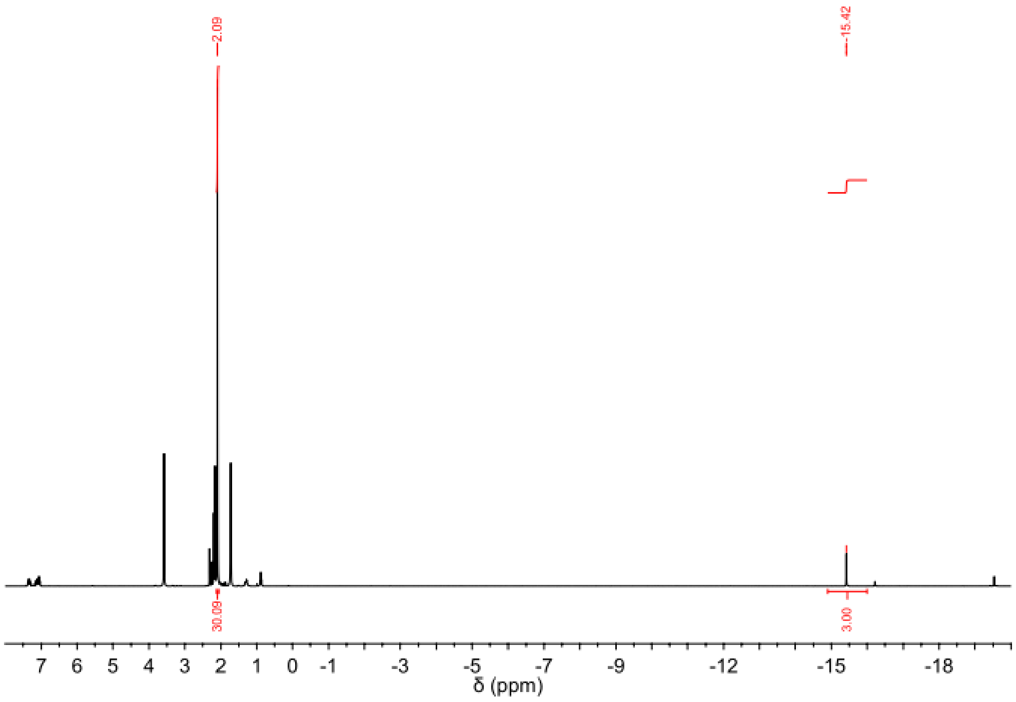


**Figure S16.** $^{1}$H NMR spectrum (300 MHz, $C_6D_6$, 296 K) for compound **4**

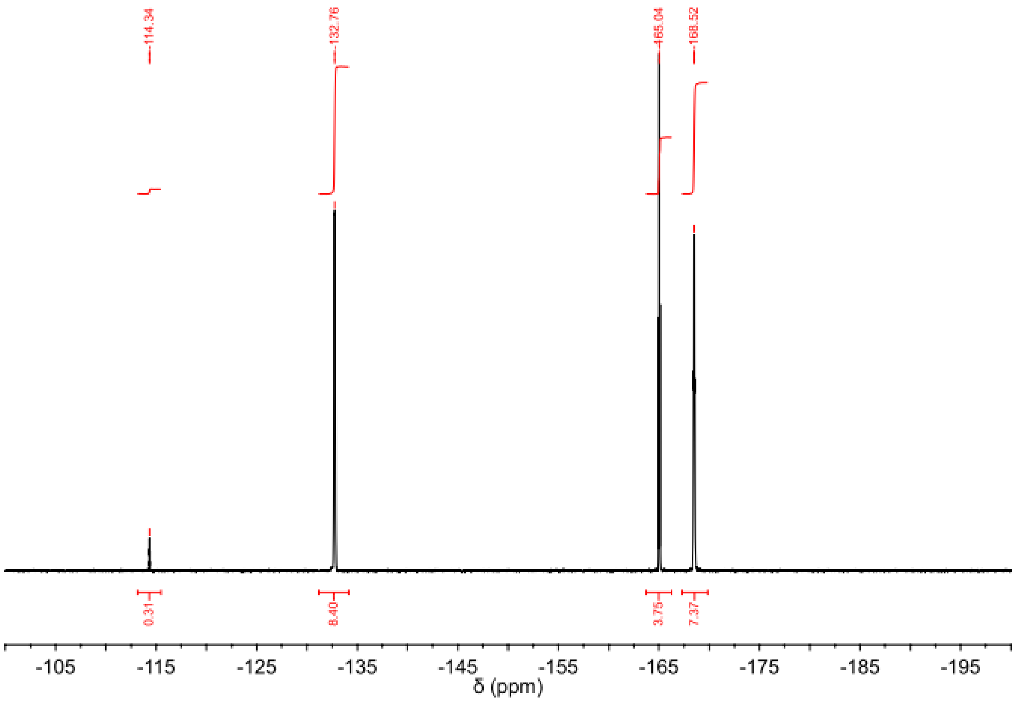


**Figure S17.** $^{19}F$ NMR spectrum (282 MHz, $C_6D_6$, 296 K) for compound **4**

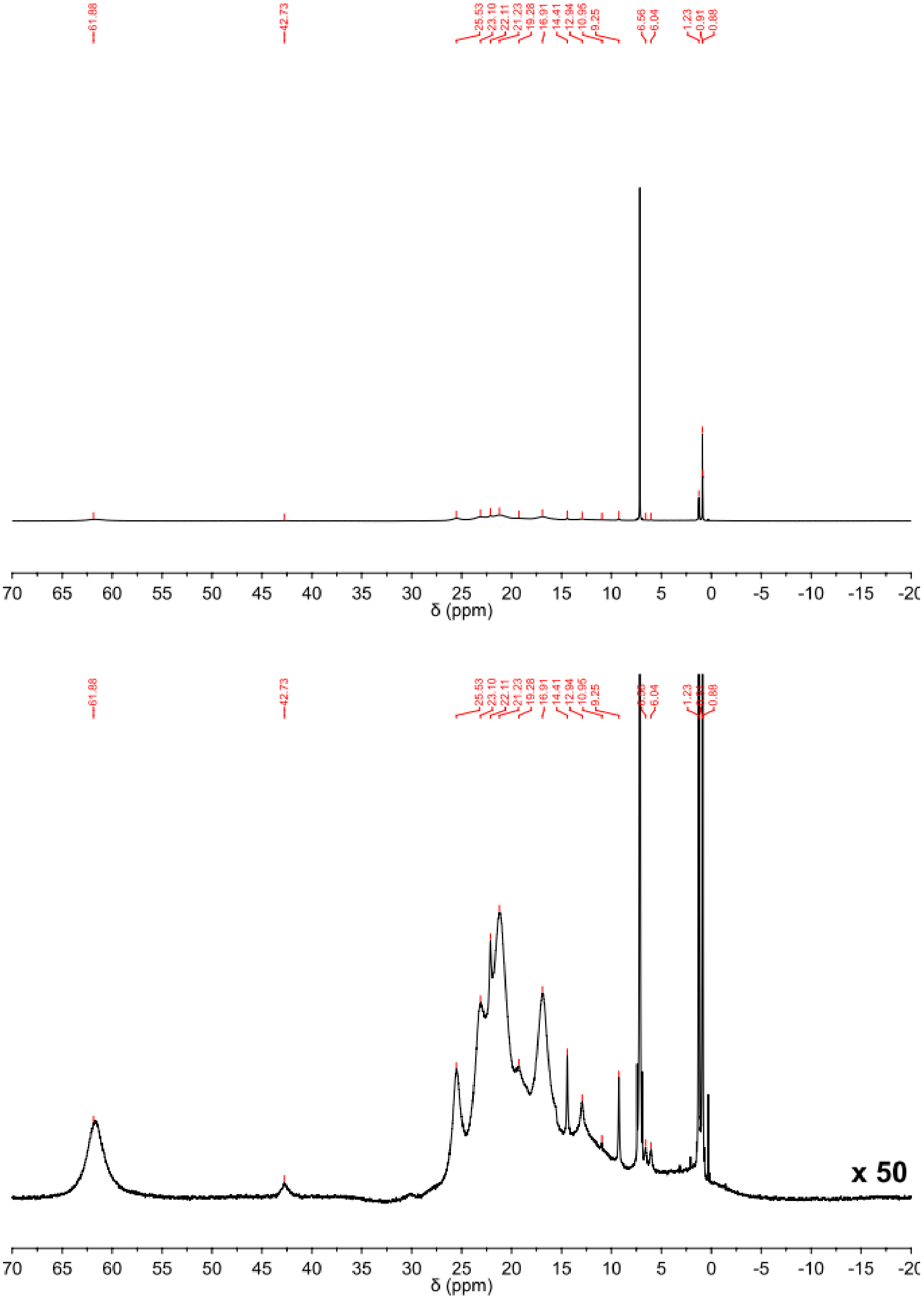


**Figure S18.** $^{1}$H NMR spectrum (300 MHz, $C_6D_6$, 296 K) for compound **5-Mn** immediately after dissolving in the deuterated solvent (top) and 50-fold vertical zoom to emphasize resonances broadened due to paramagnetism (bottom). Resonances at 0.88 and 1.23 ppm from trace amounts of *n*-pentane, resonance at 0.91 ppm from beginning release of neopentane

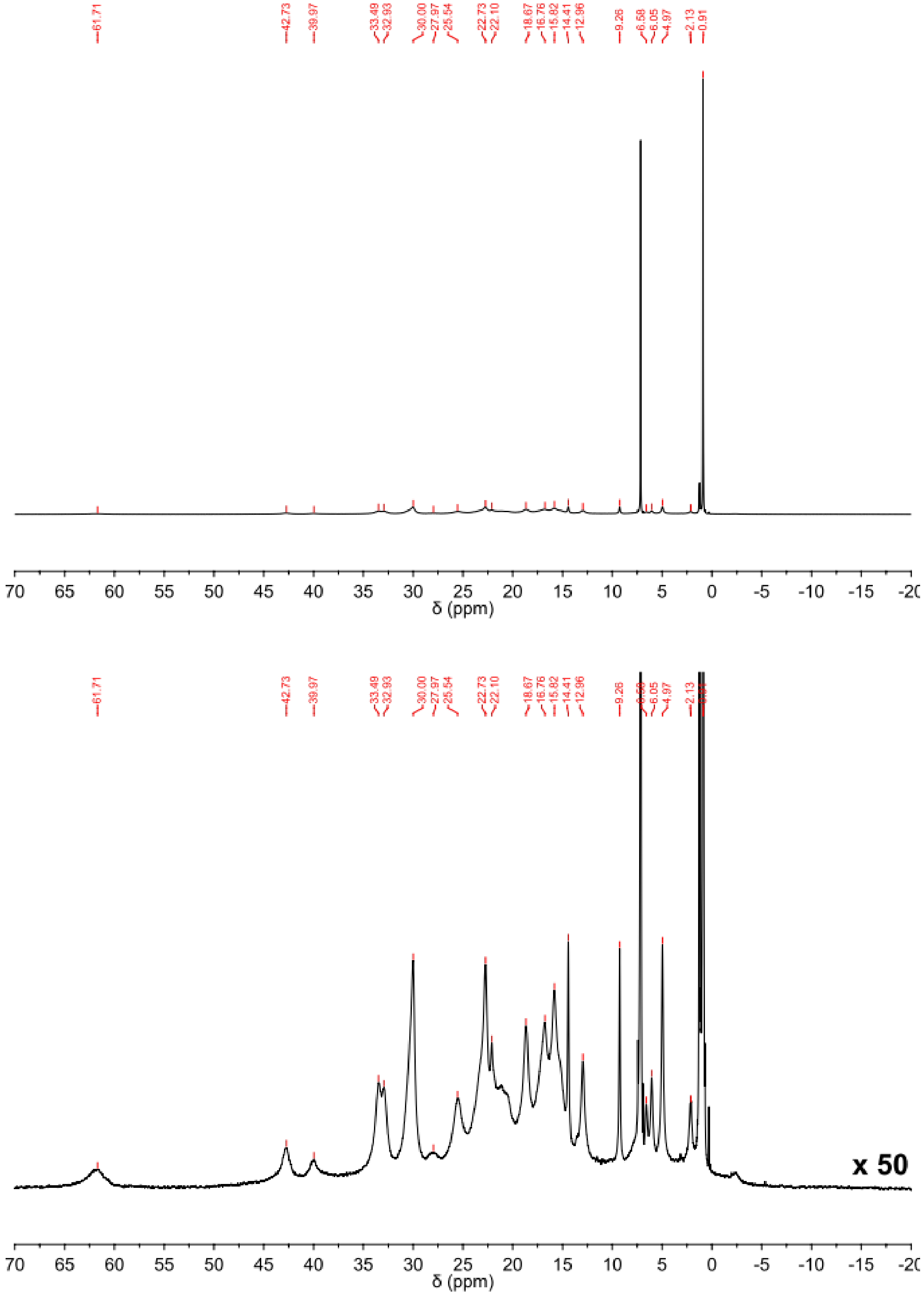


**Figure S19.** $^{1}$H NMR spectrum (300 MHz, $C_6D_6$, 296 K) for compound **5-Mn** two hours after dissolving in the deuterated solvent (top) and 50-fold vertical zoom to emphasize resonances broadened due to paramagnetism (bottom). The intensified resonance at 0.91 ppm illustrates the fast release of neopentane into the solution.

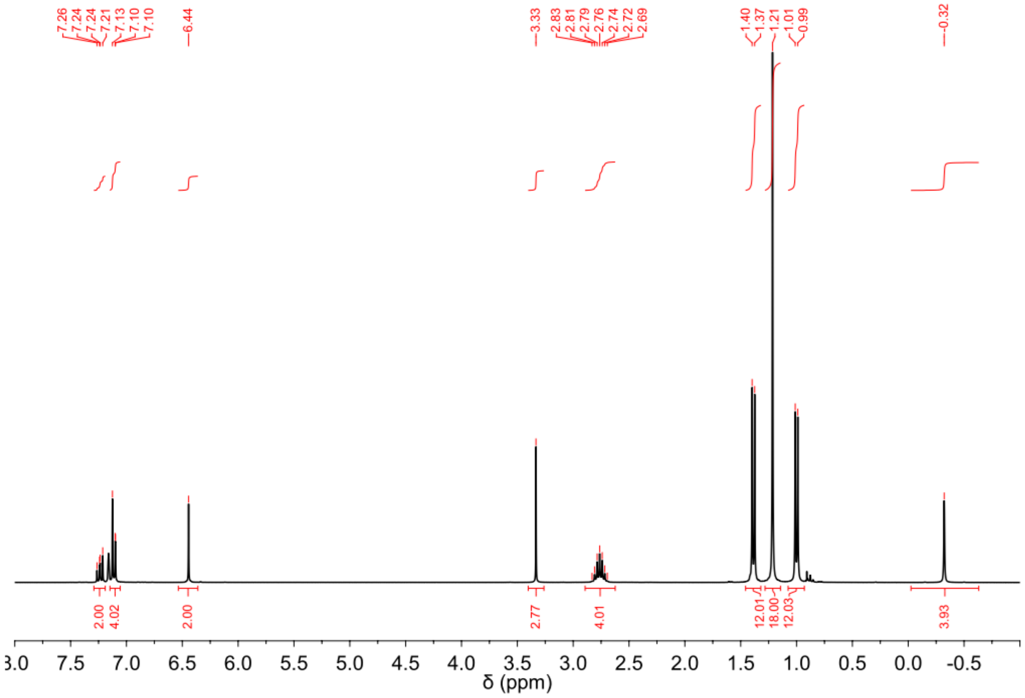


**Figure S20.** $^1$H NMR spectrum (300 MHz, $C_6D_6$, 295 K) for compound **6-Mg**. Resonance at 3.33 ppm due to residual amounts of 1,4-dioxane

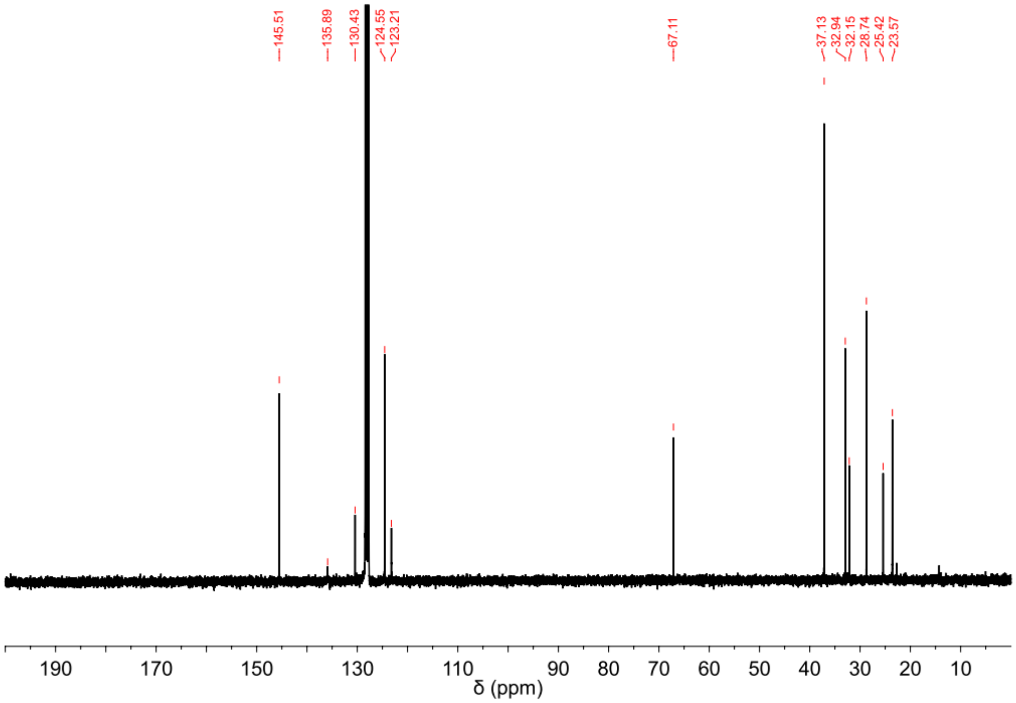


**Figure S21.** $^{13}C\{^1H\}$ NMR spectrum (75 MHz, $C_6D_6$, 295 K) for compound **6-Mg**. Resonance at 67.11 ppm due to residual amounts of 1,4-dioxane

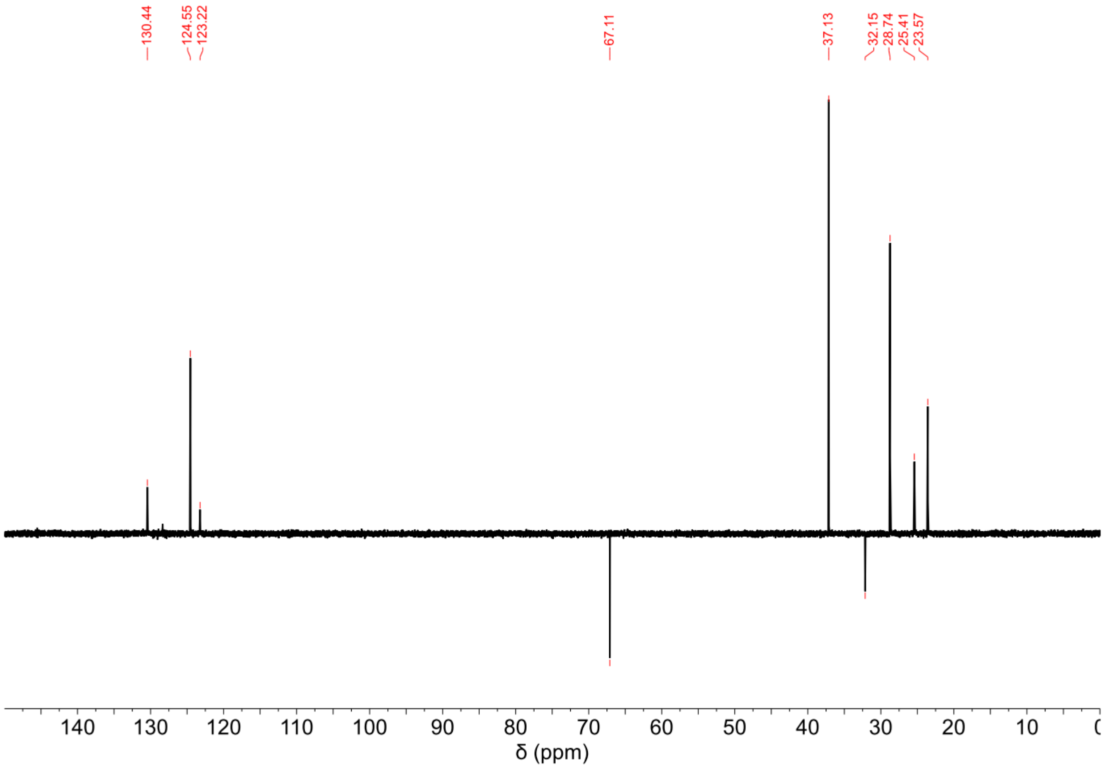


**Figure S22.** $^{13}C\{^{1}H\}$ DEPT-135 NMR spectrum (100 MHz, $C_6D_6$, 293 K) for compound **6-Mg**

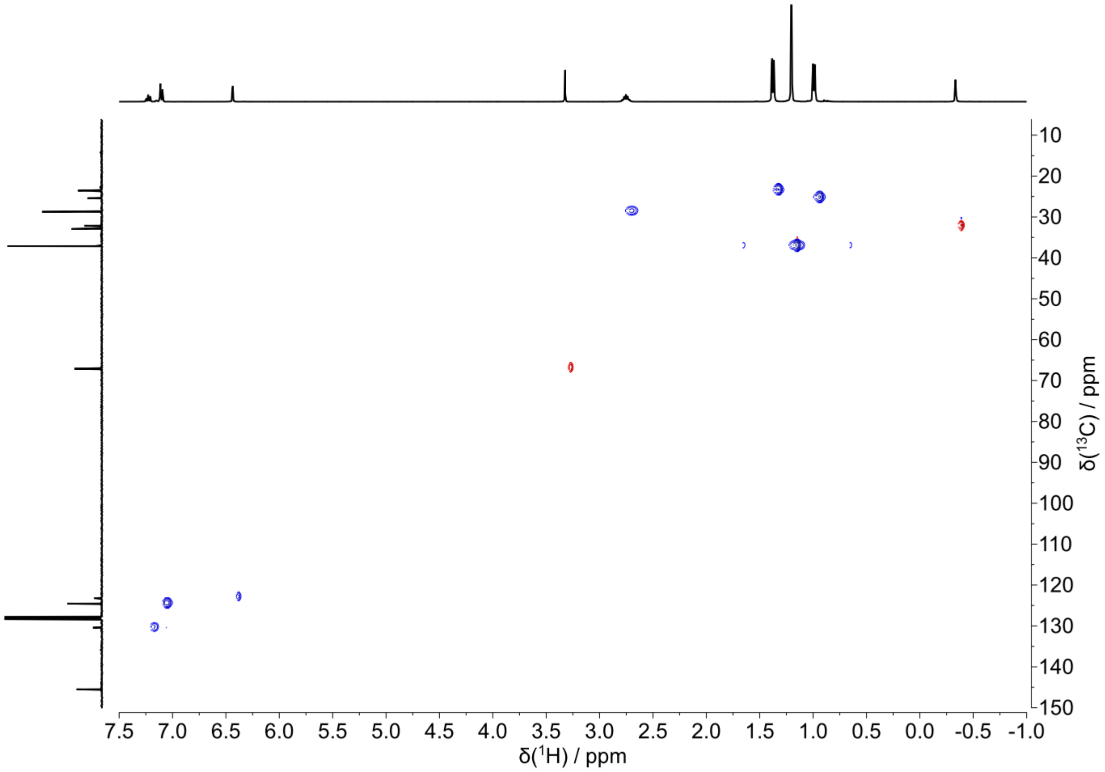


**Figure S23.** $^{1}H$-$^{13}C$ HSQC NMR spectrum (400 MHz, $C_6D_6$, 293 K) for compound **6-Mg**

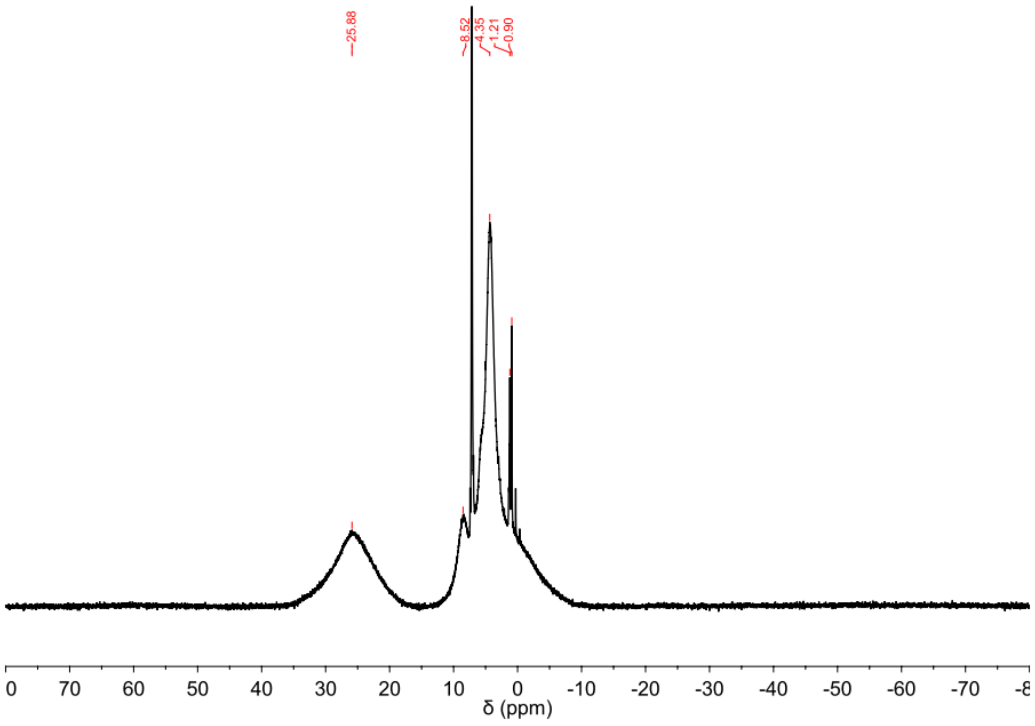


**Figure S24.** $^{1}H$ NMR spectrum (300 MHz, $C_6D_6$, 295 K) for compound **6-Mn**. Resonances at 0.90 and 1.21 ppm due to trace amounts of *n*-pentane in the purified product

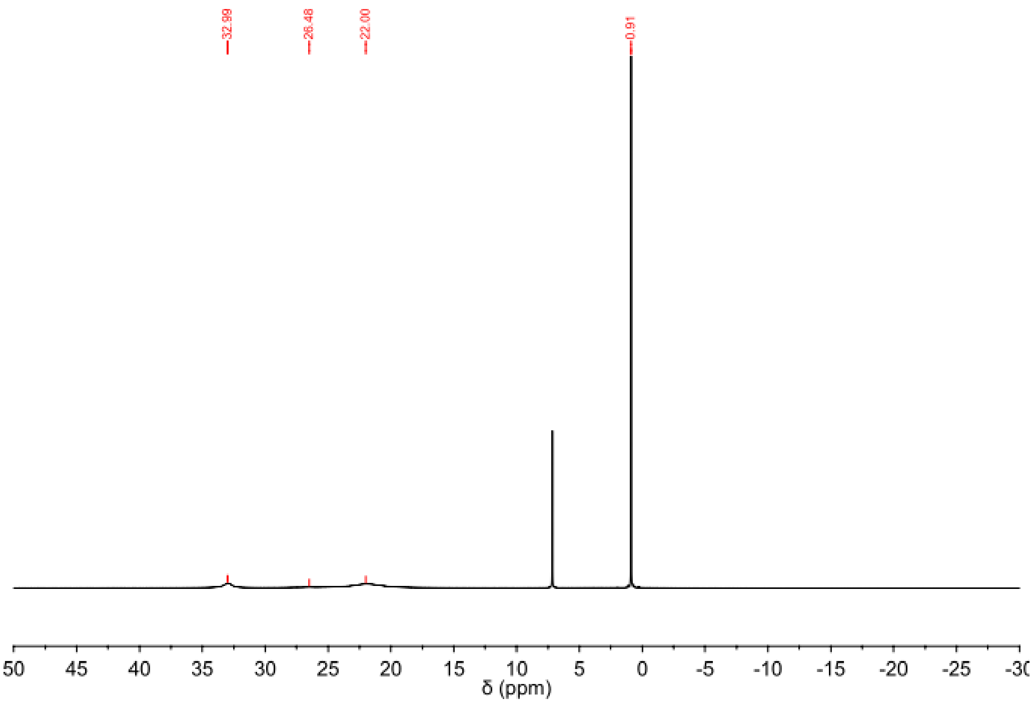


**Figure S25.** $^{1}H$ NMR spectrum (300 MHz, $C_6D_6$, 295 K) for compound **7-Mn** formed *in situ* from **1-Mn** and 8 eq. of [Cp*Ir$H_4$]. Sharp resonance at 0.91 ppm from release of neopentane eliminated during the protonolysis between the monometallic precursors

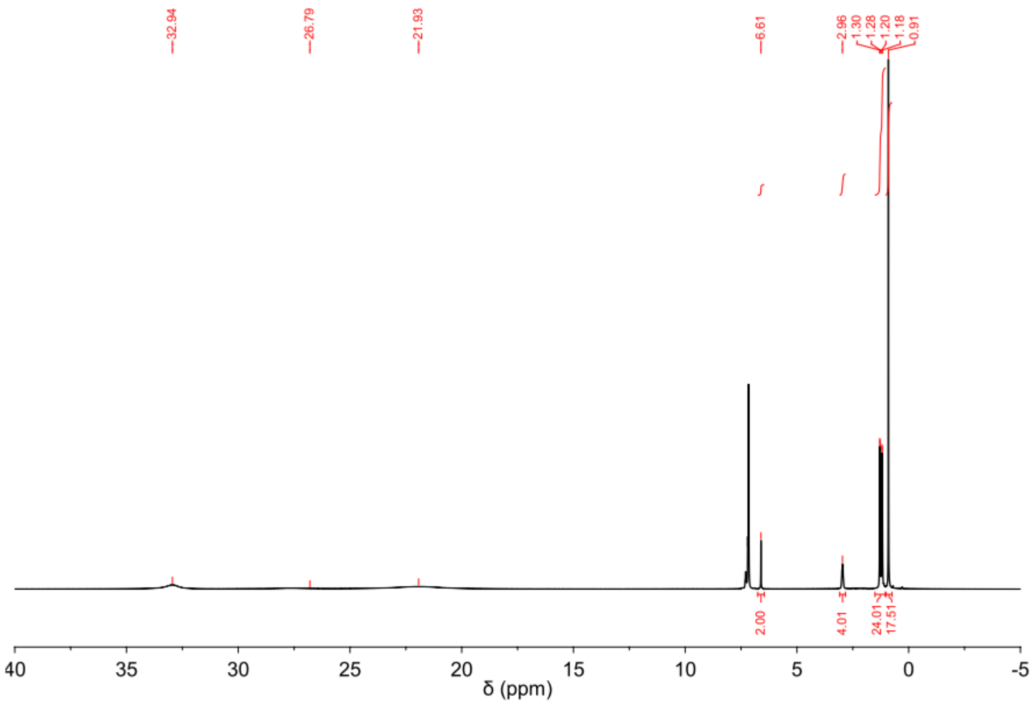


**Figure S26.** $^{1}$H NMR spectrum (300 MHz, $C_6D_6$, 295 K) for compound **7-Mn** formed *in situ* from **6-Mn**. Sharp resonances due to neopentane elimination (0.91 ppm) and uncoordinated NHC ligand

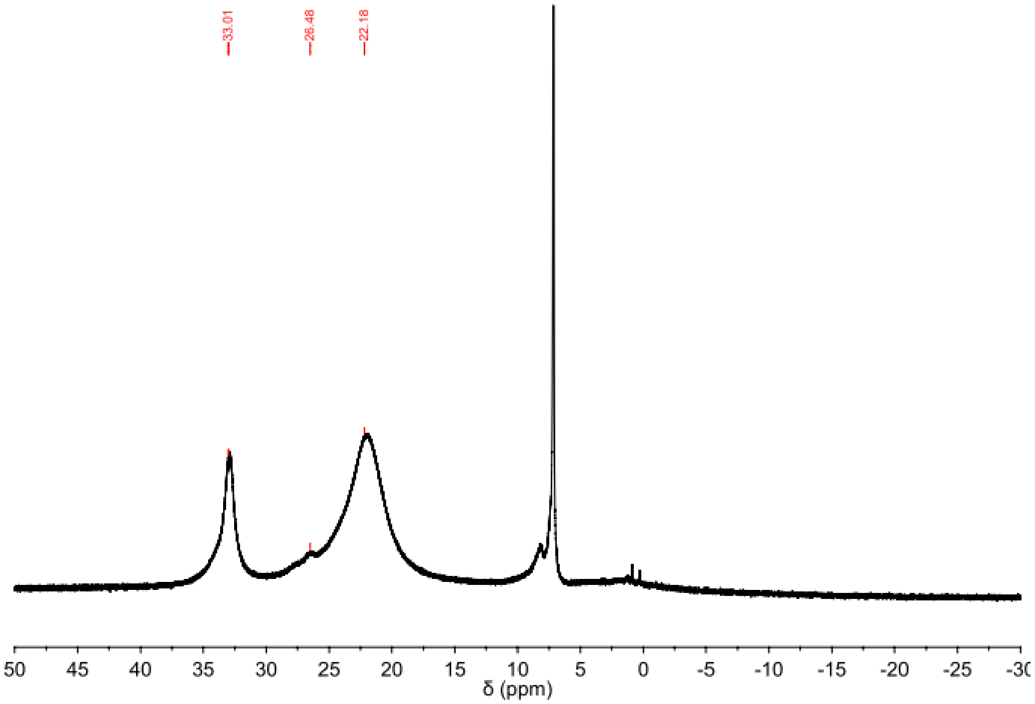


**Figure S27.** $^{1}$H NMR spectrum (300 MHz, $C_6D_6$, 295 K) for isolated **7-Mn** dried under vacuum

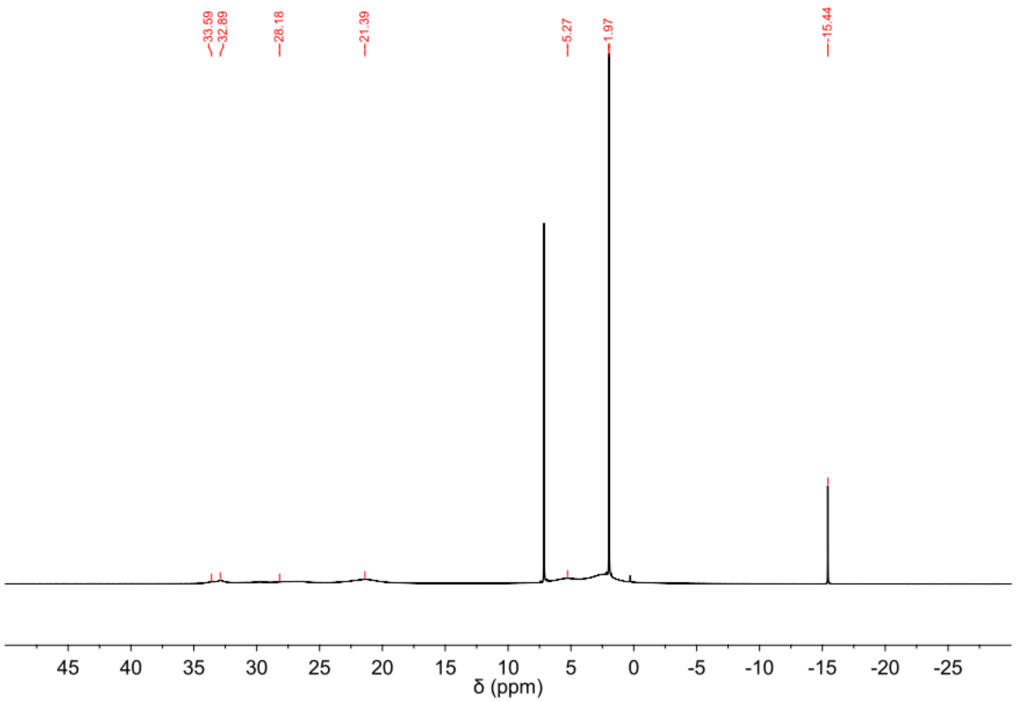


**Figure S28.** $^{1}$H NMR spectrum (300 MHz, $C_6D_6$, 296 K) for compound **8-Mn** formed *in situ* from **7-Mn** by addition of 1 eq. THF and 1 eq. tris(*tert*-butoxy)silanol based on Mn. Sharp resonances at 1.97 and -15.44 ppm from release of 1 eq. [Cp*$IrH_4$]

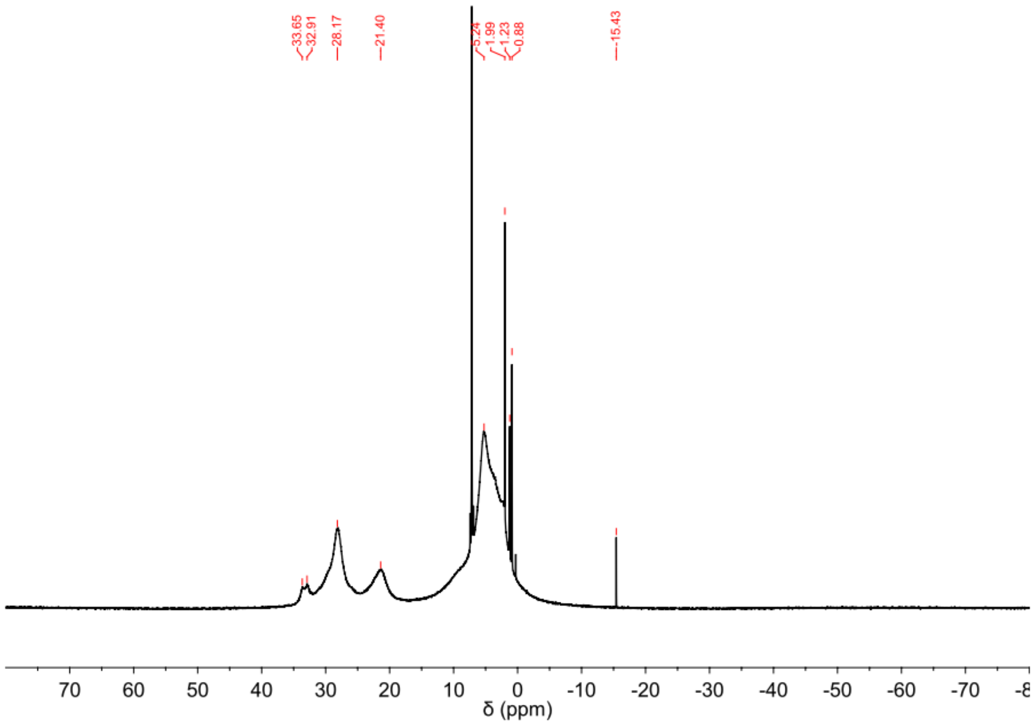


**Figure S29.** $^{1}$H NMR spectrum (300 MHz, $C_6D_6$, 295 K) for isolated, crystalline **8-Mn** dried under vacuum. Resonances at 0.88, 1.23 ppm (*n*-pentane) and -15.43, 1.99 ppm ([Cp*$IrH_4$]) due to trace impurities in the purified product

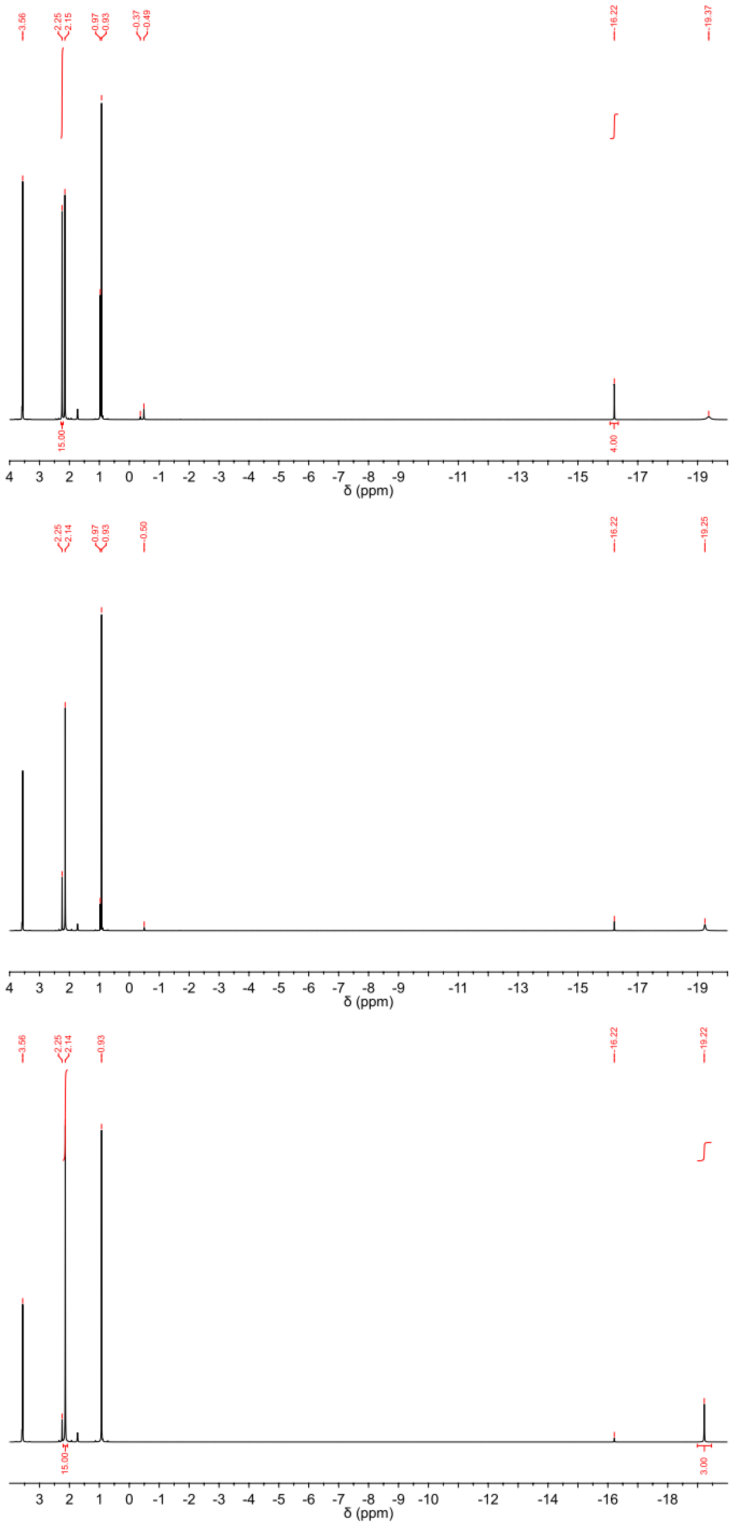


**Figure S30.** $^{1}$H NMR spectra (300 MHz, THF-$d_8$, 295 K) for the reaction of [Mg(CH$_2$*t*Bu)$_2$(1,4-dioxane)]$_n$ with 2 equiv. [Cp*IrH$_4$] (15 H at 2.25 ppm, 4 H at -16.22 ppm) at t = 10 min (top), 1 h (center) and 16 h (bottom), giving a single hydride species (15 H at 2.14 ppm, 3 H at -19.22 ppm) under formation of neopentane (0.93 ppm)

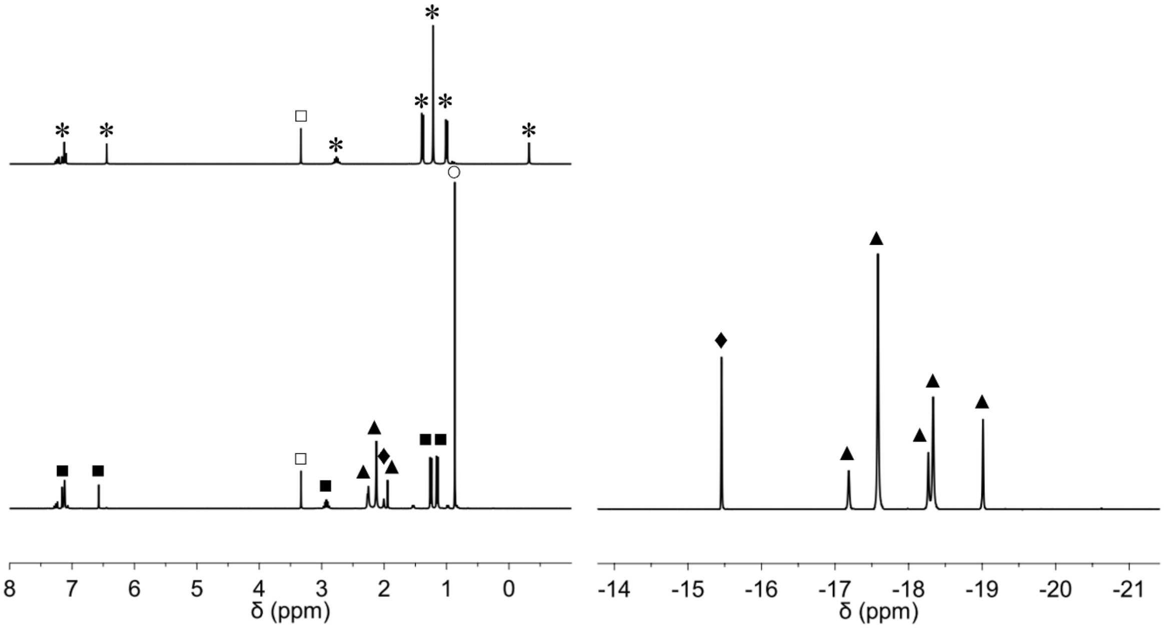


**Figure S31.** $^1$H NMR spectra (300 MHz, $C_6D_6$, 296 K) for compound **6-Mg** (top) and for the reaction of **6-Mg** with 2.3 equiv. [Cp*IrH$_4$] with vertical zoom of the hydride region (bottom). Identified compounds: **6-Mg** (*), 1,4-dioxane (□), uncoordinated NHC ligand IPr (■), 0.3 equiv. excess [Cp*IrH$_4$] (♦), neopentane (○), putative heterobimetallic hydride species (▲)

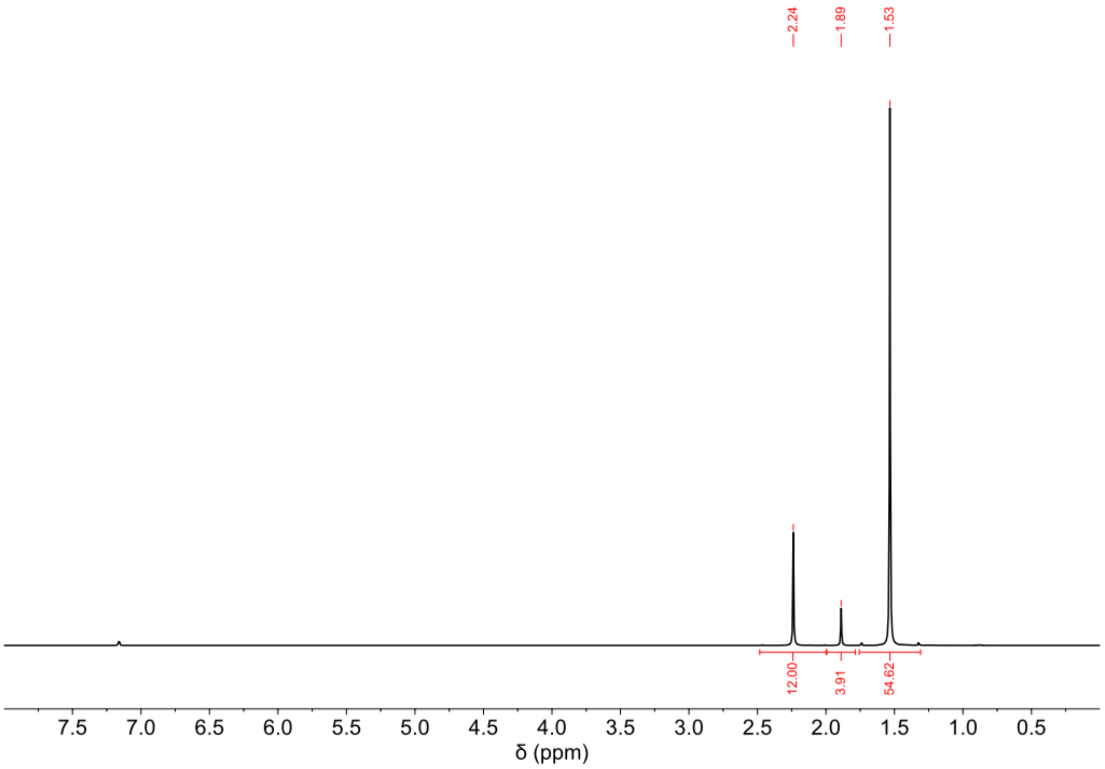


**Figure S32.** $^{1}H$ NMR spectrum (300 MHz, $C_6D_6$, 295 K) for compound [Mg(tmeda)(OSi{O*t*Bu}$_3$)$_2$]

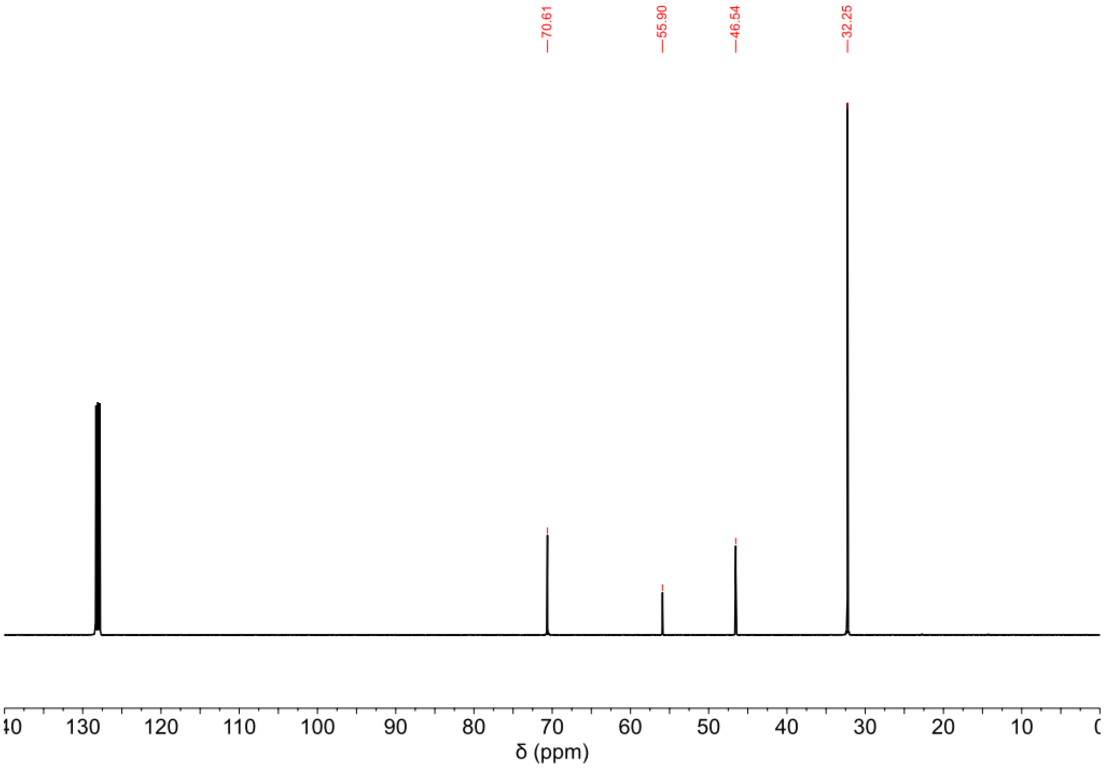


**Figure S33.** $^{13}C\{^{1}H\}$ NMR spectrum (75 MHz, $C_6D_6$, 295 K) for compound [Mg(tmeda)(OSi{O*t*Bu}$_3$)$_2$]

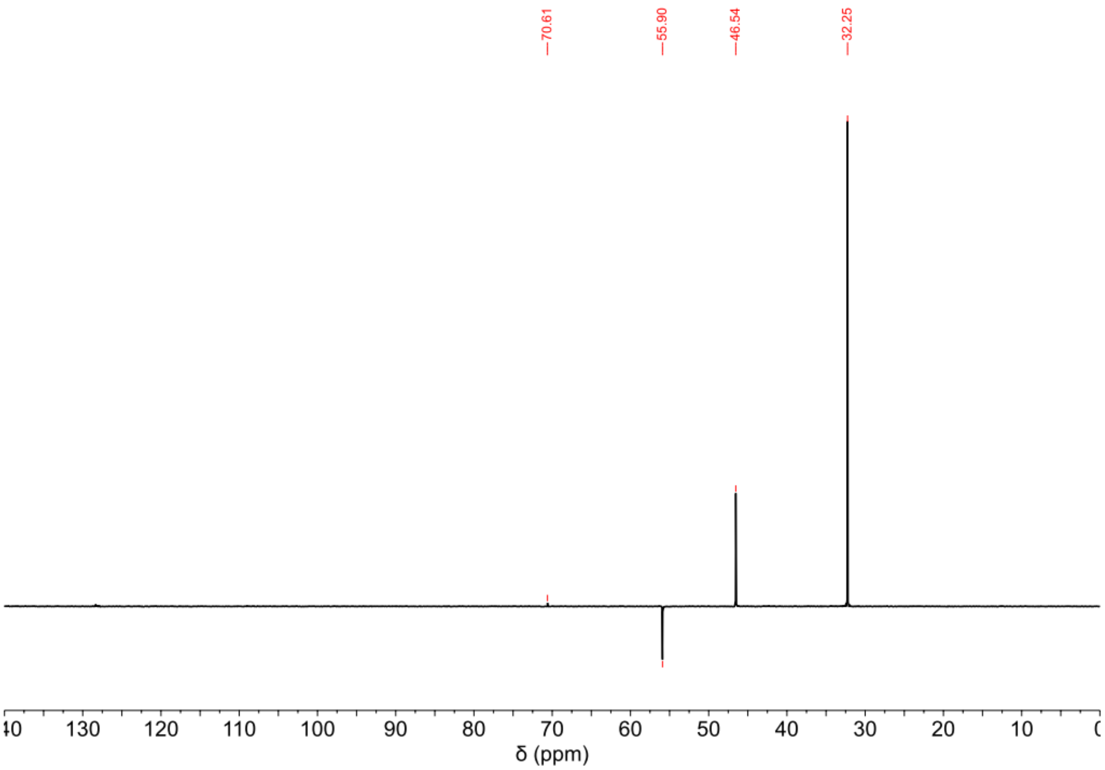


**Figure S34.** $^{13}C\{^{1}H\}$ DEPT-135 NMR spectrum (100 MHz, $C_6D_6$, 293 K) for compound [Mg(tmeda)(OSi{O*t*Bu}$_3$)$_2$]

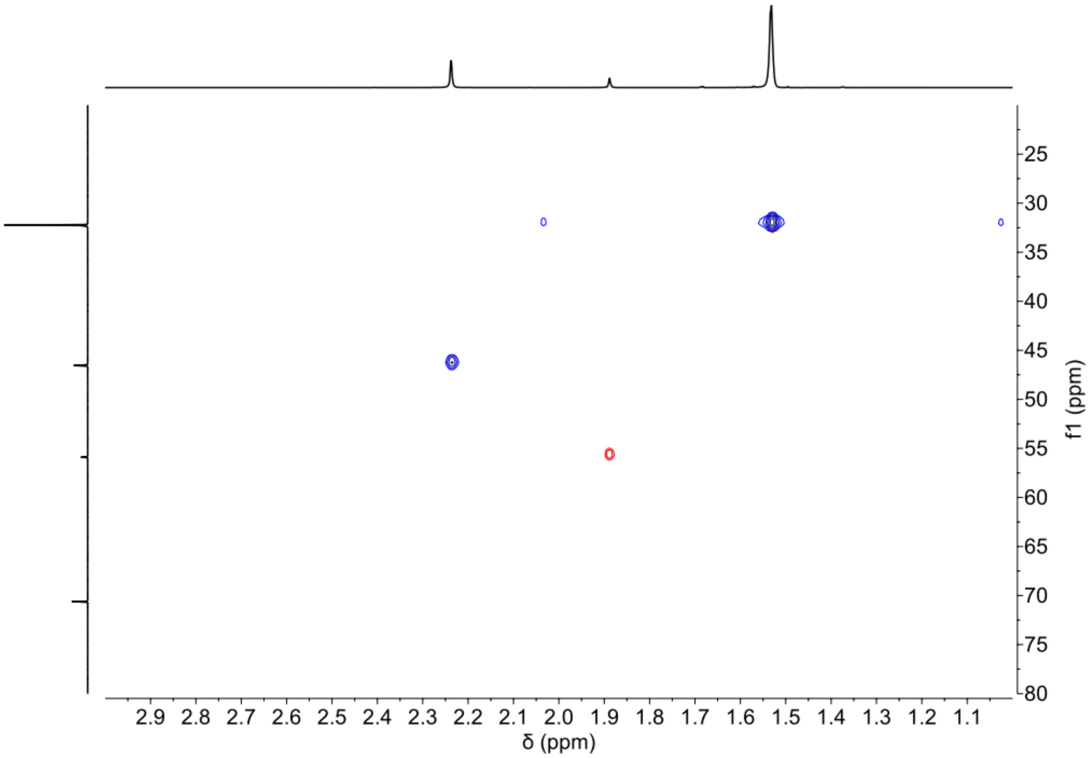


**Figure S35.** $^{1}H$-$^{13}C$ HSQC NMR spectrum (400 MHz, $C_6D_6$, 293 K) for compound [Mg(tmeda)(OSi{O*t*Bu}$_3$)$_2$]

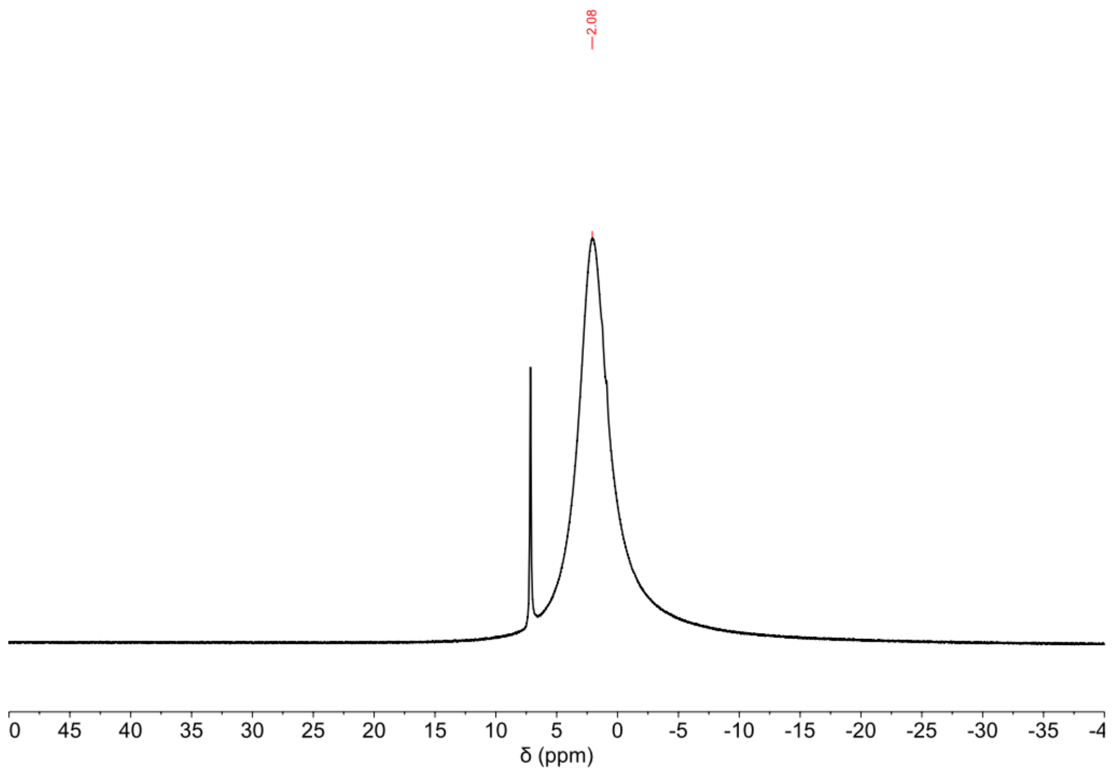


**Figure S36.** $^{1}$H NMR spectrum (300 MHz, $C_6D_6$, 295 K) for compound [Mn(tmeda)(OSi{O*t*Bu}$_3$)$_2$]

Diffuse reflectance infrared Fourier-transform (DRIFT) spectroscopy data

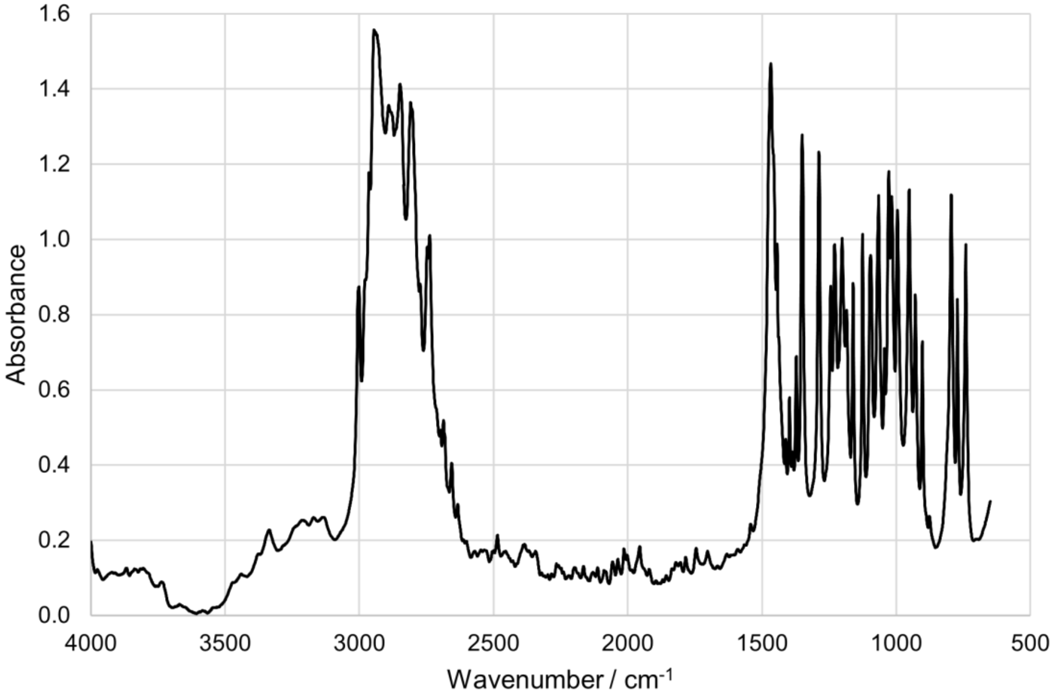


**Figure S37.** DRIFT spectrum for compound **1-Mg** (293 K)

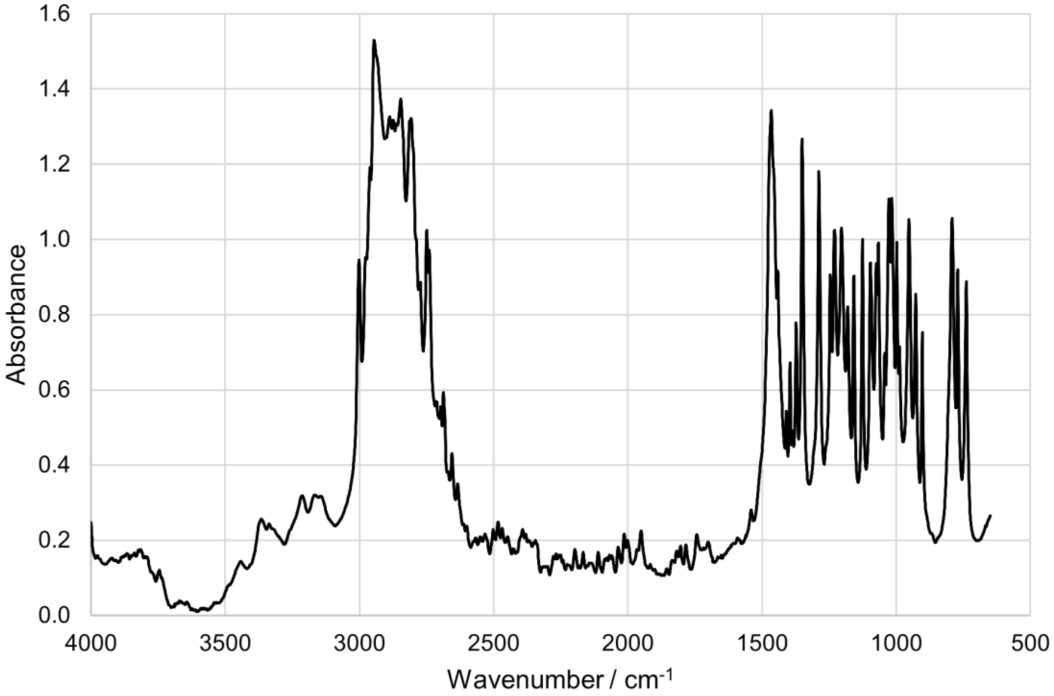


**Figure S38.** DRIFT spectrum for compound **1-Mn** (293 K)

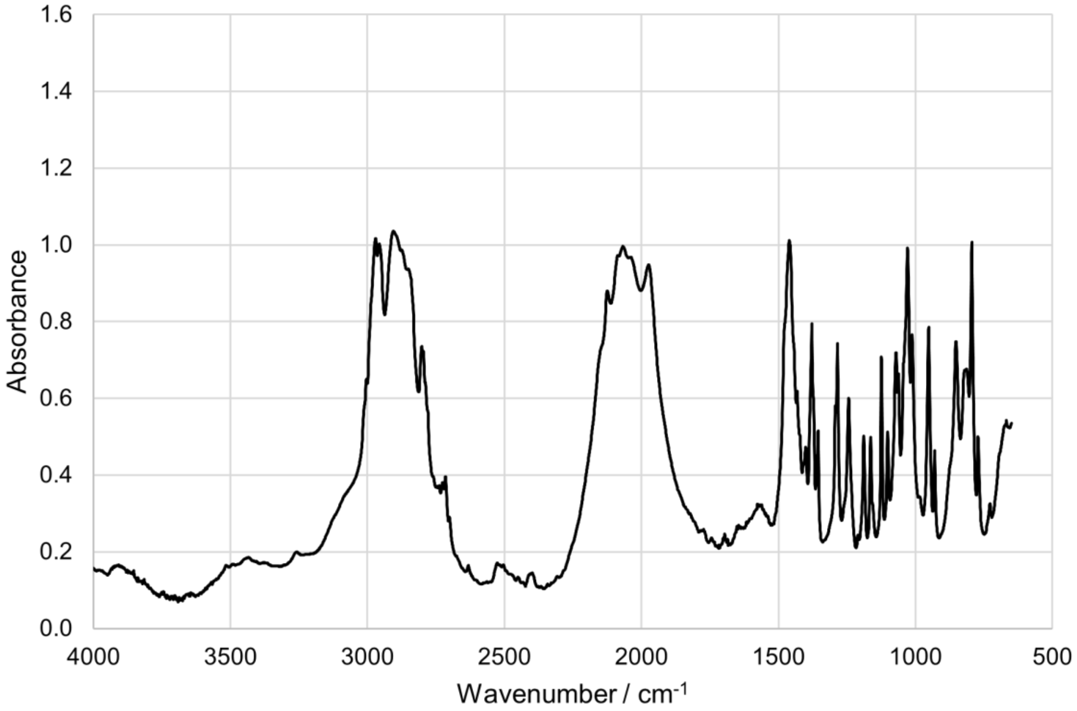


**Figure S39.** DRIFT spectrum for compound **2-Mg** (293 K)

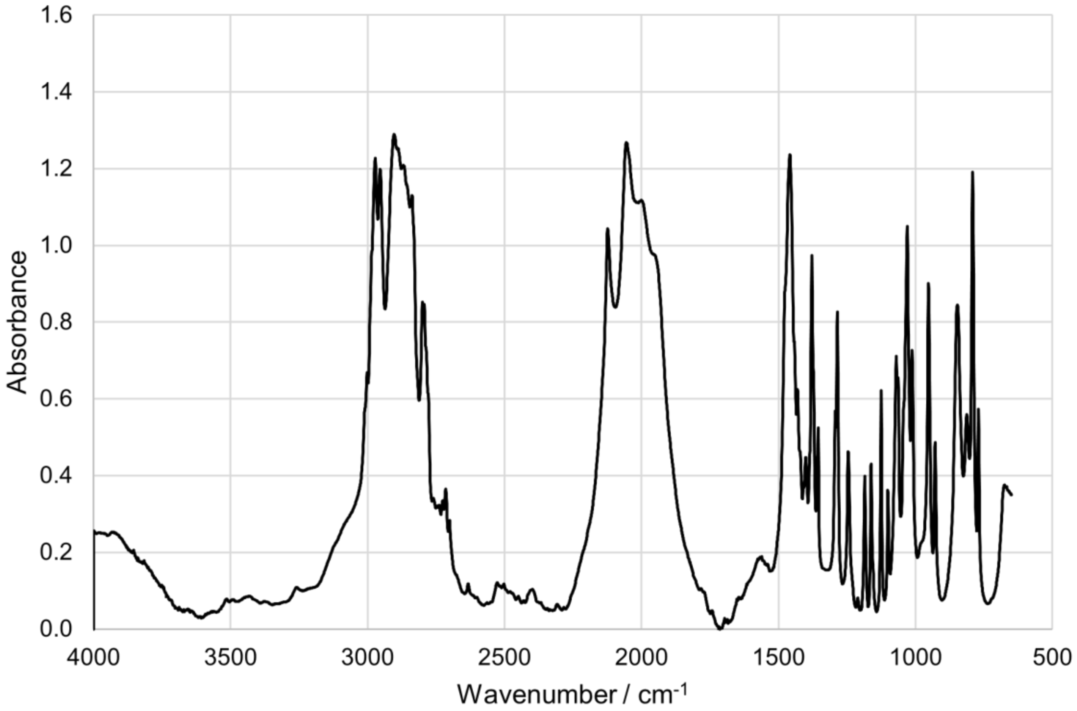


**Figure S40.** DRIFT spectrum for compound **2-Mn** (293 K)

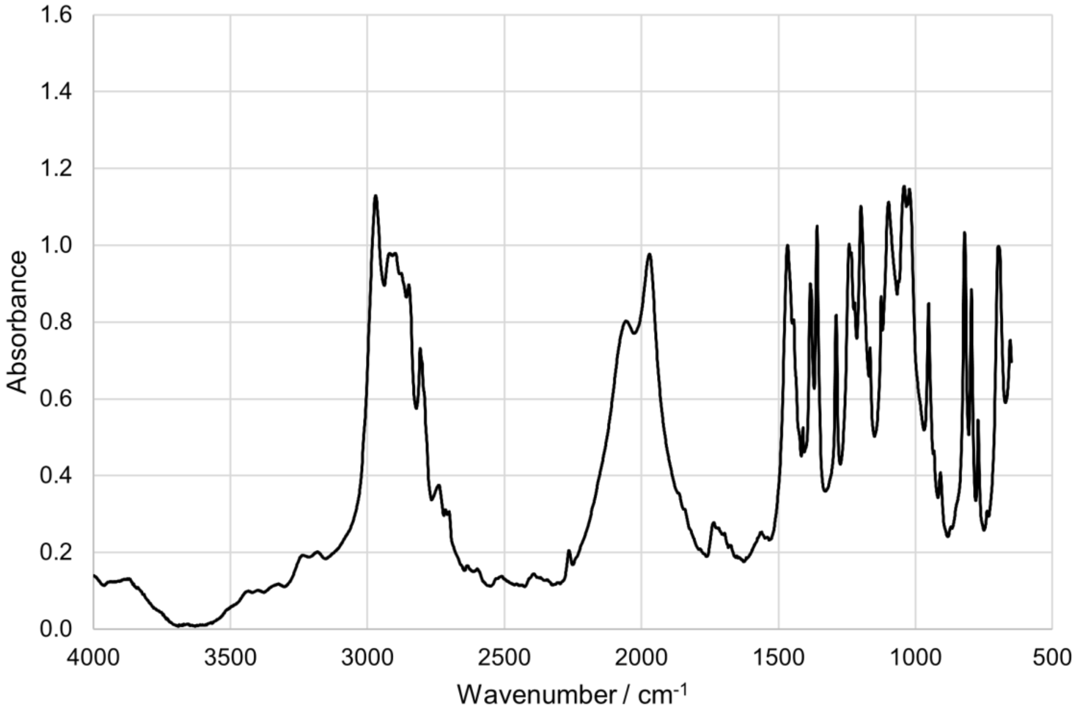


**Figure S41.** DRIFT spectrum for compound **3-Mg** (293 K)

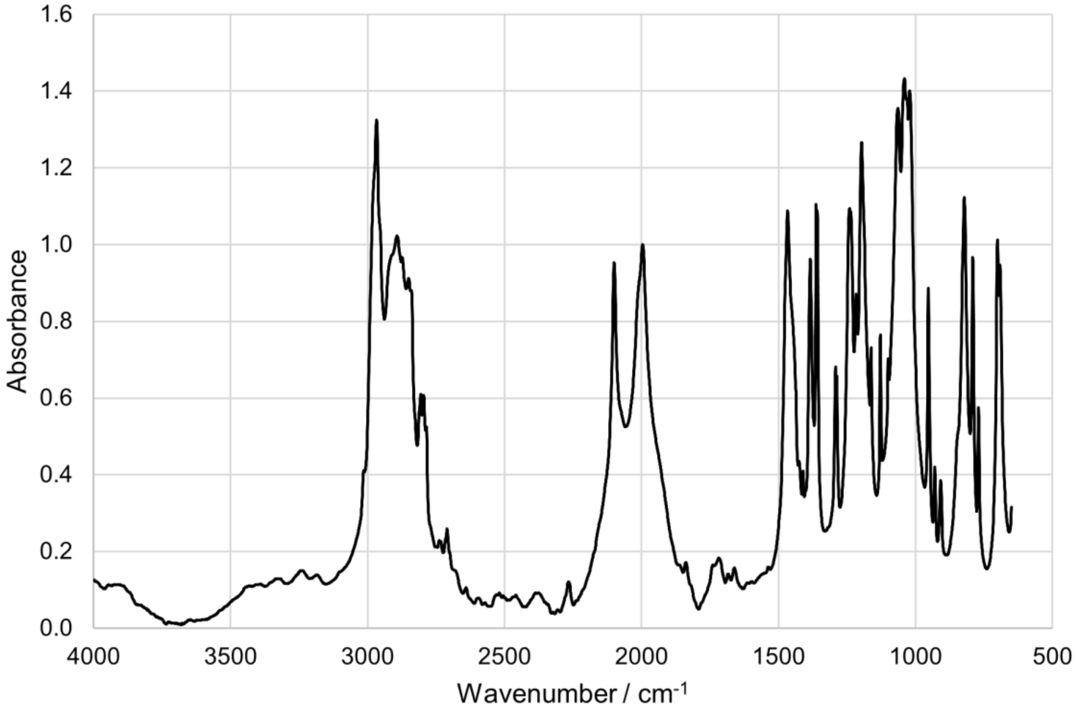


**Figure S42.** DRIFT spectrum for compound **3-Mn** (293 K)

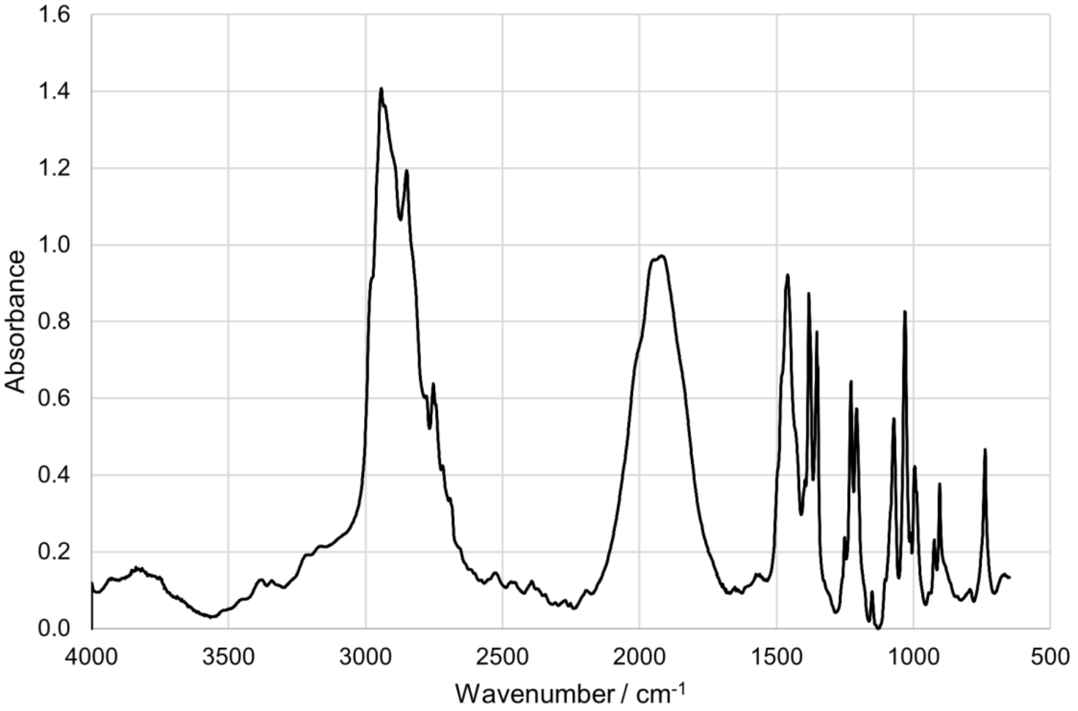


**Figure S43.** DRIFT spectrum for compound **5-Mn** (293 K)

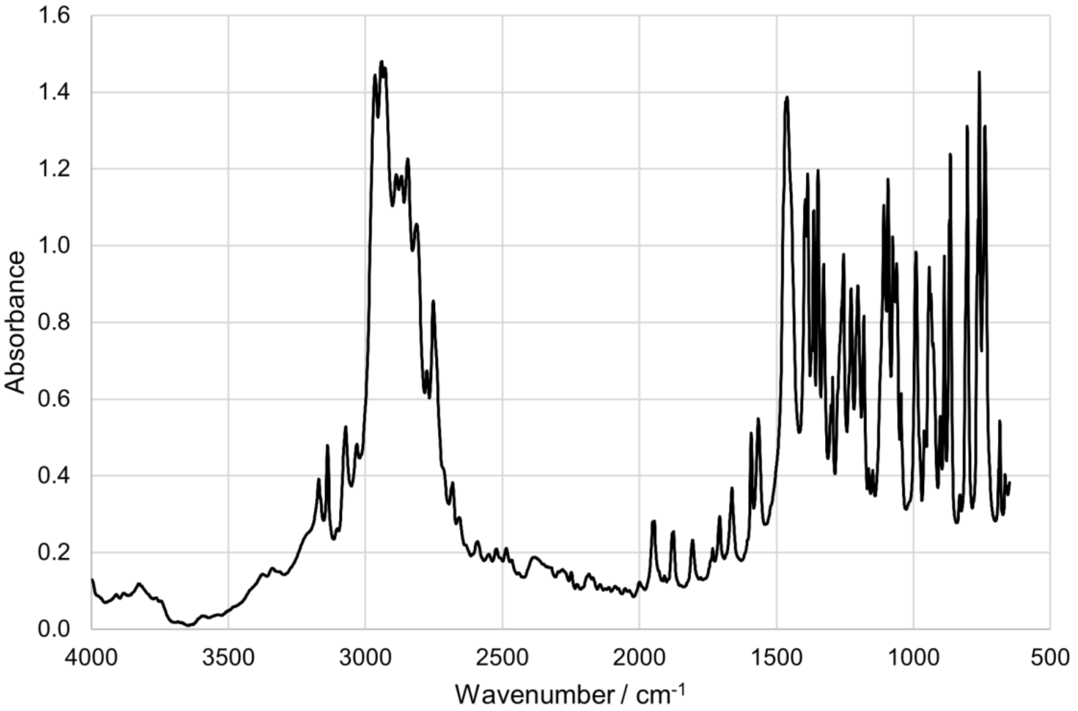


**Figure S44.** DRIFT spectrum for compound **6-Mg** (293 K)

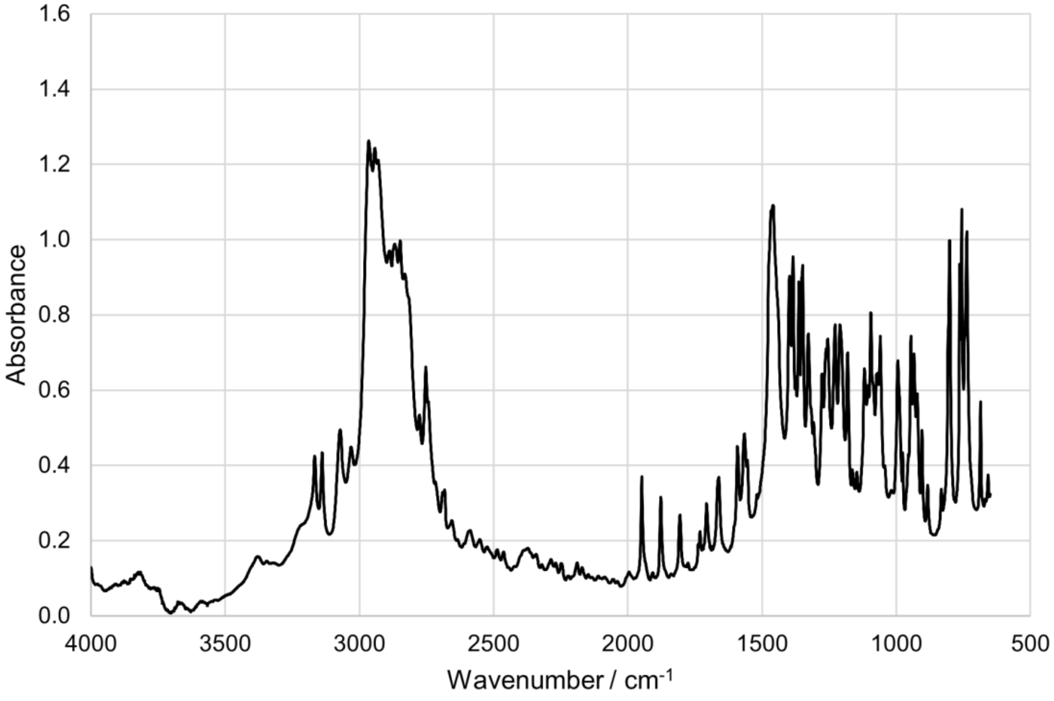


**Figure S45.** DRIFT spectrum for compound **6-Mn** (293 K)

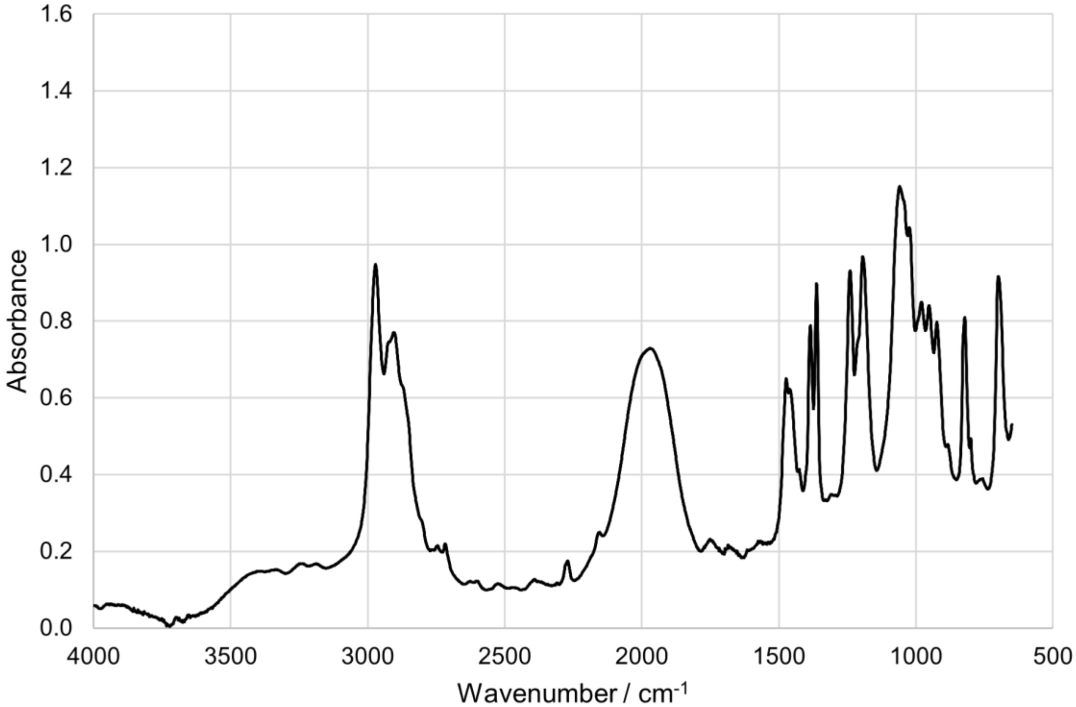


**Figure S46.** DRIFT spectrum for compound **8-Mn** (293 K)

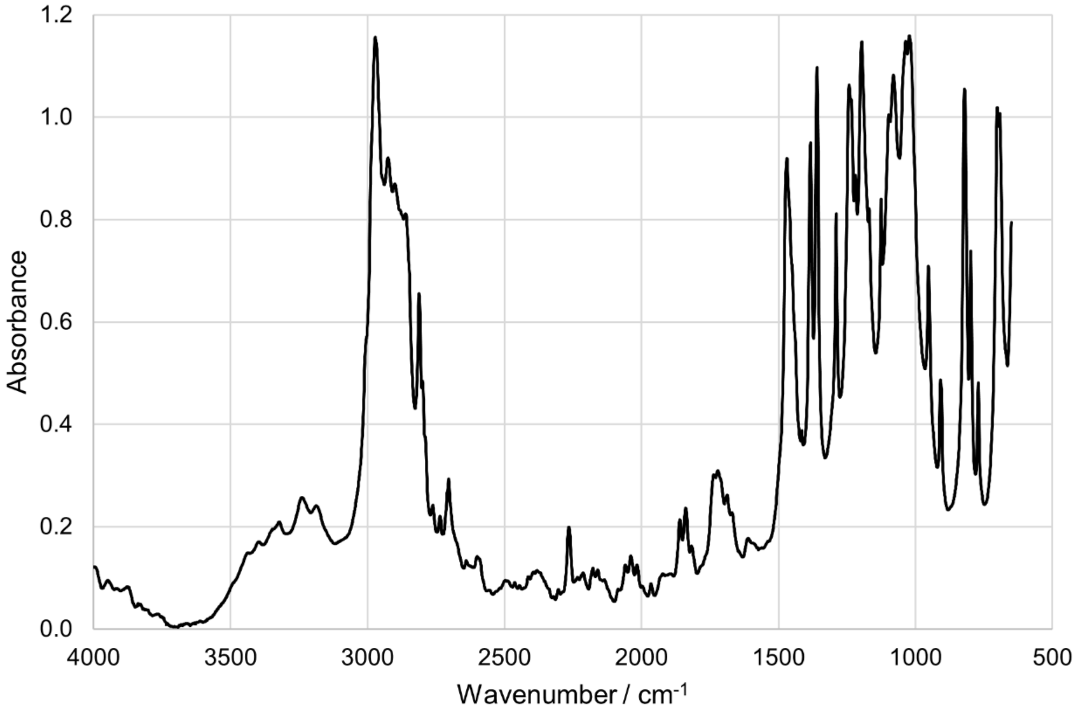


**Figure S47.** DRIFT spectrum for compound [Mg(tmeda)(OSi{O*t*Bu}$_3$)$_2$] (293 K)

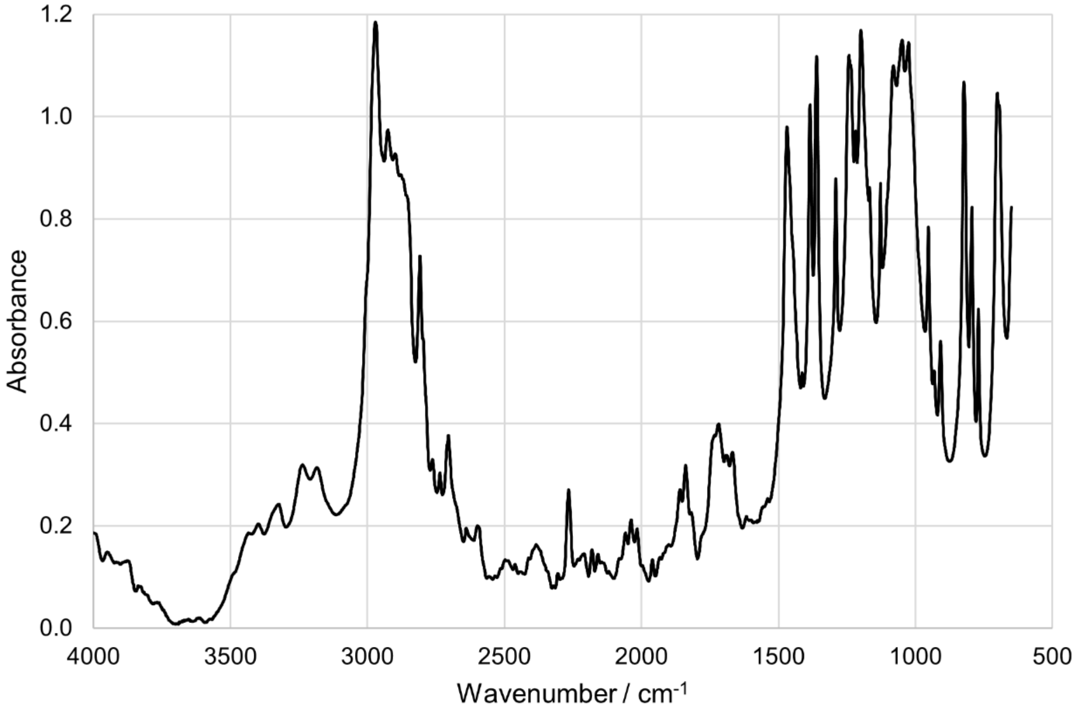


**Figure S48.** DRIFT spectrum for compound [Mn(tmeda)(OSi{O*t*Bu}$_3$)$_2$] (293 K)

# Crystallographic data

Single crystal samples were prepared in a glove box under an argon atmosphere and transported to the diffractometer in a sealed scintillation vial. Suitable crystals were selected and mounted on a MITIGEN holder in perfluoroether oil on a XtaLAB Synergy, Dualflex, HyPix-Arc 100 diffractometer. The crystals were kept at a steady T = 100 K during data collection. The structure was solved with the ShelXT[1] solution program using dual methods and by using Olex2 1.5-ac6-020[2] as the graphical interface. The model was refined with ShelXL 2018/3[3] using full matrix least squares minimization on F2.

Note that the numbers and positions of the terminal and bridging hydride ligands in the structures of **2-Mg**, **2-Mn**, **3-Mg**, **3-Mn**, **4**, **5-Mn** and **8-Mn** could not be obtained from the difference Fourier maps and were thus not refined. Therefore, the numbers of hydrogen atoms in the certain formulas in Tables S2-S5 do not correspond to those of compounds **2-Mg**, **2-Mn**, **3-Mg**, **3-Mn**, **4**, **5-Mn** and **8-Mn** as they appear in the main article.

The residual electron density peaks of +5.08 and +4.82 $e$ Å$^{-3}$ in the crystal structure of **4** are located in the vicinity of the two iridium atoms (Ir1 and Ir2). Given the high atomic number and strong X-ray scattering of iridium, such residual density is typical and is attributed to minor imperfections in the modelling of core electron density and Fourier truncation effects. No chemically meaningful unmodelled features are indicated in the difference Fourier map.

The crystal structure of **5-Mn** has a large reported maximum positive residual density of 5.40 $e$ Å$^{-3}$ located 0.88 Å from Ir1, and thus lies in the immediate vicinity of the heavy iridium atom. Such features are typical for heavy-atom structures and arise from minor imperfections in modelling of core electron density and Fourier truncation effects.

The crystal structure of **6-Mg** has a large reported maximum positive residual density of 2.23 $e$ Å$^{-3}$. The residual densities suggest that the entire molecules are disordered around two sites (around 5% disorder). We were not able to model this disorder and we recorded several single-crystals obtained by different crystallization conditions, but they all showed the same feature. This also leads to a relatively high $R_1$ and $wR_2$, but we believe that the overall structure is reliable enough for publication.

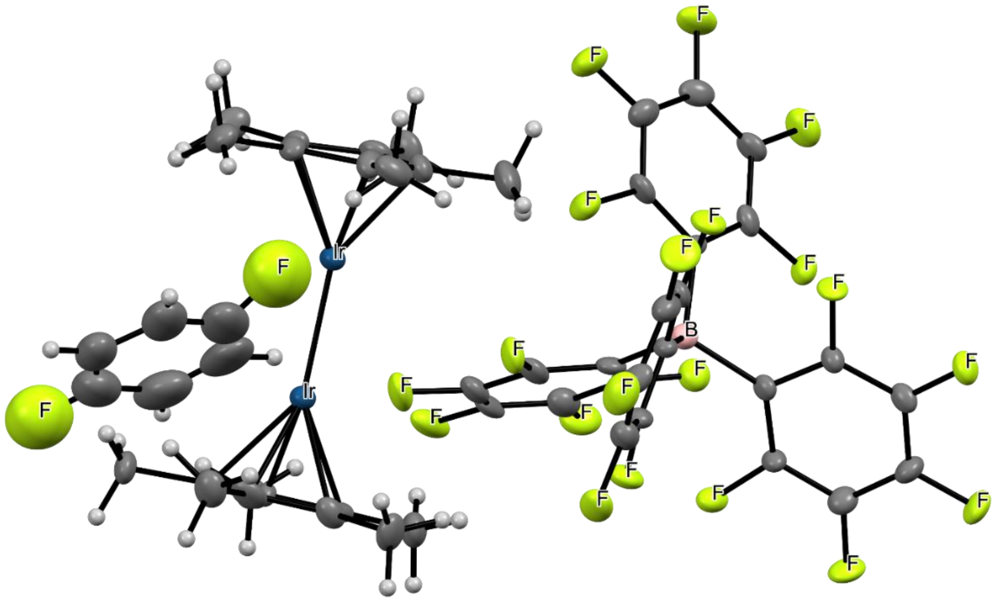


**Figure S49.** Solid-state molecular structure of **4**. Carbon atoms (gray) and hydrogen atoms (white) unlabeled for clarity.

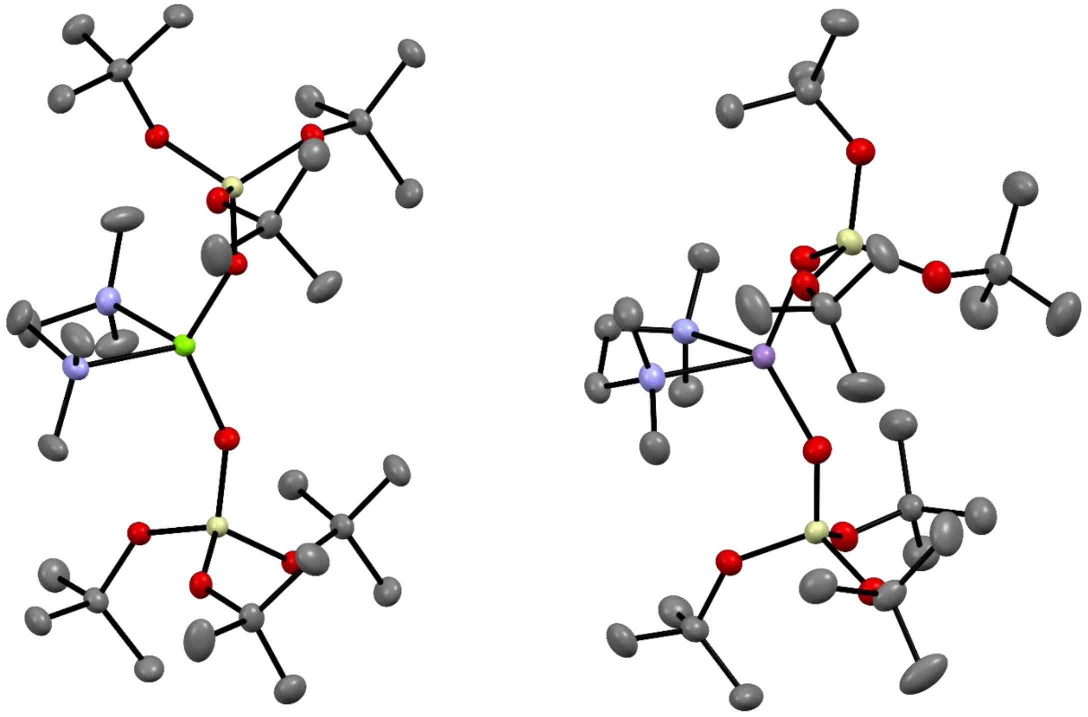

**Figure S50.** Solid-state molecular structures of [Mg(tmeda)(OSi{O*t*Bu}$_3$)$_2$] (left) and [Mn(tmeda)(OSi{O*t*Bu}$_3$)$_2$] (right) with thermal ellipsoids (ORTEP) drawn at the 50 % probability level. Hydrogen atoms omitted for clarity. Selected bond distances (Å) and angles (°): Mg−O 1.8583(7), Mn−O 1.944(3), Mg−N 2.191(2), Mn−N 2.258(3); Mg−O−Si 150.15(5), Mn−O−Si 126.9(1) and 148.2(2), N−Mg−N 84.12(5), N−Mn−N 81.6(1), O−Mg−O 127.17(5), O−Mn−O 128.0(1). These crystal structures closely resemble those of the analogous Cr and Co complexes reported previously.[4,5]

**Table S1.** Crystallographic parameters for the monometallic compounds **1-Mg** and **1-Mn**

| **Compound** | **1-Mg** | **1-Mn** |
|---|---|---|
| Formula | $C_{16}H_{38}MgN_2$ | $C_{16}H_{38}N_2Mn$ |
| $D_{calc.}$ / g cm$^{-3}$ | 0.970 | 1.078 |
| *m* / mm$^{-1}$ | 0.706 | 0.677 |
| Formula Weight | 282.79 | 313.42 |
| Color | colorless | colorless |
| Shape | block-shaped | block-shaped |
| Size / mm$^3$ | 0.19×0.10×0.05 | 0.66×0.52×0.30 |
| *T* / K | 100.01(10) | 100.00(16) |
| Crystal System | monoclinic | monoclinic |
| Space Group | *C*2/*c* | *C*2/*c* |
| *a* / Å | 17.2860(2) | 17.3230(4) |
| *b* / Å | 9.37130(10) | 9.3595(2) |
| *c* / Å | 12.4971(2) | 12.4436(3) |
| $\alpha$ / ° | 90 | 90 |
| $\beta$ / ° | 106.9810(10) | 106.840(2) |
| $\gamma$ / ° | 90 | 90 |
| *V* / Å$^3$ | 1936.17(4) | 1931.02(8) |
| *Z* | 4 | 4 |
| *Z'* | 0.5 | 0.5 |
| Wavelength / Å | 1.54184 | 0.71073 |
| Radiation type | Cu $K_\alpha$ | Mo $K_\alpha$ |
| $\Theta_{min}$ / ° | 5.351 | 2.457 |
| $\Theta_{max}$ / ° | 77.989 | 30.675 |
| Measured Refl's. | 18518 | 22667 |
| Indep't Refl's | 1994 | 2717 |
| Refl's $I \geq 2\,\sigma(I)$ | 1837 | 2581 |
| $R_{int}$ | 0.0363 | 0.0277 |
| Parameters | 92 | 92 |
| Restraints | 0 | 0 |
| Largest Peak | 0.286 | 0.424 |
| Deepest Hole | -0.162 | -0.132 |
| GooF | 1.065 | 1.071 |
| $wR_2$ (all data) | 0.0865 | 0.0574 |
| $wR_2$ | 0.0847 | 0.0568 |
| $R_1$ (all data) | 0.0343 | 0.0229 |
| $R_1$ | 0.0318 | 0.0215 |

**Table S2.** Crystallographic parameters for the bimetallic compounds **2-Mg** and **2-Mn**

| **Compound** | **2-Mg** | **2-Mn** |
|---|---|---|
| Formula | $C_{26}H_{46}Ir_2MgN_2$ | $C_{26}H_{46}Ir_2MnN_2$ |
| $D_{calc.}$ / g cm$^{-3}$ | 1.792 | 1.849 |
| $m$ / mm$^{-1}$ | 9.052 | 9.380 |
| Formula Weight | 795.36 | 825.99 |
| Color | colorless | yellow |
| Shape | plate-shaped | block-shaped |
| Size / mm$^3$ | 0.35×0.27×0.08 | 0.30×0.17×0.11 |
| $T$ / K | 100.02(10) | 100.00(10) |
| Crystal System | orthorhombic | orthorhombic |
| Flack Parameter | -0.018(8) | -0.014(5) |
| Hooft Parameter | -0.025(5) | -0.012(4) |
| Space Group | $Pna2_1$ | $Pna2_1$ |
| $a$ / Å | 22.9272(4) | 22.9641(3) |
| $b$ / Å | 10.15860(10) | 10.20390(10) |
| $c$ / Å | 12.6604(2) | 12.6636(2) |
| $\alpha$ / ° | 90 | 90 |
| $\beta$ / ° | 90 | 90 |
| $\gamma$ / ° | 90 | 90 |
| $V$ / Å$^3$ | 2948.71(8) | 2967.38(7) |
| $Z$ | 4 | 4 |
| $Z'$ | 1 | 1 |
| Wavelength / Å | 0.71073 | 0.71073 |
| Radiation type | Mo $K_\alpha$ | Mo $K_\alpha$ |
| $\Theta_{min}$ / ° | 2.720 | 3.118 |
| $\Theta_{max}$ / ° | 30.485 | 30.469 |
| Measured Refl's. | 62704 | 62924 |
| Indep't Refl's | 7846 | 7943 |
| Refl's $I \geq 2\,\sigma(I)$ | 7188 | 7488 |
| $R_{int}$ | 0.0585 | 0.0525 |
| Parameters | 294 | 294 |
| Restraints | 1 | 1 |
| Largest Peak | 2.078 | 1.230 |
| Deepest Hole | -1.755 | -0.884 |
| GooF | 1.040 | 1.051 |
| $wR_2$ (all data) | 0.0744 | 0.0445 |
| wR2 | 0.0720 | 0.0438 |
| R1 (all data) | 0.0366 | 0.0241 |
| $R_1$ | 0.0306 | 0.0213 |

**Table S3.** Crystallographic parameters for the bimetallic compounds **3-Mg** and **3-Mn**

| **Compound** | **3-Mg** | **3-Mn** |
|---|---|---|
| Formula | $C_{28}H_{58}IrMgN_2O_4Si$ | $C_{28}H_{58}IrMnN_2O_4Si$ |
| $D_{calc.}$ / g cm$^{-3}$ | 1.395 | 1.448 |
| $m$ / mm$^{-1}$ | 3.916 | 4.229 |
| Formula Weight | 731.36 | 761.99 |
| Color | colorless | dark brown |
| Shape | block-shaped | plate-shaped |
| Size / mm$^3$ | 0.34×0.23×0.16 | 0.27×0.25×0.10 |
| $T$ / K | 100.00(15) | 100.00(10) |
| Crystal System | triclinic | triclinic |
| Space Group | $P$-1 | $P$-1 |
| $a$ / Å | 11.56810(10) | 11.5899(2) |
| $b$ / Å | 12.35950(10) | 12.3899(2) |
| $c$ / Å | 13.04210(10) | 13.0411(2) |
| $\alpha$ / ° | 97.5580(10) | 97.7280(10) |
| $\beta$ / ° | 96.9270(10) | 96.9290(10) |
| $\gamma$ / ° | 107.0920(10) | 107.1100(10) |
| $V$ / Å$^3$ | 1741.73(3) | 1747.98(5) |
| $Z$ | 2 | 2 |
| $Z'$ | 1 | 1 |
| Wavelength / Å | 0.71073 | 0.71073 |
| Radiation type | Mo $K_\alpha$ | Mo $K_\alpha$ |
| $\Theta_{min}$ / ° | 2.936 | 2.932 |
| $\Theta_{max}$ / ° | 30.646 | 30.609 |
| Measured Refl's. | 78010 | 76849 |
| Indep't Refl's | 9492 | 9465 |
| Refl's $I \geq 2\ \sigma(I)$ | 8993 | 8697 |
| $R_{int}$ | 0.0443 | 0.0567 |
| Parameters | 352 | 352 |
| Restraints | 0 | 0 |
| Largest Peak | 1.273 | 1.286 |
| Deepest Hole | -1.046 | -1.343 |
| GooF | 1.061 | 1.145 |
| $wR_2$ (all data) | 0.0430 | 0.0518 |
| $wR_2$ | 0.0426 | 0.0510 |
| $R_1$ (all data) | 0.0199 | 0.0267 |
| $R_1$ | 0.0180 | 0.0233 |

**Table S4.** Crystallographic parameters for compounds **4** and **5-Mn**

| **Compound** | **4** | **5-Mn** |
|---|---|---|
| Formula | $C_{50}H_{34}BF_{21}Ir_2$ | $C_{100}H_{176}Ir_6Mn_8$ |
| $D_{calc.}$ / g cm$^{-3}$ | 1.953 | 1.585 |
| *m* / mm$^{-1}$ | 5.588 | 7.197 |
| Formula Weight | 1428.98 | 2971.12 |
| Color | translucent intense yellow | black |
| Shape | plate | block-shaped |
| Size / mm$^3$ | 0.83×0.67×0.09 | 0.17×0.15×0.08 |
| *T* / K | 149(2) | 100.0(4) |
| Crystal System | monoclinic | monoclinic |
| Space Group | $P2_1/n$ | *P*21/*c* |
| *a* / Å | 11.0362(3) | 22.7271(5) |
| *b* / Å | 18.4322(5) | 12.8289(2) |
| *c* / Å | 24.4821(7) | 23.6110(6) |
| *α* / ° | 90 | 90 |
| *β* / ° | 102.627(3) | 115.276(3) |
| *γ* / ° | 90 | 90 |
| *V* / Å$^3$ | 4859.7(2) | 6225.0(3) |
| *Z* | 4 | 2 |
| *Z'* | 1 | 0.5 |
| Wavelength / Å | 0.71073 | 0.71073 |
| Radiation type | Mo $K_{\alpha}$ | Mo $K_{\alpha}$ |
| $\Theta_{min}$ / ° | 2.190 | 2.285 |
| $\Theta_{max}$ / ° | 30.506 | 30.693 |
| Measured Refl's. | 30405 | 147058 |
| Indep't Refl's | 14773 | 17589 |
| Refl's $I \geq 2\ \sigma(I)$ | 10399 | 14875 |
| $R_{int}$ | 0.0715 | 0.0638 |
| Parameters | 686 | 541 |
| Restraints | 48 | 0 |
| Largest Peak | 3.383 | 5.021 |
| Deepest Hole | -2.725 | -1.021 |
| GooF | 1.085 | 1.046 |
| $wR_2$ (all data) | 0.1491 | 0.1160 |
| $wR_2$ | 0.1278 | 0.1127 |
| $R_1$ (all data) | 0.1001 | 0.0586 |
| $R_1$ | 0.0643 | 0.0487 |

**Table S5.** Crystallographic parameters for compounds **6-Mg**, **6-Mn** and **8-Mn**

| **Compound** | **6-Mg** | **6-Mn** | **8-Mn** |
|---|---|---|---|
| Formula | $C_{115}H_{182}Mg_3N_6O_2$ | $C_{37}H_{58}MnN_2$ | $C_{52}H_{100}Ir_2Mn_2O_{10}Si_2$ |
| $D_{calc.}$ / g cm$^{-3}$ | 1.044 | 1.089 | 1.481 |
| $m$ / mm$^{-1}$ | 0.607 | 0.394 | 4.587 |
| Formula Weight | 1753.59 | 585.79 | 1435.77 |
| Color | colorless | yellow | yellow |
| Shape | block-shaped | plate-shaped | plate-shaped |
| Size / mm$^3$ | 0.24×0.15×0.11 | 0.33×0.28×0.12 | 0.37×0.16×0.07 |
| $T$ / K | 100.01(17) | 100.0(2) | 100.02(10) |
| Crystal System | monoclinic | monoclinic | monoclinic |
| Space Group | $P2_1/c$ | $I2/c$ | $P21/n$ |
| $a$ / Å | 20.23142(18) | 17.087(2) | 12.04110(10) |
| $b$ / Å | 32.4033(2) | 10.64730(10) | 15.0693(2) |
| $c$ / Å | 17.61471(13) | 20.1976(8) | 17.9165(2) |
| $\alpha$ / ° | 90 | 90 | 90 |
| $\beta$ / ° | 104.8870(8) | 103.430(6) | 97.9630(10) |
| $\gamma$ / ° | 90 | 90 | 90 |
| $V$ / Å$^3$ | 11159.98(16) | 3574.1(5) | 3219.62(6) |
| $Z$ | 4 | 4 | 2 |
| $Z'$ | 1 | 0.5 | 0.5 |
| Wavelength / Å | 1.54184 | 0.71073 | 0.71073 |
| Radiation type | Cu$K_a$ | Mo $K_\alpha$ | Mo $K_\alpha$ |
| $\Theta_{min}$ / ° | 3.766 | 2.272 | 2.937 |
| $\Theta_{max}$ / ° | 74.641 | 30.600 | 30.609 |
| Measured Refl's. | 128618 | 77128 | 73095 |
| Indep't Refl's | 22270 | 5087 | 8778 |
| Refl's $I \geq 2\,\sigma(I)$ | 19944 | 4811 | 8100 |
| $R_{int}$ | 0.0319 | 0.0384 | 0.0307 |
| Parameters | 1117 | 200 | 321 |
| Restraints | 0 | 32 | 0 |
| Largest Peak | 2.224 | 0.407 | 1.117 |
| Deepest Hole | -0.692 | -0.557 | -0.645 |
| GooF | 1.078 | 1.248 | 1.016 |
| $wR_2$ (all data) | 0.3033 | 0.1118 | 0.0440 |
| $wR_2$ | 0.2970 | 0.1111 | 0.0432 |
| $R_1$ (all data) | 0.1176 | 0.0532 | 0.0210 |
| $R_1$ | 0.1111 | 0.0509 | 0.0179 |

**Table S6.** Crystallographic parameters for the monometallic Mg and Mn complexes obtained by double protonolysis of compounds **2-Mg** and **2-Mn** with 2 eq. of tris(*tert*-butoxy)silanol

| **Compound** | **[Mg(tmeda)(OSi{O*t*Bu}$_3$)$_2$]** | **[Mn(tmeda)(OSi{O*t*Bu}$_3$)$_2$]** |
|---|---|---|
| Formula | $C_{30}H_{70}MgN_2O_8Si_2$ | $C_{30}H_{70}MnN_2O_8Si_2$ |
| $D_{calc.}$ / g cm$^{-3}$ | 1.078 | 1.133 |
| $m$ / mm$^{-1}$ | 1.271 | 0.423 |
| Formula Weight | 667.37 | 698.00 |
| Color | colorless | colorless |
| Shape | block-shaped | block-shaped |
| Size / mm$^3$ | 0.29×0.26×0.15 | 0.45×0.23×0.17 |
| $T$ / K | 100.03(11) | 100.0(3) |
| Crystal System | orthorhombic | triclinic |
| Space Group | *Pbcm* | *P*-1 |
| $a$ / Å | 8.88615(5) | 18.4577(2) |
| $b$ / Å | 16.88944(10) | 21.7020(3) |
| $c$ / Å | 27.41111(14) | 21.8833(3) |
| $\alpha$ / ° | 90 | 89.7820(10) |
| $\beta$ / ° | 90 | 77.0900(10) |
| $\gamma$ / ° | 90 | 73.7370(10) |
| $V$ / Å$^3$ | 4113.92(4) | 8186.12(19) |
| $Z$ | 4 | 8 |
| $Z'$ | 0.5 | 4 |
| Wavelength / Å | 1.54184 | 0.71073 |
| Radiation type | Cu $K_a$ | Mo $K_a$ |
| $\Theta_{min}$ / ° | 3.225 | 3.201 |
| $\Theta_{max}$ / ° | 78.998 | 30.701 |
| Measured Refl's. | 41751 | 67482 |
| Indep't Refl's | 4374 | 67482 |
| Refl's $I \geq 2\,\sigma(I)$ | 4183 | 47162 |
| $R_{int}$ | 0.0207 | − (twinned crystal) |
| Parameters | 230 | 1638 |
| Restraints | 0 | 0 |
| Largest Peak | 0.253 | 1.212 |
| Deepest Hole | -0.292 | -0.580 |
| GooF | 1.061 | 1.045 |
| $wR_2$ (all data) | 0.0800 | 0.1446 |
| $wR_2$ | 0.0790 | 0.1256 |
| $R_1$ (all data) | 0.0289 | 0.0920 |
| $R_1$ | 0.0279 | 0.0532 |

# References


(1) Sheldrick, G. M. SHELXT − Integrated Space-Group and Crystal-Structure Determination. *Acta Cryst. A* **2015**, *71* (1), 3–8. https://doi.org/10.1107/S2053273314026370.

(2) Dolomanov, O. V.; Bourhis, L. J.; Gildea, R. J.; Howard, J. A. K.; Puschmann, H. OLEX 2: A Complete Structure Solution, Refinement and Analysis Program. *J. Appl. Crystallogr.* **2009**, *42* (2), 339–341. https://doi.org/10.1107/S0021889808042726.

(3) Sheldrick, G. M. Crystal Structure Refinement with SHELXL. *Acta Cryst. C* **2015**, *71* (1), 3–8. https://doi.org/10.1107/S2053229614024218.

(4) Hill, M. S.; Johnson, A. L.; Manning, T. D.; Molloy, K. C.; Wickham, B. J. Single-Source AACVD of Composite Cobalt-Silicon Oxide Thin Films. *Inorg. Chim. Acta* **2014**, *422*, 47–56. https://doi.org/10.1016/j.ica.2014.07.045.

(5) Werner, D.; Anwander, R. Unveiling the Takai Olefination Reagent via Tris(*Tert*-Butoxy)Siloxy Variants. *J. Am. Chem. Soc.* **2018**, *140* (43), 14334–14341. https://doi.org/10.1021/jacs.8b08739.
